# Robustness and Evolvability of the Human Signaling Network

**DOI:** 10.1371/journal.pcbi.1003763

**Published:** 2014-07-31

**Authors:** Junil Kim, Drieke Vandamme, Jeong-Rae Kim, Amaya Garcia Munoz, Walter Kolch, Kwang-Hyun Cho

**Affiliations:** 1 Department of Bio and Brain Engineering, Korea Advanced Institute of Science and Technology (KAIST), Yuseong-gu, Daejeon, Republic of Korea; 2 Systems Biology Ireland, University College Dublin, Dublin, Ireland; 3 Department of Mathematics, University of Seoul, Seoul, Republic of Korea; 4 Conway Institute of Biomolecular and Biomedical Research, University College Dublin, Dublin, Ireland; 5 School of Medicine and Medical Science, University College Dublin, Dublin, Ireland; University of Virginia, United States of America

## Abstract

Biological systems are known to be both robust and evolvable to internal and external perturbations, but what causes these apparently contradictory properties? We used Boolean network modeling and attractor landscape analysis to investigate the evolvability and robustness of the human signaling network. Our results show that the human signaling network can be divided into an evolvable core where perturbations change the attractor landscape in state space, and a robust neighbor where perturbations have no effect on the attractor landscape. Using chemical inhibition and overexpression of nodes, we validated that perturbations affect the evolvable core more strongly than the robust neighbor. We also found that the evolvable core has a distinct network structure, which is enriched in feedback loops, and features a higher degree of scale-freeness and longer path lengths connecting the nodes. In addition, the genes with high evolvability scores are associated with evolvability-related properties such as rapid evolvability, low species broadness, and immunity whereas the genes with high robustness scores are associated with robustness-related properties such as slow evolvability, high species broadness, and oncogenes. Intriguingly, US Food and Drug Administration-approved drug targets have high evolvability scores whereas experimental drug targets have high robustness scores.

## Author Summary

Biological systems are known to be robust and evolvable to internal mutations and external environmental changes. What causes these apparently contradictory properties? This study shows that the human signaling network can be decomposed into two structurally distinct subgroups of links that provide both evolvability to environmental changes and robustness against internal mutations. The decomposition of the human signaling network reveals an evolutionary design principle of the network, and also facilitates the identification of potential drug targets.

## Introduction

Organisms have evolved so that their networks are robust against the effects of mutations, but evolvable in response to environmental changes [1]–[4]. Genetic mutations can profoundly change network structures, so mutational robustness of a network indicates how well the network can preserve its own dynamic behavior upon changes to its structure. In a similar way, evolvability of a network represents how well a network can produce appropriate dynamic behavior in response to environmental changes. Although robustness and evolvability are apparently opposite notions, they are simultaneously implicit in biological organisms. There are three main research results on mutational robustness and evolvability. First, mutational robustness facilitates evolvability as high mutational robustness increases the diversity of genotypes that can evolve [5]–[7]. Second, biological networks have evolved to have scale-free structures [8] and highly optimized tolerance (HOT) structures [9] so as to increase mutational robustness. Third, biological systems have evolved to possess modular structures [10]–[12], critical regime [2], [13], hub nodes [14], [15], and hierarchical structures [15] so as to simultaneously increase mutational robustness and evolvability. These investigations mainly focused on either revealing the relationship between mutational robustness and evolvability or unraveling the structural characteristics of biomolecular regulatory networks which have evolved to increase robustness and evolvability.

Although a number of studies have been done on mutational robustness and evolvability of the biomolecular regulatory networks [2], [5]–[15], many questions still remain unsolved. For instance, the evolutionary design principles by which the mutational robustness and evolvability are implemented in biomolecular regulatory networks are poorly understood. For this purpose, we need to identify not only the network components and their molecular interactions but also the dynamic properties of the network.

Previous studies have shown that signaling networks can effectively be analyzed by considering the cellular phenotype as a high-dimensional state attractor [16]–[21]. An attractor is a mathematical concept representing a stable steady state or limit cycle (a repeating sequence of states) adopted by a dynamic system, in this case a signaling network [16]–[21]. Based on this concept a signaling network is mapped into an attractor landscape, where each point in this landscape represents one state of the network defined by a set of state values containing the activity states of all signaling proteins in the network [16]–[21]. Although an attractor landscape of a signaling network is composed of various attractors, cellular behavior typically reaches a dominant stable state known as “primary attractor”, which represents the normal cellular state or phenotype [16]–[21]. The set of states, which converge to an attractor, is called the “basin of attraction” and the primary attractor has the biggest basin of attraction [16]–[21].

In this paper, we show that the human signaling network consists of a subgroup of interactions for mutational robustness and the other subgroup of interactions for evolvability. For this purpose we used an integrated human signaling network constructed by Helikar *et al.* [22], where the connections between nodes (edges) are described by well-characterized Boolean logics derived from mechanistic data in the biochemical literature, which based on a set of logical rules specify whether a connection exists or not. This network model is composed of representative signal transduction pathways regulated by three major receptor families including receptor tyrosine kinases, G protein-coupled receptors, and integrins. It was shown that the Boolean dynamic model of this network has the ability to replicate known qualitative behaviors of the actual human signaling network. Based on the Boolean network model, we first identified an attractor landscape of the model, and then we decomposed the network into two subgroups of interactions: the evolvable core, which preserves the basin of the primary attractor in state space, and the robust neighbor, which has no influence on the basin of the primary attractor. Decomposition of the network elucidated that the evolvable core has more scale-freeness than the robust neighbor and that the robust neighbor contributes to reducing the characteristic path length of the evolvable core, thereby constituting the HOT structure. We validated the theoretical predictions related to the different effects of perturbations in the evolvable core compared to the robust neighbor through biochemical experiments. Our network decomposition analysis further indicates that the genes with high evolvability score are associated with evolvability-related properties whereas those with high robustness score are associated with robustness-related properties. Intriguingly, US Food and Drug Administration (FDA)-approved drug targets have high evolvability score whereas experimental drug targets (targets of drugs in the pipeline or not yet approved by the FDA) [23] have high robustness score. Thus, the decomposition of a biomolecular regulatory network into an evolvable core and a robust neighbor can not only reveal the evolutionary design principle of the network, but also help identifying potential drug targets.

## Results

### Decomposition of the human signaling network

**Figure 1:**
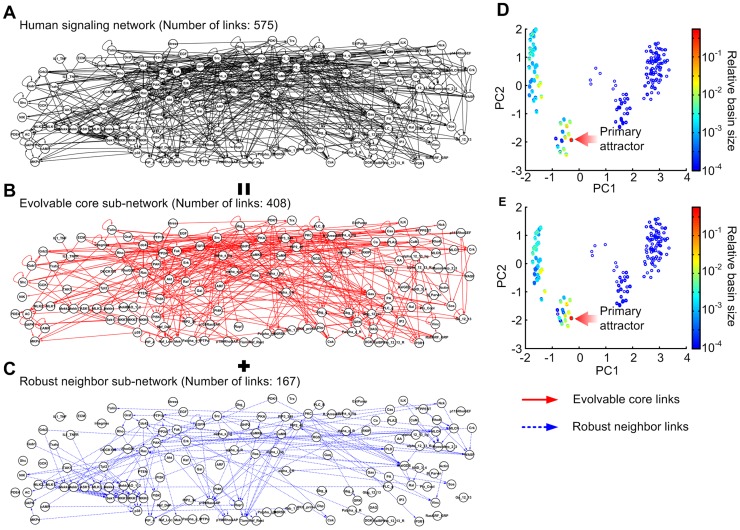
Decomposition of the human signaling network. (**A**) Human signaling network. (**B**) Evolvable core. (**C**) Robust neighbor. (**D**) Attractor landscape of the human signaling network. (**E**) Attractor landscape of the evolvable core.

The attractor landscape is a useful representation of phenotypes of biological systems [2]. Hence we defined the two subgroups of interactions (the evolvable core and robust neighbor) of a biomolecular regulatory network based on the attractor landscape (see Materials and Methods for the definition of these subgroups of interactions). In order to decompose the human signaling network (Fig. 1A) into the evolvable core and robust neighbor, we first identified the attractor landscape of the network through Boolean simulation. Since the human signaling network consists of 139 nodes, we would have to calculate transitions between 2^139^ states to obtain its attractor landscape, which is unfeasible. Therefore, we used 10,000 randomly selected initial states to identify the approximated attractor landscape (see Materials and Methods). The reason why we used the sampling size 10,000 is because it is feasible, and because we could show that for sample sizes 10–100 fold bigger than 10,000 the distributions of estimated (relative) basin sizes are very similar (Fig. S1). From this random sampling approach, we obtained an approximated landscape of the human signaling network. From the 10,000 initial states, we obtained 135 attractors and found one primary attractor whose basin contained approximately 56% out of the 10,000 initial states (Fig. S1A). This primary attractor was a limit cycle composed of a repeating sequence of six states (Table S1). In these six states, 123 nodes were ‘OFF’ and the remaining 16 nodes were ‘ON’ at least once in their cyclic state transitions. The sub-network composed of these 16 nodes and their interactions consists of three separate modules: a module for phosphatidylinositol signaling, a module for Raf activation (composed of three inactivated forms of Raf and PP2A (Protein serine/threonine Phosphatase 2A)), and a module for PKC (Protein Kinase C) activation (composed of PKC_primed which is an inactivated form of PKC) (Fig. S2). The ‘ON’ nodes in the primary attractor are related to precursors of second messengers or inactive forms of kinases. In other words, the primary attractor can be considered as a ‘ready’ state of the signaling network, which might be the nominal condition of cell signaling [24]–[28].

Next, we developed an algorithm for the decomposition of the human signaling network (see Materials and Methods), which allowed us to identify the evolvable core with 408 edges (Fig. 1B) and the robust neighbor with 167 edges (Fig. 1C). The lists of links in the evolvable core and robust neighbor are provided in Table S2 and Table S3, respectively. We obtained similar results when using different random seeds of initial states (Fig. S3A) and deletion order (Fig. S3B). In order to compare the attractor landscape of the original human signaling network and its evolvable core, we projected all the obtained attractor states, which correspond to 139-dimensional vectors, onto a 2-dimensional plane using principle component analysis (PCA). Fig. 1D shows the projected landscape of the 135 attractors of the original network. Using the same 10,000 initial states as used to find the attractors of the original network, we obtained 106 attractors for the evolvable core. Then we projected all the obtained attractor states onto the same plane in Fig. 1D, after applying the same linear transformation as in the PCA analysis of the original network. Fig. 1E shows this projected landscape of attractors of the evolvable core. The attractor landscape of the original network and that of the evolvable core are very similar despite the fact that the evolvable core was obtained by removing edges whose deletion did not change the landscape of the primary attractor only. Furthermore, the approximated relative basin sizes of each attractor were also similar (Figs. 1D and E). These results imply that the landscape of the evolvable core largely represents the landscape of the original human signaling network.

### Perturbation experiments of the evolvable core and robust neighbor

**Figure 2:**
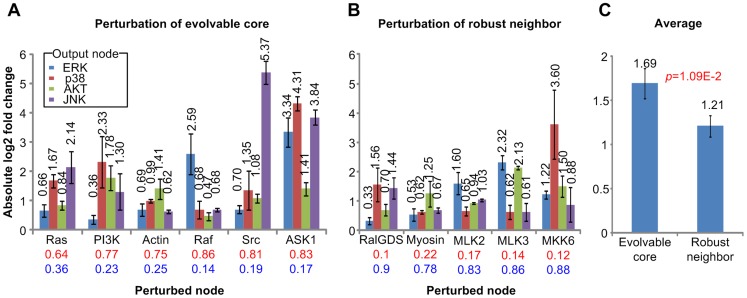
Perturbation experiments of the evolvable core and robust neighbor. (**A**) Average of absolute log2 fold change of each output node activity over three repetitions obtained from each perturbation experiments of the evolvable core. (**B**) Average of absolute log2 fold change of each output node activity over three repetitions obtained from each perturbation experiments of the robust neighbor. (**C**) Average of all the experimental results (three repetitions×four output nodes×eight perturbation experiments) with respect to perturbation of the evolvable core elements and average of all the experimental results (three repetitions×four output nodes×five perturbation experiments) with respect to perturbation of the robust neighbor elements. Error bars denote the standard errors of the average values. The red numbers and blue numbers in Figs. 2A and 2B denote the evolvability scores and the robustness scores, respectively.

We wanted to experimentally validate the theoretical prediction that perturbations in the evolvable core have a stronger effect on the network than perturbations in the robust neighbor. Therefore, we carried out a series of biochemical experiments where we induced perturbations through chemical inhibition or overexpression of the nodes with high evolvability score and those with high robustness score (see Materials and Methods and Table S4 for the definition of these scores), and compared the phosphorylation of four network outputs: ERK, Akt, p38 and JNK (Figs. 2 and S4). To perturb the nodes with high evolvability score, we overexpressed the constitutive active HRasV12 mutant (Ras perturbation), the HRasV12C40 mutant (PI3K perturbation), Raf1 (Raf perturbation), Src, and ASK1; and we carried out chemical inhibition with blebbistatin (Actin perturbation). To perturb the nodes with high robustness score, we overexpressed the HRasV12G37 mutant (RalGDS perturbation), MLK2, MLK3, and MKK6; and we carried out chemical inhibition with the drug ML7 (Myosin perturbation). All kinases were GFP tagged, and each experiment was carried out in triplicate. A representative western blot is shown in Fig. S4. To facilitate comparison between the different types of experiments all measurements were quantified and normalized to the value of the respective controls (untreated cells or cells expressing a control plasmid). The results show that the overall normalized perturbation effect of the evolvable core is higher than that of the robust neighbor (Figs. 2A, 2B and S5). The average of all the perturbation effects for the evolvable core was significantly higher (*p*-value = 1.09E-2) than that for the robust neighbor (Fig. 2C).

### Topological properties of the evolvable core and robust neighbor sub-network

**Figure 3:**
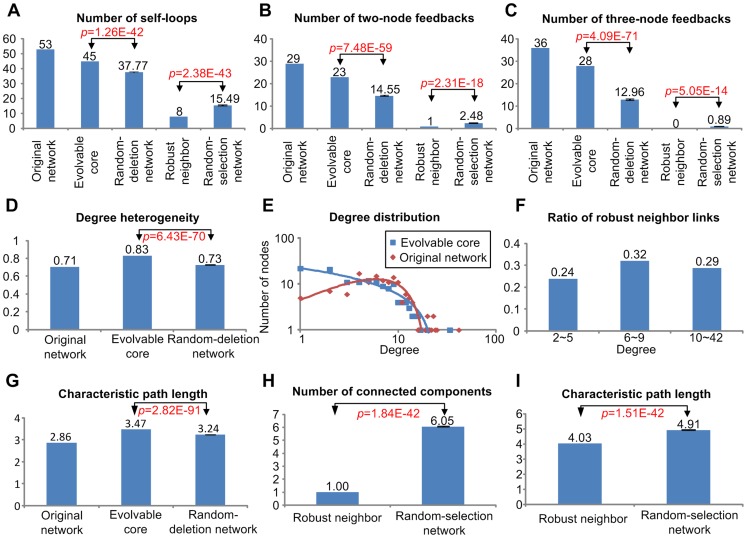
Topological characteristics of the evolvable core and robust neighbor sub-network. (**A**) Number of self-loops. (**B**) Number of two-node feedbacks. (**C**) Number of three-node feedbacks of the original network, evolvable core sub-network, random-deletion sub-network, robust neighbor sub-network, and random-selection sub-network. (**D**) Degree heterogeneity of the original network, evolvable core sub-network, and random-deletion sub-network. (**E**) Degree distribution of the original network and evolvable core sub-network. (**F**) The ratio of robust neighbor links to the whole links for the low-degree, middle-degree, and high-degree nodes, respectively. (**G**) Characteristic path length of the original network, evolvable core sub-network, and random-deletion sub-network. (**H**) Number of connected components of the robust neighbor and random-selection sub-network. (**I**) Characteristic path lengths of the robust neighbor and random-selection sub-network. Error bars denote the standard errors of the average values.

In the previous subsections, we showed that the human signaling network could be decomposed into an evolvable core and a robust neighbor. The attractor landscape of the evolvable core is very similar to that of the original network, and the edges of the robust neighbor do not affect the landscape of the original network. How do the two sub-networks differ in terms of structure? The interlinked structure of feedback loops in a network is an important factor determining the characteristics of the attractor landscape, such as the number of attractors and the distribution of basin sizes [29], [30]. Hence, we first compared the numbers of self-feedback loops (Fig. 3A), two-node feedback loops (Fig. 3B), and three-node feedback loops (Fig. 3C) in the evolvable core sub-network to the numbers of these loops in similar random edge-deleted sub-networks; and also compared the numbers of these loops in the robust neighbor sub-network to the numbers of these loops in similar random edge-selected sub-networks (see Materials and Methods for the definition of these sub-networks). For this purpose we constructed 100 random-deletion sub-networks by taking the human signaling network and randomly deleting 167 edges; and 100 random-selection sub-networks by taking sub-networks composed of 167 edges randomly selected from the human signaling network. Subsequently, we calculated the average number of feedback loops in the random-deletion sub-networks and compared them to the numbers in the evolvable core sub-network; and calculated the average number of feedback loops in the random-selection sub-networks and compared them to the numbers in the robust neighbor sub-network. We found that the evolvable core sub-network contains significantly more feedback loops compared to the random-deletion sub-networks, whereas the robust neighbor sub-network contains significantly less feedback loops compared to the random-selection sub-networks (Figs. 3A, 3B, and 3C). We obtained similar results when using different random seeds of initial states (Figs. S5A, S5C, and S5E) and deletion order (Figs. S5B, S5D and S5F).

Scale-freeness is one of the representative characteristics of biological networks. We calculated the degree heterogeneity and the degree distribution [31], [32] as a measure of this scale-freeness. As a result, we found that the degree heterogeneity of the evolvable core sub-network is significantly higher than that of random-deletion sub-networks (Fig. 3D). The degree distribution of the original network is similar to that of an Erdös random network [33] which has many middle-degree nodes, whereas the degree distribution of the evolvable core sub-network is similar to that of a scale-free network [33] which has much more low-degree nodes (Fig. 3E). This implies that many middle-degree nodes were deleted during the link-deletion process that identified the evolvable core. In fact, we verified that the ratio of robust neighbor links to the whole links for the middle-degree (from 6 to 9) nodes is higher than those for the low-degree (from 2 to 5) and high-degree (from 10 to 42) nodes (Fig. 3F). We obtained similar results using different random seeds of initial states (Figs. S6A and S6C) and deletion order (Figs. S6B and S6D).

It is well-known that biological networks which transfer information such as cell signaling pathways satisfy the small-world property as well as scale-freeness [34]. In order to explore the small-world property of the evolvable core sub-network, we compared the characteristic path length [32] of the evolvable core sub-network to 100 random-deletion sub-networks. We found that the characteristic path length of the evolvable core sub-network is larger than those of random-deletion sub-networks (Fig. 3G). This implies that appending the robust neighbor to the evolvable core increases the small-world property. We obtained similar results using different random seeds of initial states (Fig. S6E) and deletion order (Fig. S6F). In order to verify this, we also compared the network structures of the robust neighbor sub-network to the 100 previously mentioned random-selection sub-networks. The result shows that the number of connected components (Fig. 3H) and the characteristic path length (Fig. 3I) of the robust neighbor sub-network are significantly smaller than those of random-selection sub-networks. We obtained similar results using different random seeds of initial states (Figs. S7A and S7C) and deletion order (). In summary, the robust neighbor sub-network contains many middle-degree nodes that are closely connected to each other. Hence we conclude the structure of the robust neighbor sub-network is similar to the HOT structure [9], [35], and has been evolutionarily designed to be robust to changes or a targeted attack.

### Genetic properties of the network nodes versus evolvability and robustness scores

**Figure 4:**
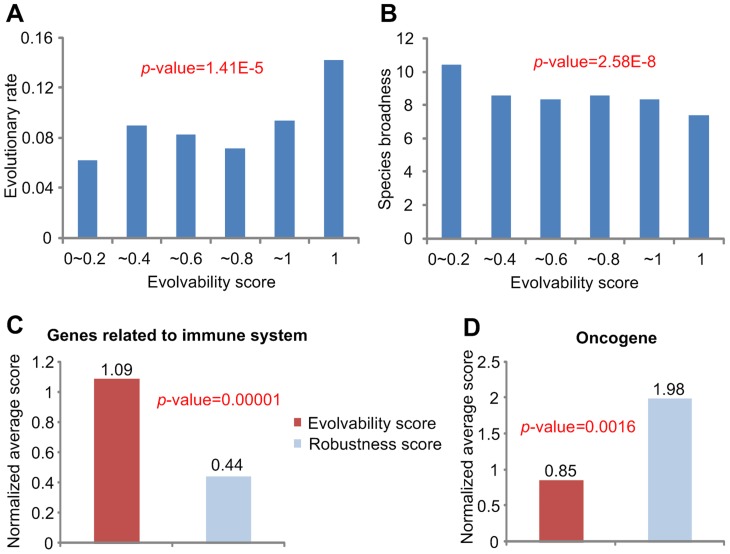
Genetic properties of the network nodes in terms of evolvability and robustness scores. (**A**) The correlation between evolutionary rate and evolvability score. (**B**) The correlation between species broadness and evolvability score. (**C**) The normalized average evolvability and robustness scores of the genes related to immune system. (**D**) The normalized average evolvability and robustness scores of the oncogenes.

The evolvable core is defined by the minimal subgroup of interactions that preserves the attractor landscape and the robust neighbor is defined by the subgroup of interactions satisfying that deletion of any link in the subgroup of interactions does not affect the attractor landscape. From this definition, we speculated that a link perturbation on the evolvable core could induce a new phenotype with higher probability than that on the robust neighbor. In order to confirm this conjecture, we investigated the relationship between the evolutionary rate and the evolvability score for each node, and found a significant positive correlation between them (Fig. 4A). Furthermore, we found that the species broadness is significantly negatively correlated with the evolvability score (Fig. 4B). This result implies that mutations in the evolvable core can induce new phenotypes more frequently, since the mutation of genes with high evolutionary rates can facilitate positive selection [36] and genes with low species broadness result from rapid evolution [37]. We again obtained similar results using different random seeds of initial states (Fig. S8A and S8C) and deletion order (Fig. S8B and S8D).

Furthermore, we looked at genes related to the immune system and oncogenes. The immune system is known to rapidly evolve in order to cope with rapidly evolving pathogens [38], [39]. Oncogenes denote genes that promote cancer when mutated or overexpressed. Cancer is a system which utilizes some of the robustness mechanisms of normal tissues and further evolves them to become more robust due to the greatly enhanced ability of generating genetically heterogeneous cells that increase the population fitness under selection [40]. Therefore, the genes related to the immune system might have higher evolvability score than other genes whereas the oncogenes might have higher robustness score than the other genes. As expected, the genes related to immune system have a high normalized average evolvability score (Fig. 4C), whereas oncogenes have a high normalized average robustness score (Fig. 4D). These findings support the notion that the evolvable core is related to evolvability, and the robust neighbor is related to robustness in terms of biological data. We obtained similar results using different random seeds of initial states (Figs. S9A and S9C) and deletion order (Figs. S9B and S9D).

### Drug targets

**Figure 5:**
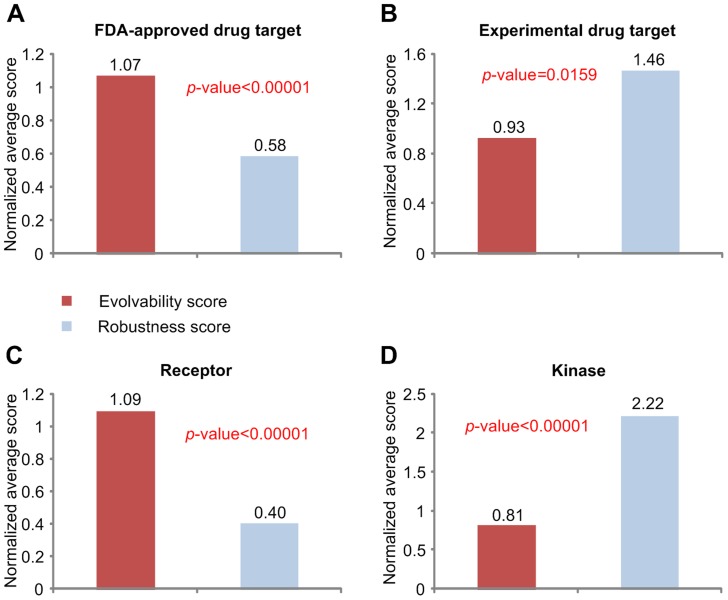
The relationship between drug targets and evolvable core or robust neighbor. The normalized average evolvability and robustness scores of the genes related to FDA-approved drug targets (**A**), experimental drug targets (**B**), receptors (**C**), and kinases (**D**).

Because a link perturbation on the evolvable core could be more effective in changing the cellular phenotype than a link perturbation on the robust neighbor, we can speculate that drug targets might have higher evolvability scores than non-drug targets. We found that the FDA-approved drug targets have a high normalized average evolvability score (Fig. 5A). Similarly, we can expect that the experimental drug targets might have a high normalized average evolvability score. Surprisingly, we found that the experimental drug targets have a high normalized average robustness score (Fig. 5B). Since many drugs have multiple target proteins, we considered combination of targets for all the FDA-approved drugs and the union of targets of all the experimental drugs. The overlap between 1330 targets of FDA-approved drugs and 765 targets of experimental drugs is only 297. To show that this overlap does not influence our result on the relationship between evolvability score and drug target, we further analyzed the FDA-approved drug targets that are not experimental drug targets and the experimental drug targets that are not FDA-approved drug targets, and obtained the same results (Fig. S10). Moreover, we have analyzed the evolvability scores of all the targets of each multi-target drug and calculated the standard deviation of the scores. We found that the average of the standard deviations for all multi-target drugs (0.0557, see Table S5) is much smaller than the standard deviation of the evolvability scores of all the nodes in the network (0.298). This indicates that most of the targets are still included either in the evolvable core (for the FDA-approved drugs) or in the robust neighbor (for the experimental drugs) and mixed inclusion is uncommon even for the multi-target drugs.

Why do FDA-approved drug targets and experimental drug targets have such contrasting scores? To answer this question, we investigated the distribution of receptors and kinases since most FDA-approved drug targets are membrane proteins such as receptors whereas the experimental drug targets also include proteins localized in various cellular compartments [23]. Interestingly, we found that the receptors have a high normalized average evolvability score (Fig. 5C) whereas the kinases have a high normalized average robustness score (Fig. 5D). This implies that the deletion of a link connected to a receptor is more likely to significantly change the cellular phenotype than the deletion of a link connected to a kinase. It also explains why FDA-approved drug targets and experimental drug targets have such contrasting scores and why they have different cellular compartmental distributions [23]. We obtained similar results using different random seeds of initial states (Figs. S11A, S11C, S11E, and S11G) and deletion order (Figs. S11B, S11D, S11F, and S11H).

## Discussion

Here we show that the human signaling network can be decomposed into an evolvable core and robust neighbor by analyzing the attractor landscape. We also show that the two subgroups of interactions are different in terms of structure and biological meaning. We further validated salient properties of and predicted associations with the evolvable core and robust neighbor experimentally through specific chemical inhibition or overexpression of wild-type and mutant proteins. Like any model our model is not a one-to-one description of the real biological network but a simplified abstraction that can explain general network properties. Thus, we would not expect that every detail can be experimentally confirmed; this even is rarely possible in classic biochemical experiments which test only one or few components of a network. Thus, the experimental work has to be taken as a validation of the general properties of the network, and viewed in the context of the overall results. The experimental results simply add another piece of information to the usefulness of the approach to elaborate network structures with different properties through modeling. One remarkable point in Fig. 2A is that the perturbation effect of ASK1 was most significant. This is particularly meaningful if we consider the following facts: (i) Only ASK1 among the six perturbed evolvable core nodes in the experiments is connected to all of the four output nodes through evolvable core links; (ii) The normalized proportion of the paths in the evolvable core from ASK1 to each output node over such paths in the original network is higher than those of the other five perturbed evolvable core nodes, which means that most of the paths from ASK1 to output nodes remain invariant during the network reduction to evolvable core (Table S6). Hence, the experimental result in Fig. 2A is meaningful even though it cannot be a full experimental validation of the simulation results.

**Figure 6:**
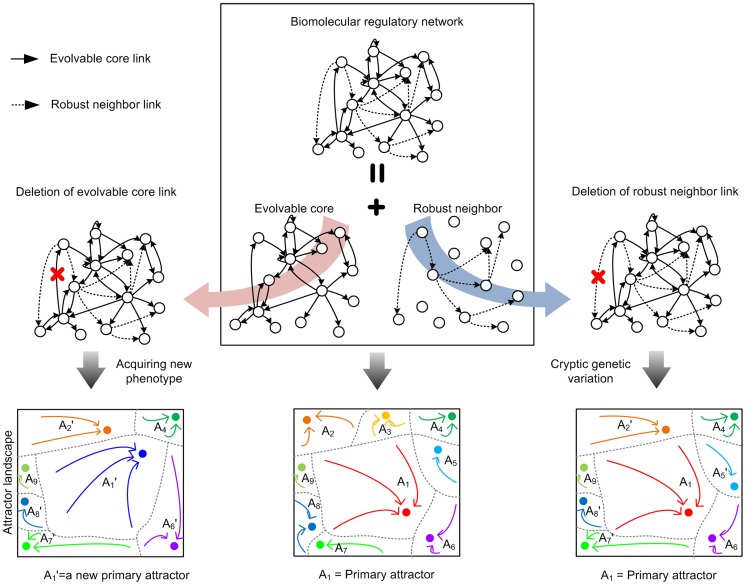
A conceptual framework of the evolvable core and robust neighbor in biomolecular regulatory networks. This figure shows that deletion of an evolvable core link causes acquiring a new phenotype, whereas deletion of a robust neighbor link causes cryptic genetic variation for the biological system represented by the attractor landscape.

The proposed concept and analysis can be applied to any other biomolecular regulatory network that was shaped by evolution. In the conceptual framework of the attractor landscape, deletion of a robust neighbor link causes cryptic genetic variation [6], whereas deletion of an evolvable core link changes the phenotype of the biological system represented by the attractor landscape (Fig. 6). Hence, a molecule which has many robust neighbor links would have robustness-related properties, whereas a molecule which has many evolvable core links would have evolvability-related properties as we found in the human signaling network.

Wagner [41] showed that genotypic robustness and genotypic evolvability share an antagonistic relationship, whereas phenotypic robustness promotes phenotypic evolvability. In this regard, the coexistence of evolvable core and robust neighbor in the human signaling network implies that both phenotypic robustness and phenotypic evolvability are reflected on the human signaling network, since the concepts of evolvable core and robust neighbor of the human signaling network are related to phenotypic evolvability and phenotypic robustness, respectively.

One might consider that the concept of ‘robust neighbor link’ is similar to that of ‘redundant link’ in the context of canalizing function, which is a function of multiple input variables with the property that one of its inputs can solely determine the output value regardless of other inputs [20], [42], [43] (see Fig. S12A). However, they are different because the robust neighbor links can be identified by considering global dynamics whereas the redundant links can only be identified by examining the local relationship between the regulatory inputs and the resulting output of a particular node. To further clarify this, we determined and compared both robust neighbor links and redundant links in our example network. We identified 325 redundant links and found that 192 links out of these 325 redundant links are evolvable core links, which are not redundant when considering the attractor landscape (i.e. not robust neighbor links) (Fig. S12B and Table S7).

Aldana *et al.* [2] defined that a network is evolvable if, as a result of perturbations, new attractors emerge. On the other hand, we defined that a network is evolvable if, as a result of perturbations, the attractor landscape is significantly changed in terms of the primary attractor. To investigate the relationship between these two concepts, we considered 408 sub-networks obtained by deleting the 408 evolvable core links one by one. By simulating each of the 408 sub-networks, 3,500 new attractors were obtained from 10,000 initial states that were included within the basin of the primary attractor of the original network. We found that about 88% of the 3,500 new attractors are not the attractors of the original network. This implies that our concept of evolvability is closely related to the concept of evolvability suggested by Aldana *et al* [2]. However, our concept of evolvability is broader and more inclusive in that a network is evolvable if, as a result of perturbations, an initial state which was included in the basin of attraction of the primary attractor of the original network converges to any of other attractors of the original network or a new one. In the literature, multiple definitions of evolvability were suggested [41], [44]–[51]. In general, a system is said to be evolvable if the genotypic variation in the system can produce heritable phenotypic variation [41]. We think that the difference among the multiple definitions is caused by the different definition of the phenotypic variation. Aldana *et al.* considered the emergence of a new attractor as phenotypic variation. On the other hand, we considered the variation of attractor landscape as phenotypic variation since phenotypic variation includes not only the emergence of new attractors but also the transition between attractors [16], [17], [52], [53].

In this study, we identified the evolvable core and robust neighbor of the human signaling network on the basis of its inherent network dynamics with all the state values of input nodes (i.e. nodes without any incoming link) set to ‘OFF’ and synchronously updating the Boolean functions. To examine whether this result might depend on the input conditions or asynchronous update of Boolean functions, we further carried out extensive simulations for various input conditions and asynchronous update of Boolean functions. This also links the biochemical data better with the simulations, as to see activation, and subsequent differences in activation, of the measured output nodes stimulation with growth factors, such as serum, is necessary. It turns out that the decomposition into the evolvable core and robust neighbor does not depend on the input conditions or synchronous/asynchronous update of Boolean functions, and that the evolvable core and robust neighbor are mostly invariant and do not much depend on such conditions (Figs. S13 and S14) even though the primary attractor might change (Tables S8-S18). This change in primary attractor upon different input conditions makes biological sense, as specific parts of the network are switched ON, in addition to the nodes that are already switched ON with all input nodes ‘OFF’. In other words, the network switches from a ‘ready’ to an ‘active’ state.

Helikar *et al.* [22] showed that there is an emergent function of information processing in the human signaling network. We have further investigated whether such an emergent function is preserved in the evolvable core and found that it is well-preserved (see Fig. S15 and Table S19). This suggests that the evolvable core might be the minimal structure with the complexity that can create such an emergent function.

Previous research suggested that biological networks have evolved to have scale-free [8] and HOT [9] structures so as to increase mutational robustness. However, these studies could not unravel the dynamic characteristics underlying the mutational robustness of the biological networks since they only focused on topological characteristics. Our results about the topological difference such as degree heterogeneity of the evolvable core and low characteristic path length of the robust neighbor, shed light on the previous results in terms of network dynamics that explain the eventual state transition of molecular components in the network in a collective way since we used attractor landscape analysis to decompose the network into the two subgroups of interactions. Furthermore, we show that the two subgroups of interactions are different in terms of biological meaning as well as topological characteristics. Even though we divided the network into the two subgroups of interactions based on the Boolean simulation only, the two subgroups of interactions are distinguished from each other in terms of many biological properties such as evolutionary rate, species broadness, and relationships to the immune system, oncogenes, FDA-approved drug targets, experimental drug targets, receptors, and kinases. To examine the potential generality of our result, we first have analyzed another large-scale signaling network example (JAK/STAT signaling network) obtained from the curated signaling pathway database SignaLink [54]. For this example, we have also identified and analyzed the evolvable core and robust neighbor on the basis of Boolean modeling with the logical rules adopted from Li *et al.* [55]. Secondly, we have analyzed another curated logic model (keratinocyte signaling network) [56]. As a result, we found that the evolvable cores and robust neighbors of the JAK/STAT signaling network and the keratinocyte signaling network show consistent results in terms of genetic properties (evolutionary rate, species broadness, relationship with immune system, and relationship with oncogene, see Fig. S16 and S17). This segregation will be useful for understanding large-scale genomic data, which are now being generated, by predicting which mutations or gene deletions are likely to affect the phenotype. Moreover, we could validate the existence of the evolvable core and robust neighbor through biological experiments.

In our previous research about network kernels [37], we showed that a signaling network can be divided into a kernel and non-kernel. The kernel represents a part that preserves transient dynamics, whereas the evolvable core here represents a part that preserves steady state dynamics. Although these two concepts seem to be similar in terms of preserving some dynamic behavior, they are very different in terms of evolutionary rates and drug targets. Further studies will be needed to unravel the relationship between the kernel and the evolvable core of various biological networks.

## Materials and Methods

### A Boolean network model for the human signaling network

We adapted the Boolean network model of the human signaling network [22] composed of 139 nodes and 575 links (Fig. 1A). In the Boolean network model, each node is associated with a logic table that determines the state of the node for a given input node set [16]–[21], except the nodes without any incoming link [22]. Network dynamics were simulated by updating all the Boolean functions simultaneously from the initial state to the corresponding final attractor state, where a network state is a collective binary representation of all node variables [16]–[21]. The nodes without any incoming link can be considered as input nodes of the network such as ligands of the signaling network. We fixed the state values of those nodes as ‘OFF’ at each time step since we wanted to analyze nominal dynamics of the system without any external input signal.

### Evolvable core (robust neighbor), evolvable core (robust neighbor) sub-network, and evolvability (robustness) score

The evolvable core of a network is defined by the subgroup of interactions satisfying the condition that deletion of any edge in this subgroup of interactions causes a significant change of the attractor landscape of the original network by changing its primary attractor. The robust neighbor is defined by the subgroup of interactions satisfying that deletion of any edge in the subgroup of interactions does not affect the attractor landscape of the original network much, by preserving its primary attractor. The evolvable core (robust neighbor) sub-network is defined by a sub-network composed of the evolvable core (robust neighbor, respectively) links and all the nodes of the original network. The evolvability (robustness) score of a node is defined by the proportion of evolvable core (robust neighbor, respectively) links connected to the node over all links associated with the node.

### Decomposition algorithm to identify the evolvable core and robust neighbor

We identified the evolvable core and the robust neighbor of a Boolean network through the following processes (see Fig. S17): (i) A Boolean network can be represented by a directed graph 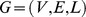, where *V* is a set of nodes, *E* is a set of edges, and *L* is a set of logic tables. Each edge can be represented by 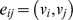 where *v_i_* is a start node and *v_j_* is an end node. The logic table of node *v_j_* can be represented by *l*(*v_j_*) and the reduced logic table of node *v_j_* when the state value of *v_i_* is *x* (0 or 1) can be represented by 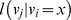. The logic table of each node is a set of output node states for each combination of input node states. (ii) We randomly sample 10,000 initial states which converge to the primary attractor in the original network. (iii) We then consider a copy, termed ‘Reduced Network’, of the original network. For each edge 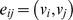 we remove the insignificant edges in which 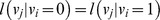, and update the Reduced Network with the edge-removed network. (iv) We then define a set of edges 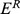 for the reduction which is empty initially. (v) For each edge 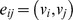 in the Reduced Network we test if the 10,000 initial states are attracted to the primary attractor in the Reduced Network with the selected edge removed. If the primary attractor is preserved, we add the selected edge to 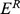. (vi) We then randomly select an edge 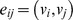 from 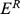 and test if the 10,000 initial states are attracted to the primary attractor in Reduced Network with the selected edge removed. If the primary attractor is preserved, we update the Reduced Network with the edge-removed network. (vii) We repeat the above process (vi) until the Reduced Network cannot be reduced any more. After all, the Reduced Network becomes the evolvable core and the sub-network obtained by subtracting the evolvable core links from the original network becomes the robust neighbor.

### Chemical inhibition and overexpression experiments

The experiments with chemical inhibitions and overexpression of HRas and HRas mutants were performed in HeLa cells (ATCC CCL-2), the overexpression of GFP-tagged kinases was performed in HEK293 (ATCC CRL-11268). ML7 (I2764) and blebbistatin (B0560) were purchased at Sigma. Antibodies against ppERK (M8159) and total ERK (M5670) were from Sigma. Antibodies against pJNK (9251), total JNK (9252), pP38 (9211), total P38 (9212), pAKT (9275) and total Akt (9272) were from Cell Signaling. The antibody against Ras (OP40) was from Calbiochem. All GFP-tagged kinase plasmids were generated through Gateway® cloning (Invitrogen), using the pDONR-223 gateway entry vectors from the ‘Human Kinase Open Reading Frame collection’ (Addgene 1000000014), and the destination vector 221 pCS-EGFP. As a control plasmid we used the same destination vector, inserted with EGFP (pDONR-EGFP as entry vector). HRas mutants in the pDCR vector were a kind gift from Dr. Pierro Crespo. In these experiments expression of the empty vector was used as a control.

For the chemical inhibitions, HeLa cells were treated with either 2 µM ML7 for MLCK inhibitions or 1 µM blebbistatin for myosin inhibition, 3 hours before lysis. For all overexpression experiments, cells were transfected 24 hours after seeding and 48 hours before lysis. In the experiments with chemical inhibitions and HRas mutant overexpression in HeLa cells, the cells were starved for 3 hours, and stimulated with 10% fetal bovine serum for 1 hour for JNK activation. Cells were starved for 16 hours, and stimulated with 10% fetal bovine serum for 10 minutes for ERK, Akt and P38 activation. All measurements upon overexpression of the GFP-tagged kinases in HEK293 were performed in growing conditions, 48 hours after transfection.

All cells were lysed in 20 mM HEPES, 150 mM NaCl, 1% NP40. SDS-PAGE was performed, followed by Western blotting using the antibodies against pJNK, pP38, total ERK or pAkt or pan-Ras. The membranes were then stripped with 1%SDS, 0.2 M glycin at pH 2.5, re-blocked in 4%BSA in TBS-T and incubated with antibodies against total JNK, total P38, ppERK or total AKT. We chose our outputs according to the following criteria, i.e. that (i) they are linked to nodes in the evolvable core and robust neighbor enabling a comparative assessment of perturbation experiments; and (ii) they are experimentally tractable. This is how we selected to measure ERK, Akt, p38, and JNK activation. In addition, all of the outputs are linked to the primary attractor: ERK activation is linked closely to Raf (which is the main upstream activator of ERK) as well as PKC; Akt activation is closely linked to phosphatidylinositol signaling; and the stress activated MAP kinases JNK and p38 are closely linked to PKC and also phosphatidylinositol signaling.

### Degree heterogeneity and characteristic path length

The degree heterogeneity was defined by the variance of the degree distribution divided by the average of the degree distribution [31]. The characteristic path length was defined by the average of the shortest path lengths over all pairs of nodes [32].

### The evolutionary rate and species broadness

The evolutionary rates were defined by the ratios of the non-synonymous substitution rates (dN) and the synonymous substitution rates (dS) for homologous gene pairs in human and mouse, and we obtained the evolutionary rates of 13815 genes from the Human PAML Browser [57]. We defined the species broadness of a gene as the number of species in which homologs of the gene exist. The homolog information of 20 species was extracted from the HomoloGene database [58] in the NCBI and the species broadness of 19571 genes was obtained from the database. In order to investigate the correlation between the evolutionary rates or species broadness and the evolvability score, we mapped each node of the network into the corresponding genes based on EntrezGene IDs (see Table S4). Some nodes such as PIP_4 do not have corresponding EntrezGene ID, some nodes such as MKK3 correspond to one EntrezGene ID, and some nodes such as MLCP correspond to multiple EntrezGene ID. Based on the transformation, we obtained 631 genes which have EntrezGene ID. Among 631 genes, 549 genes have evolutionary rate values and 629 genes have species broadness values.

### Immune system, oncogene, FDA-approved drug target, experimental drug target, receptor, and kinase

The list of genes related to immune system was selected as the genes classified into the gene ontology (GO) term ‘immune system process (GO:0002376)’ [59]. This list contains 944 genes related to immune system, 109 of which are included in the human signaling network (Table S20). The list of oncogenes was obtained from the OMIM database [60] in the NCBI. This list contains 51 oncogenes, 12 of which are included in the human signaling network (Table S21). The drug target list was obtained from the DrugBank database [61]. This list contains 1330 FDA-approved drug targets, 168 of which are included in the human signaling network (Table S22) and 765 experimental drug targets, 52 of which are included in the human signaling network (Table S23). The list of genes related to receptor or kinase was obtained on the basis of GO terms, ‘receptor activity (GO:0004872)’ or ‘kinase activity (GO:0016301). This list contains 1688 genes related to receptors, 177 of which are included in the human signaling network (Table S24) and 770 genes related to kinases, 107 of which are included in the human signaling network (Table S25).

### Normalized average evolvability (robustness) score

In order to calculate the normalized average evolvability or robustness score, we mapped each node of the network into the corresponding genes based on EntrezGene IDs and calculated the average of the proportions of evolvable core (or robust neighbor) links of the resulting 631 genes. The normalized average evolvability (robustness) score is defined as the average of the proportions of evolvable core (robust neighbor, respectively) links for a particular gene group (genes related to immune system, oncogenes, FDA-approved drug targets, experimental drug targets, receptors, or kinases) divided by the average of the proportions of evolvable core (robust neighbor, respectively) links for the total 631 genes.

### Statistical analysis

We performed one-sided two sample t-test to compare the number of feedback loops (Figs. 3A–C), degree heterogeneity (Fig. 3D), and the characteristic path length (Fig. 3G) for the evolvable core and random-deletion sub-networks; the number of connected components (Fig. 3H) and characteristic path length (Fig. 3I) for the robust neighbor and random-selection sub-networks; the average perturbation effect (Fig. 2C). We performed Pearson's correlation test to analyze the significance of the correlation between the evolutionary rates (Fig. 4A) or species broadness (Fig. 4B) and the evolvability score. In order to compare the normalized average evolvability (robustness) score of a particular gene group (the genes related to the immune system process, oncogenes, FDA-approved drug targets, experimental drug targets, receptors, or kinases) with that of the random control group, the permutation test with 100,000 permutations was performed. The random control group was obtained by randomly selecting genes out of 631 genes where the sample size was fixed as the size of the given particular gene group.

### Availability of the software

We have implemented the proposed decomposition algorithm as software. It is available from the http://sbie.kaist.ac.kr/software and as part of the Supplementary Materials.

## Supporting Information

Figure S1 **The histogram of the estimated basin sizes for the major twenty attractors, computed using various numbers of randomly selected initial states** (10,000 for A, 100,000 for B, and 1,000,000 for C).
(TIF)

Figure S2 **Subnetwork of the human signaling network composed of 16 nodes which are ‘ON’ at least once in their cyclic state transitions of the primary attractor.** The subnetwork composed of these 16 nodes and their interactions consists of three separate modules: a module for phosphatidylinositol signaling (red nodes), a module for Raf activation (blue nodes), and a module for PKC activation (green node). The ‘ON’ nodes in the primary attractor are related to precursors of second messengers or inactive forms of kinases. In other words, the primary attractor can be considered as a ‘ready’ state of the signaling network, which might be the nominal condition of cell signaling.
(TIF)

Figure S3 **Number of robust neighbor links with respect to different random seeds of initial states (A) and deletion order (B).** This figure shows that the number of robust neighbor links is similar irrespective of different random seeds.
(TIF)

Figure S4 **Representative western blots and quantification of the MAPK (ERK, JNK, p38) and Akt output activities upon chemical inhibitions (A) and overexpression of HRas mutants (B) and GFP-tagged kinases (C).**
(TIF)

Figure S5 **Number of self-loops, two-nodes feedbacks, and three-nodes feedbacks in the evolvable cores with respect to different random seeds of initial states (A, C, and E) and deletion order (B, D, and F).** This figure shows that the evolvable cores obtained from the proposed identification algorithm include, irrespective of different random seeds, a significantly large number of feedbacks compared to the random-deletion networks. Error bars on the white bars outlined in red denote the standard errors of the average values.
(TIF)

Figure S6 **The network heterogeneity of evolvable cores, ratio of robust neighbor links, and characteristic path lengths of evolvable cores with respect to different random seeds of initial states (A, C, and E) and deletion order (B, D, and F).** This figure shows that the network heterogeneity (A and B) and characteristic path lengths (E and F) of the evolvable cores obtained from the proposed identification algorithm are, irrespective of different random seeds, significantly higher than those of random-deletion networks, and that middle-degree nodes (red bars of C and D) contain more robust neighbor links than low-degree nodes (blue bars of C and D) or high-degree nodes (green bars of C and D) for all different random seeds. Error bars of the white bars outlined in red in panels A, B, E, and F denote the standard errors of the average values.
(TIF)

Figure S7 **Number of connected components and the characteristic path lengths of robust neighbors with respect to different random seeds of initial states (A and C) and deletion order (B and D).** This figure shows that the number of connected components and the characteristic path lengths of the robust neighbors obtained from the proposed identification algorithm are, irrespective of different random seeds, smaller than those of random-selection networks.
(TIF)

Figure S8 **Relationship between two genetic properties (evolutionary rate and species broadness) and the evolvability score for each node with respect to different random seeds of initial states (A and C) and deletion order (B and D).** This figure shows that evolutionary rate (species broadness) is positively (negatively) correlated with the proportion of evolvable core links, irrespective of different random seeds.
(TIF)

Figure S9 **The normalized average evolvability and robustness scores of the genes related to immune system and those of the genes related to oncogene with respect to different random seeds of initial states (A and C) and deletion order (B and D).** This figure shows that the normalized average evolvability (robustness) score for the genes related to immune system (oncogene, respectively) is, irrespective of different random seeds, higher than that of the random control group.
(TIF)

Figure S10 **The normalized average evolvability and robustness scores of the FDA-approved drug targets which are not included in the experimental drug targets and those of the experimental drug targets which are not included in the FDA-approved drug targets.**
(TIF)

Figure S11 **The normalized average evolvability and robustness scores of FDA-approved drug targets, experimental drug targets, receptors, and kinases with respect to different random seeds of initial states (A, C, E, and G) and deletion order (B, D, F, and H).** This figure shows that the normalized average evolvability (robustness) score of the FDA-approved drug targets or receptors (experimental drug targets or kinases, respectively), irrespective of different random seeds, are higher than that of the random control group.
(TIF)

Figure S12 **Comparison of robust neighbor and redundant links in the context of canalizing function.** (A) Illustration of the canalizing function and non-canalizing function. (B) Venn diagram of the subgroups of links (evolvable core links and robust neighbor links) and redundant links in the context of canalizing function for the human signaling network.
(TIF)

Figure S13 **The number of evolvable core links and that of robust neighbor links under various input conditions.** (A) The number of evolvable core links under various input conditions, the number of links included in the intersection with the original evolvable core under zero input condition, and the number of links in the control. (B) The number of robust neighbor links under various input conditions, the number of links included in the intersection with the original robust neighbor under zero input condition, and the number of links in the control. Here, the control (random intersection) is the intersection between the random subgroup of links of the same size with the original evolvable core (or the original robust neighbor) and another random subgroup of links of the same size with the newly obtained evolvable core (or the newly obtained robust neighbor) under various input conditions. In this comparison, we used the first seed for random deletion order and random initial states as shown in Figure S3 for the original evolvable core and robust neighbor under zero input condition. *P*-values were obtained from one-sided two sample Chi square tests for 2 (the number of evolvable core links or robust neighbor links under zero input condition)×2 (the number of evolvable core links or robust neighbor links obtained from the simulations with one of the state values of nine ligands or the set of ligands for GPCRs (α_q_lig, α_i_lig, α_s_lig, and α_12_13_lig) set to ‘ON’ alternatively) contingency tables.
(TIF)

Figure S14 **The number of evolvable core links and that of robust neighbor links for asynchronous update.** (A) The number of evolvable core links for asynchronous update, the number of links included in the intersection with the original evolvable core for synchronous update, and the number of links in the control. (B) The number of robust neighbor links for asynchronous update, the number of links included in the intersection with the original robust neighbor for synchronous update, and the number of links in the control. Here, the control (random intersection) is the intersection between the random subgroup of links of the same size with the original evolvable core (or the original robust neighbor) and another random subgroup of links of the same size with the newly obtained evolvable core (or the newly obtained robust neighbor) for asynchronous update. In this comparison, we used the first seed for random deletion order and random initial states as shown in Figure S3 for the original evolvable core and robust neighbor for synchronous update. *P*-values were obtained from one-sided two sample Chi square tests for 2 (the number of evolvable core links or robust neighbor links for synchronous update)×2 (the number of evolvable core links or robust neighbor links for asynchronous update) contingency tables.
(TIF)

Figure S15 **The emergent function of information processing in the evolvable core of the human signaling network.** We followed the same procedure proposed by Helikar *et al.* [22]. These scatter plots show how the network system clusters combinations of 10,000 random inputs and then maps them to the global outputs. The input values (0∼100%) of three input nodes (‘EGF’, ‘ECM’, and ‘ExtPump’) associated with the 15 most common outputs of Helikar *et al.* [22] are plotted in 3-dimensional space using principle component analysis, where the input value denotes a percentage ‘ON’ for the input node in the Boolean iteration of 1,000 times and the output value denotes the average number of ‘ON’s over the last 100 iterations out of the Boolean iteration of 1,000 times. The output values were categorized by using three different ranges: 0 (0∼9%), 1 (10∼29%), and 2 (30∼100%). A four-tuple of numbers in the legends represents a category of the four output nodes (‘Akt’, ‘Erk’, ‘Rac’, and ‘Cdc42’).
(TIF)

Figure S16 **Decomposition of the JAK/STAT signaling network.** (A) JAK/STAT signaling network. (B) Evolvable core. (C) Robust neighbor. (D) The correlation between evolutionary rate and evolvability score. (E) The correlation between species broadness and evolvability score. (F) The normalized average evolvability and robustness scores of the genes related to immune system. (G) The normalized average evolvability and robustness scores of the oncogenes.
(TIF)

Figure S17 **Decomposition of the Keratinocyte signaling network.** (A) Keratinocyte signaling network. (B) Evolvable core. (C) Robust neighbor. (D) The correlation between evolutionary rate and evolvability score. (E) The correlation between species broadness and evolvability score. (F) The normalized average evolvability and robustness scores of the genes related to immune system. (G) The normalized average evolvability and robustness scores of the oncogenes.
(TIF)

Figure S18 **A flow diagram illustrating the proposed algorithm for identifying the evolvable core and robust neighbor.**
(TIF)

Table S1 **Primary attractor states of the human signaling network for the first seed of initial states as shown in Figure S3.**
(DOC)

Table S2 **List of links in the evolvable core for the first seed of deletion order and the first seed of initial states as shown in Figure S3.**
(DOC)

Table S3 **List of links in the robust neighbor for the first seed of deletion order and the first seed of initial states as shown in Figure S3.**
(DOC)

Table S4 **The name, EnetrezGene IDs, degree, evolvability score, and robustness score for each node of the network.**
(DOC)

Table S5 **The standard deviation of the evolvability scores of the targets of multi-target drug.**
(DOC)

Table S6
**The normalized proportion of the paths in the evolvable core from perturbed nodes to output nodes over such paths in the original network.** The normalized proportion (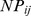) of the paths in the evolvable core from a node *i* to a node *j* is defined as follows: 
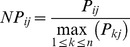
, where 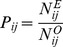, 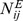 denotes the number of paths in the evolvable core from a node *i* to a node *j*, 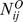 denotes the number of paths in the original network from a node *i* to a node *j*, and *n* denotes the number of all the nodes in the original network.(DOC)

Table S7 **List of evolvable core links, robust neighbor links, and redundant links in the context of canalizing function.**
(DOC)

Table S8 **Primary attractor states of the human signaling network obtained from the simulation with the state value of the input node ‘EGF’ set to ‘ON’.**
(DOC)

Table S9 **Primary attractor states of the human signaling network obtained from the simulation with the state value of the input node ‘ECM’ set to ‘ON’.**
(DOC)

Table S10 **Primary attractor states of the human signaling network obtained from the simulation with the state value of the input node ‘α_q_lig’ set to ‘ON’.**
(DOC)

Table S11 **Primary attractor states of the human signaling network obtained from the simulation with the state value of the input node ‘α_i_lig’ set to ‘ON’.**
(DOC)

Table S12 **Primary attractor states of the human signaling network obtained from the simulation with the state value of the input node ‘α_s_lig’ set to ‘ON’.**
(DOC)

Table S13 **Primary attractor states of the human signaling network obtained from the simulation with the state value of the input node ‘α_12_13_lig’ set to ‘ON’.**
(DOC)

Table S14 **Primary attractor states of the human signaling network obtained from the simulation with the state value of the input node ‘Stress’ set to ‘ON’.**
(DOC)

Table S15 **Primary attractor states of the human signaling network obtained from the simulation with the state value of the input node ‘IL1_TNF’ set to ‘ON’.**
(DOC)

Table S16 **Primary attractor states of the human signaling network obtained from the simulation with the state value of the input node ‘ExtPump’ set to ‘ON’.**
(DOC)

Table S17 **Primary attractor states of the human signaling network obtained from the simulation with the state values of the four ligands (α_q_lig, α_i_lig, α_s_lig, and α_12_13_lig) for GPCRs set to ‘ON’.**
(DOC)

Table S18 **Primary attractor states of the human signaling network obtained from the simulation with asynchronous update of Boolean functions.**
(DOC)

Table S19 **The emergent function of information processing in the evolvable core of the human signaling network.** We followed the same procedure proposed by Helikar *et al.* [22]. This table shows that the relatively small number of output categories are observed from 10,000 simulations with different inputs, where the input values denotes a percentage ‘ON’ for the input node in the Boolean iteration of 1,000 times and the output values denote the average number of ‘ON’s over the last 100 iterations out of the Boolean iteration of 1,000 times. The output values were categorized by using three different ranges; 0 (0∼9%), 1 (10∼29%), and 2 (30∼100%). A four-tuple of numbers in the legends represents a category of four output nodes (‘Akt’, ‘Erk’, ‘Rac’, and ‘Cdc42’).
(DOC)

Table S20 **The list of genes related to immune system that are included in the human signaling network.**
(DOC)

Table S21 **The list of oncogenes that are included in the human signaling network.**
(DOC)

Table S22 **The list of FDA-approved drug targets that are included in the human signaling network.**
(DOC)

Table S23 **The list of experimental drug targets that are included in the human signaling network.**
(DOC)

Table S24 **The list of genes related to receptors that are included in the human signaling network.**
(DOC)

Table S25 **The list of genes related to kinases that are included in the human signaling network.**
(DOC)
