# Supplementary figures and images for: Robustness and Evolvability of the Human Signaling Network
Source: PLoS Comput Biol. 2014 Jul 31;10(7):e1003763. doi: 10.1371/journal.pcbi.1003763 (PMC4117429; doi:10.1371/journal.pcbi.1003763)

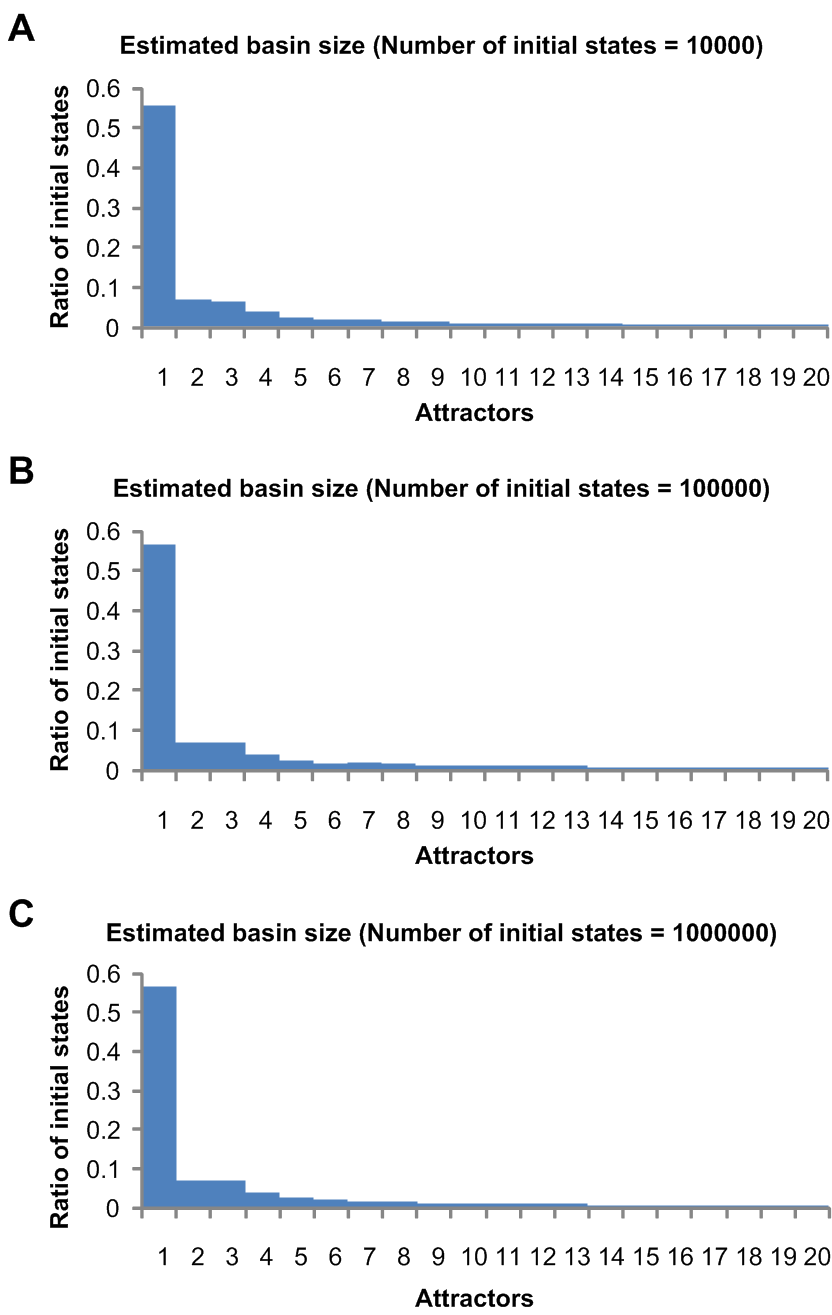

Supplement: Figure S1 — The histogram of the estimated basin sizes for the major twenty attractors, computed using various numbers of randomly selected initial states (10,000 for A, 100,000 for B, and 1,000,000 for C). (TIF) [file pcbi.1003763.s001.tif]

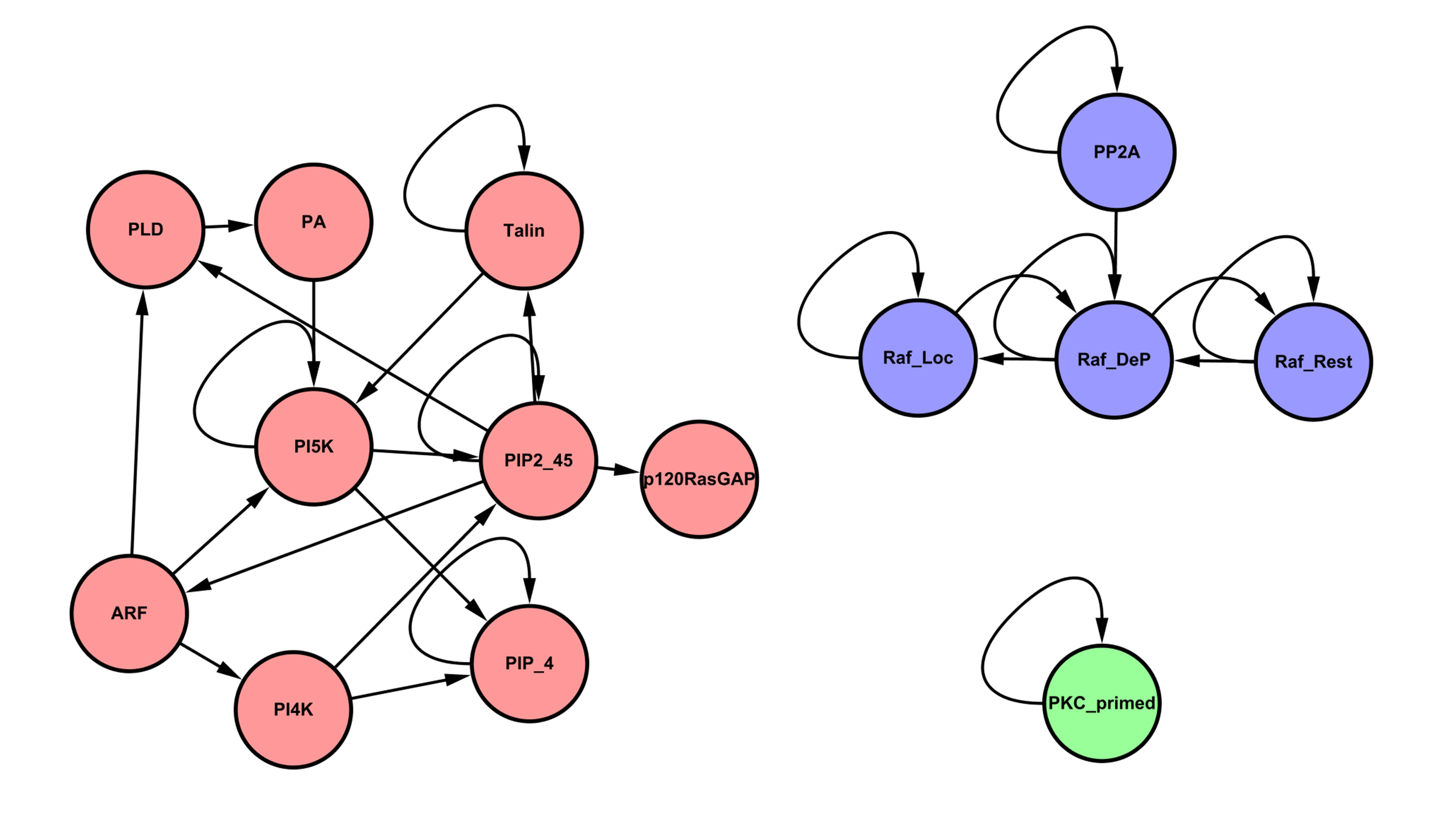

Supplement: Figure S2 — Subnetwork of the human signaling network composed of 16 nodes which are ‘ON’ at least once in their cyclic state transitions of the primary attractor. The subnetwork composed of these 16 nodes and their interactions consists of three separate modules: a module for phosphatidylinositol signaling (red nodes), a module for Raf activation (blue nodes), and a module for PKC activation (green node). The ‘ON’ nodes in the primary attractor are related to precursors of second messengers or inactive forms of kinases. In other words, the primary attractor can be considered as a ‘ready’ state of the signaling network, which might be the nominal condition of cell signaling. (TIF) [file pcbi.1003763.s002.tif]

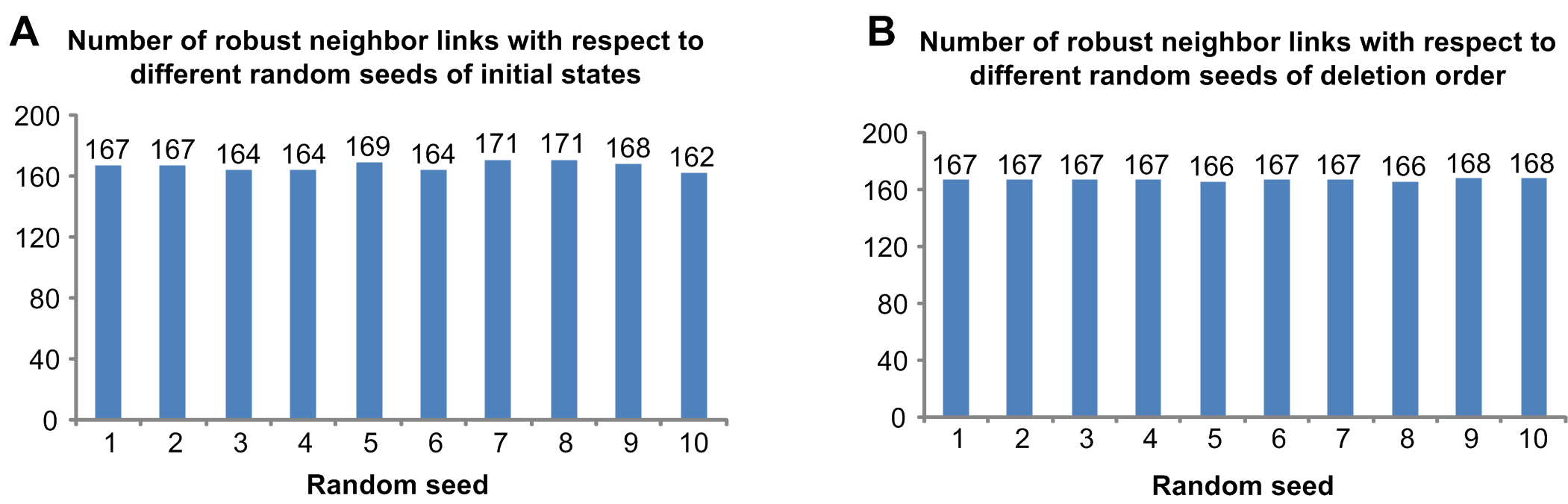

Supplement: Figure S3 — Number of robust neighbor links with respect to different random seeds of initial states (A) and deletion order (B). This figure shows that the number of robust neighbor links is similar irrespective of different random seeds. (TIF) [file pcbi.1003763.s003.tif]

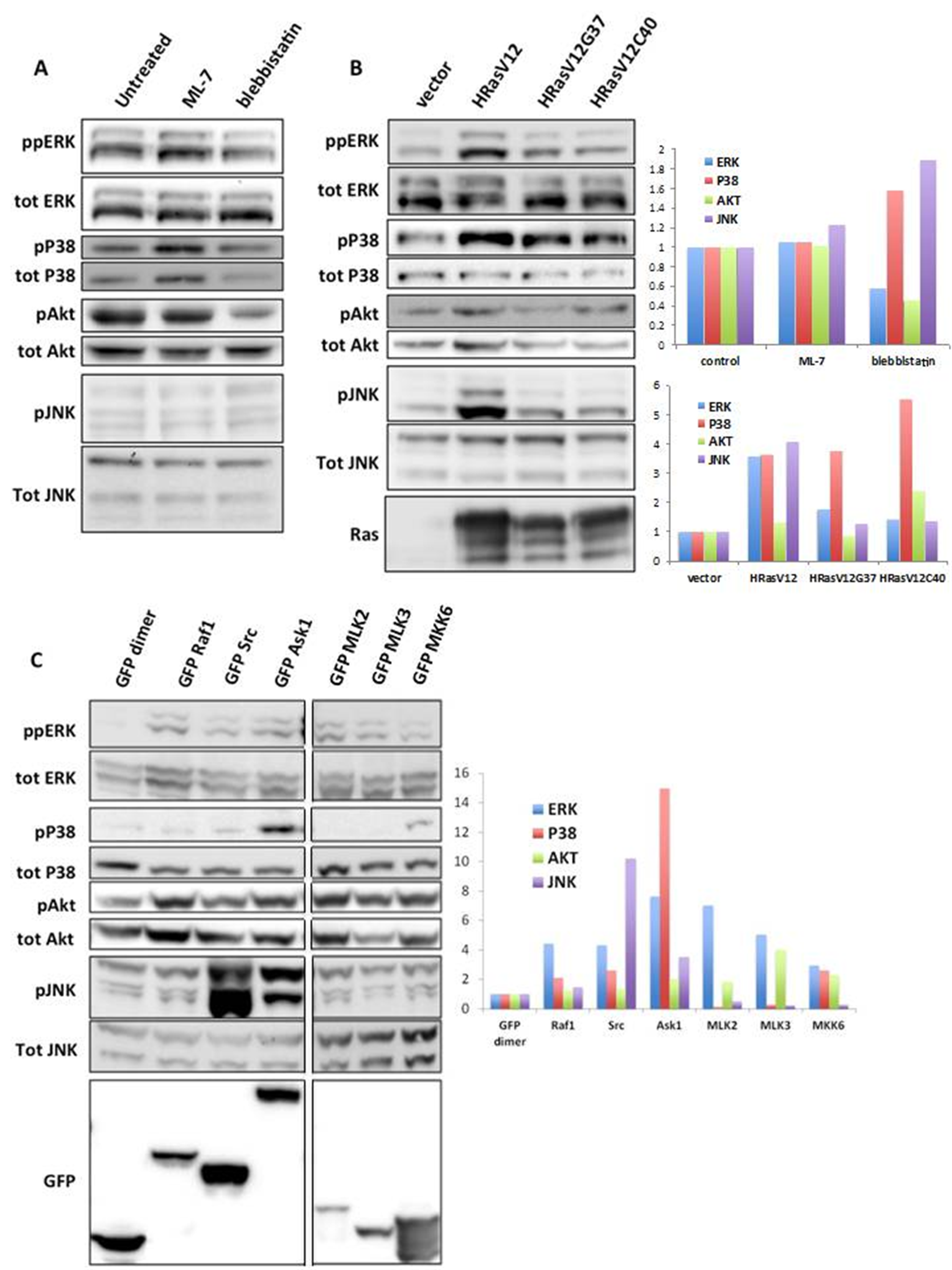

Supplement: Figure S4 — Representative western blots and quantification of the MAPK (ERK, JNK, p38) and Akt output activities upon chemical inhibitions (A) and overexpression of HRas mutants (B) and GFP-tagged kinases (C). (TIF) [file pcbi.1003763.s004.tif]

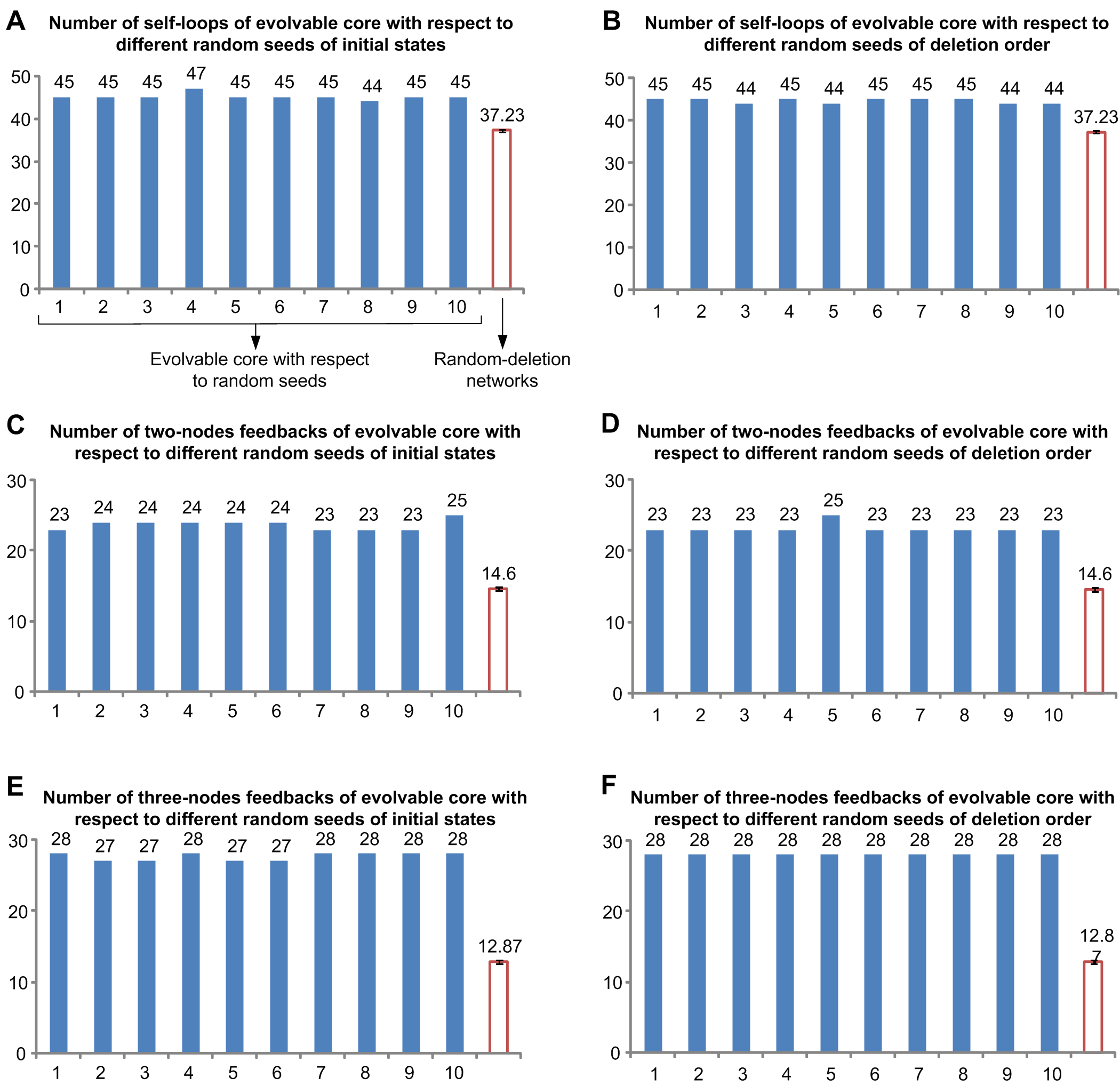

Supplement: Figure S5 — Number of self-loops, two-nodes feedbacks, and three-nodes feedbacks in the evolvable cores with respect to different random seeds of initial states (A, C, and E) and deletion order (B, D, and F). This figure shows that the evolvable cores obtained from the proposed identification algorithm include, irrespective of different random seeds, a significantly large number of feedbacks compared to the random-deletion networks. Error bars on the white bars outlined in red denote the standard errors of the average values. (TIF) [file pcbi.1003763.s005.tif]

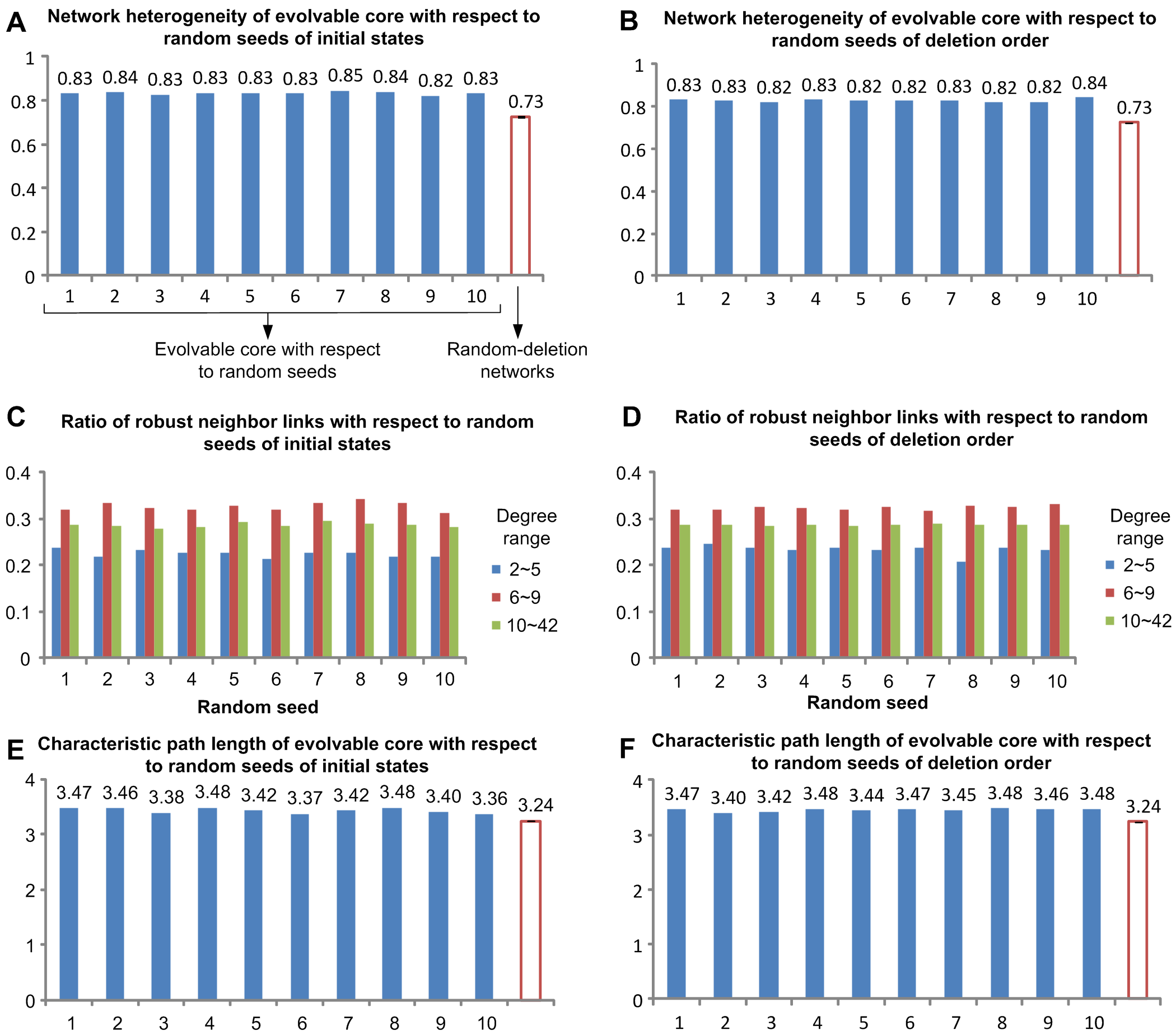

Supplement: Figure S6 — The network heterogeneity of evolvable cores, ratio of robust neighbor links, and characteristic path lengths of evolvable cores with respect to different random seeds of initial states (A, C, and E) and deletion order (B, D, and F). This figure shows that the network heterogeneity (A and B) and characteristic path lengths (E and F) of the evolvable cores obtained from the proposed identification algorithm are, irrespective of different random seeds, significantly higher than those of random-deletion networks, and that middle-degree nodes (red bars of C and D) contain more robust neighbor links than low-degree nodes (blue bars of C and D) or high-degree nodes (green bars of C and D) for all different random seeds. Error bars of the white bars outlined in red in panels A, B, E, and F denote the standard errors of the average values. (TIF) [file pcbi.1003763.s006.tif]

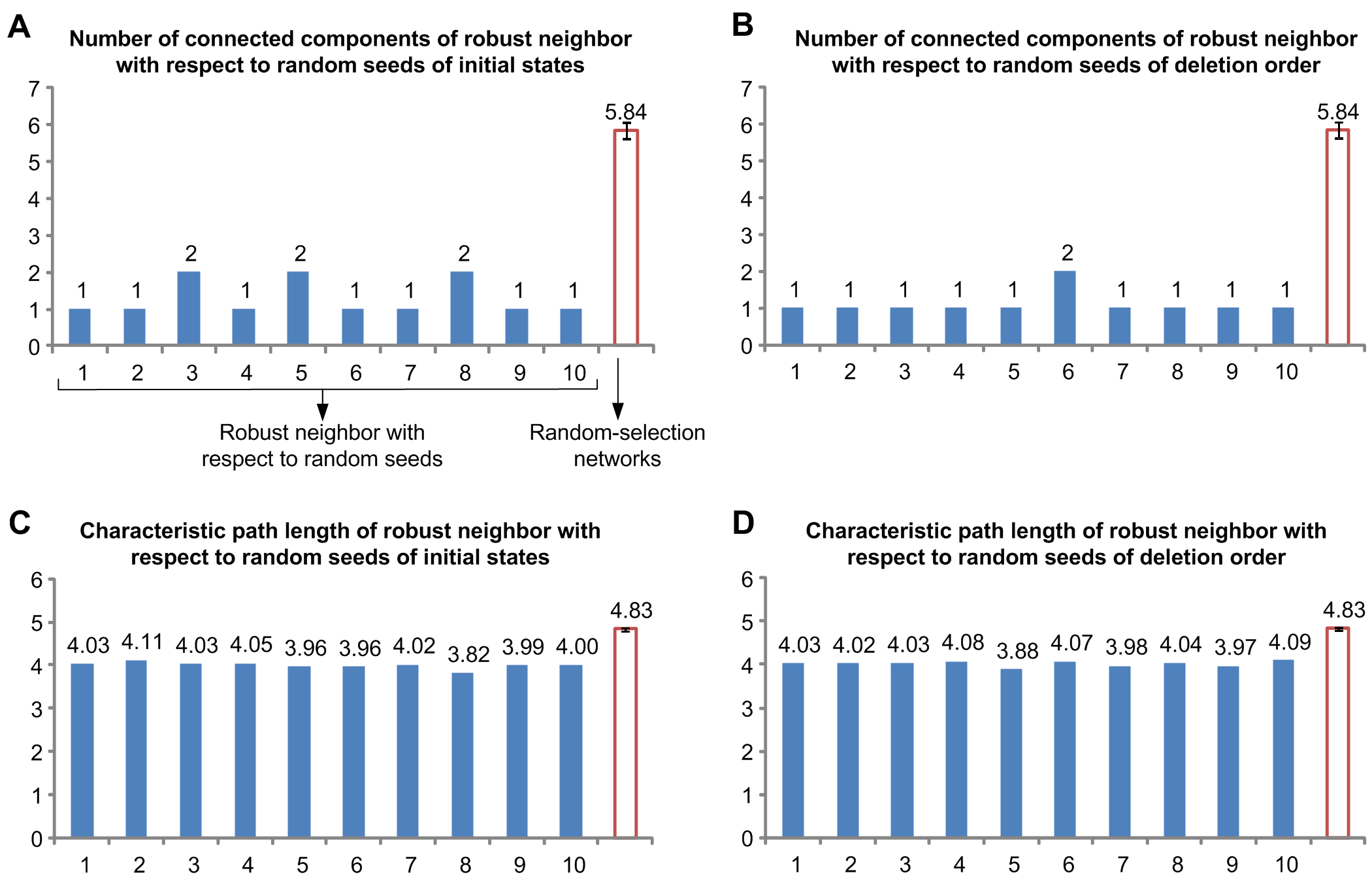

Supplement: Figure S7 — Number of connected components and the characteristic path lengths of robust neighbors with respect to different random seeds of initial states (A and C) and deletion order (B and D). This figure shows that the number of connected components and the characteristic path lengths of the robust neighbors obtained from the proposed identification algorithm are, irrespective of different random seeds, smaller than those of random-selection networks. (TIF) [file pcbi.1003763.s007.tif]

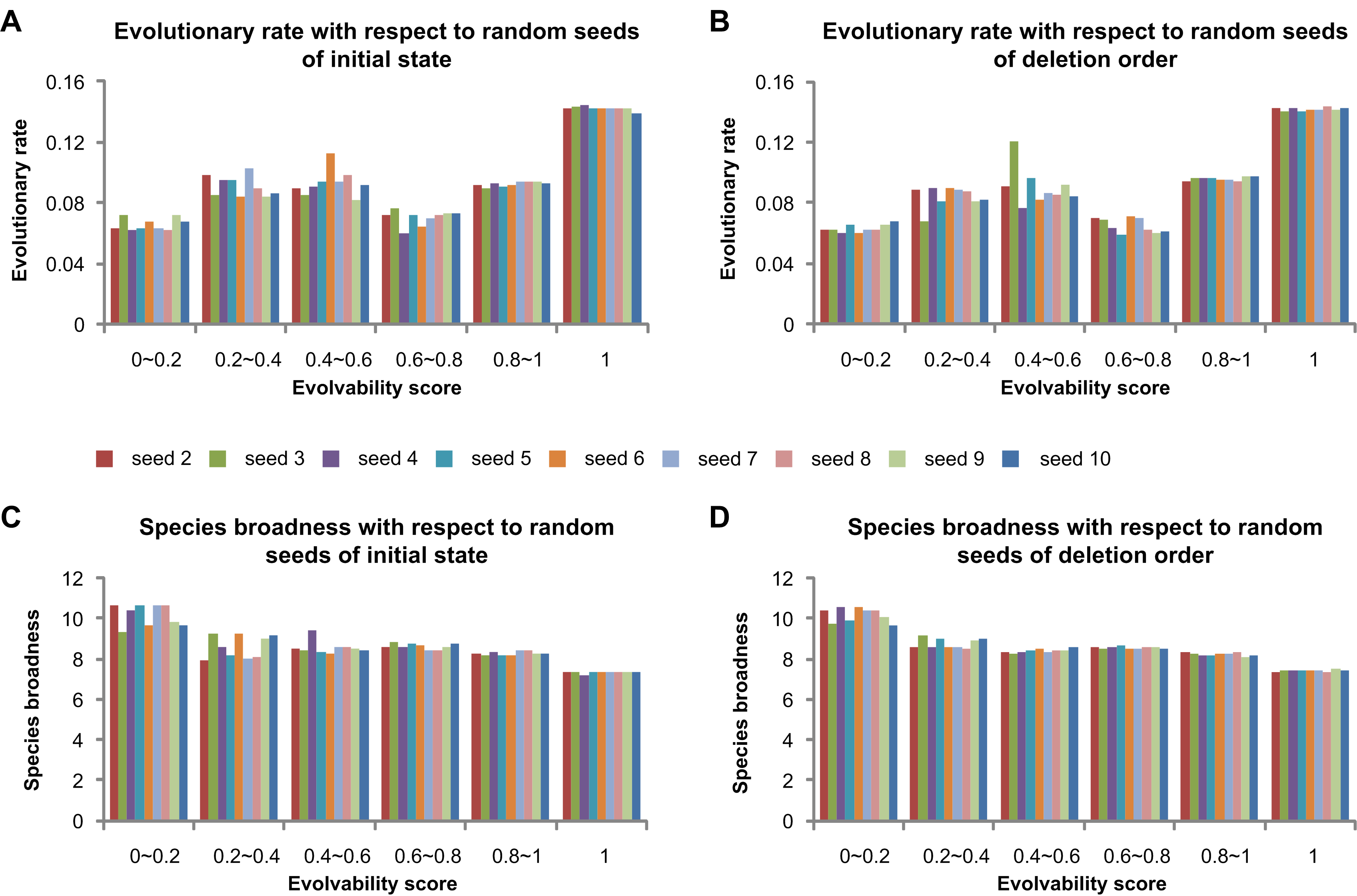

Supplement: Figure S8 — Relationship between two genetic properties (evolutionary rate and species broadness) and the evolvability score for each node with respect to different random seeds of initial states (A and C) and deletion order (B and D). This figure shows that evolutionary rate (species broadness) is positively (negatively) correlated with the proportion of evolvable core links, irrespective of different random seeds. (TIF) [file pcbi.1003763.s008.tif]

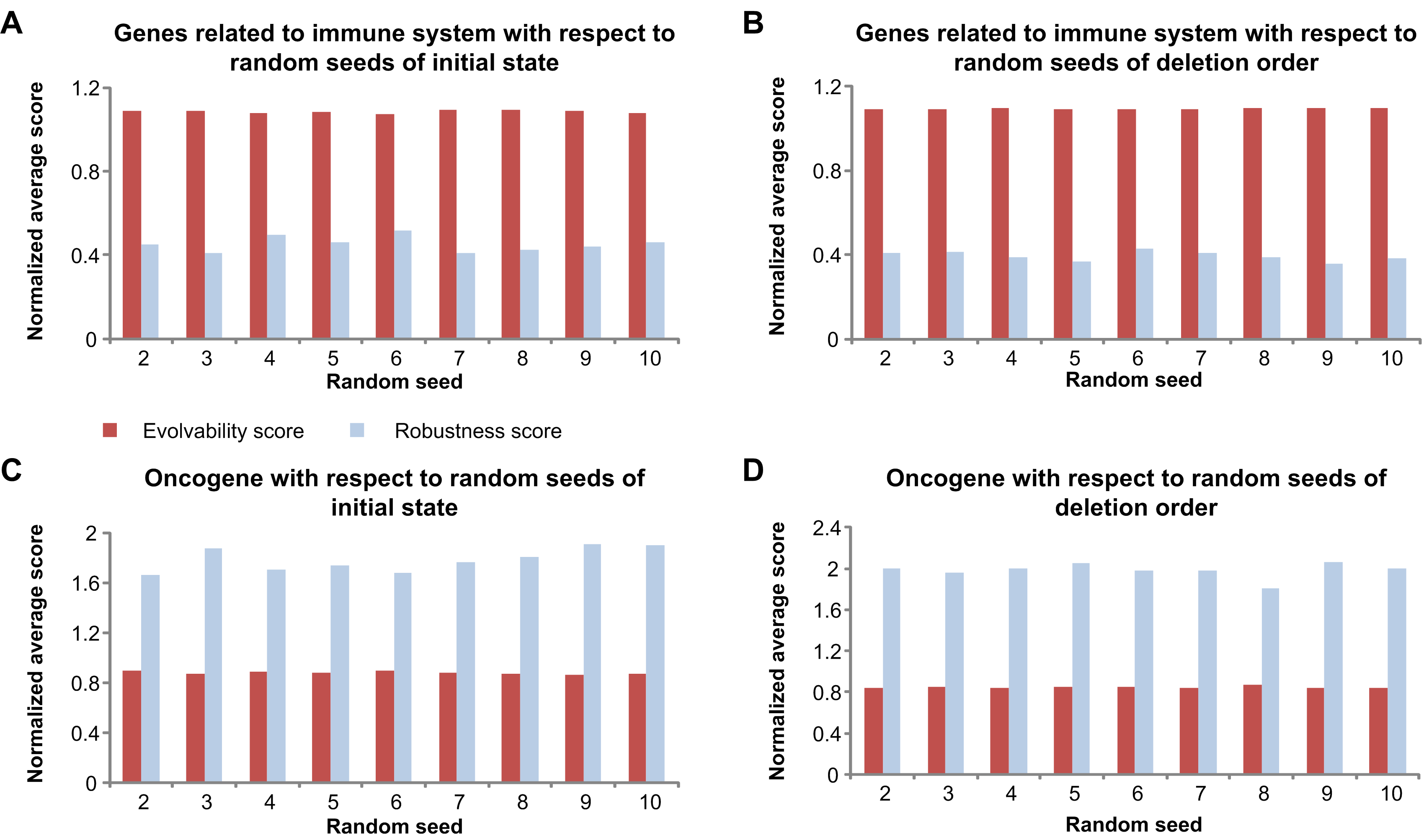

Supplement: Figure S9 — The normalized average evolvability and robustness scores of the genes related to immune system and those of the genes related to oncogene with respect to different random seeds of initial states (A and C) and deletion order (B and D). This figure shows that the normalized average evolvability (robustness) score for the genes related to immune system (oncogene, respectively) is, irrespective of different random seeds, higher than that of the random control group. (TIF) [file pcbi.1003763.s009.tif]

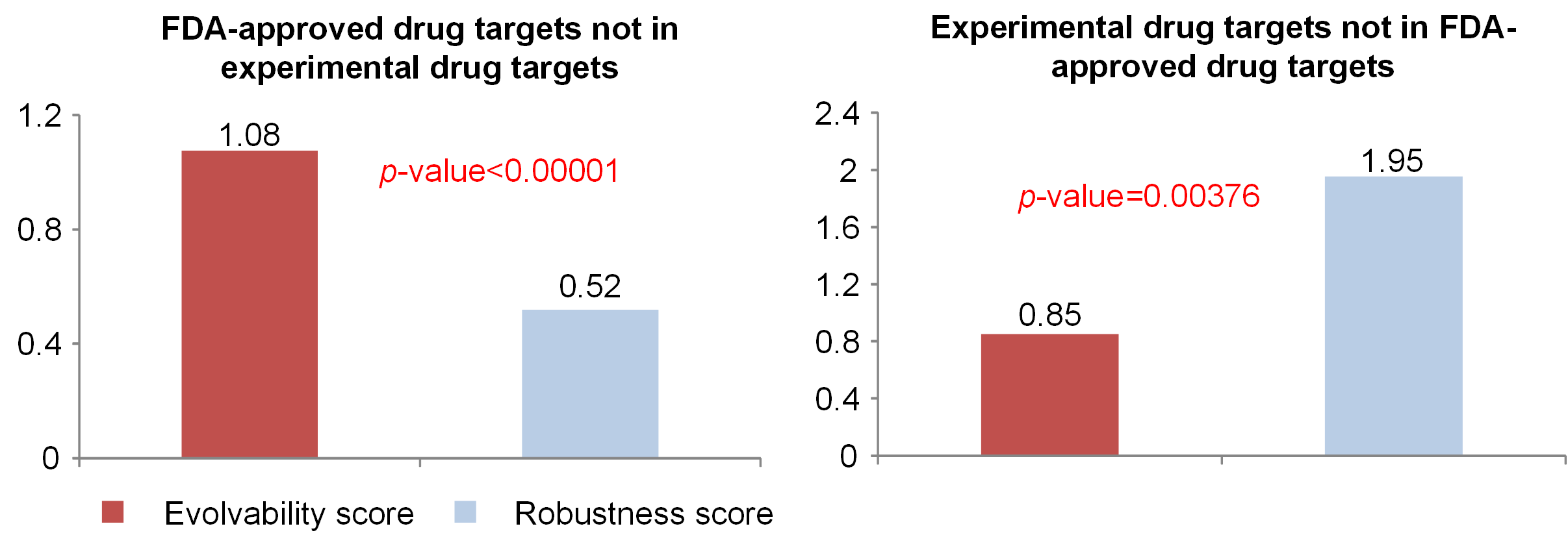

Supplement: Figure S10 — The normalized average evolvability and robustness scores of the FDA-approved drug targets which are not included in the experimental drug targets and those of the experimental drug targets which are not included in the FDA-approved drug targets. (TIF) [file pcbi.1003763.s010.tif]

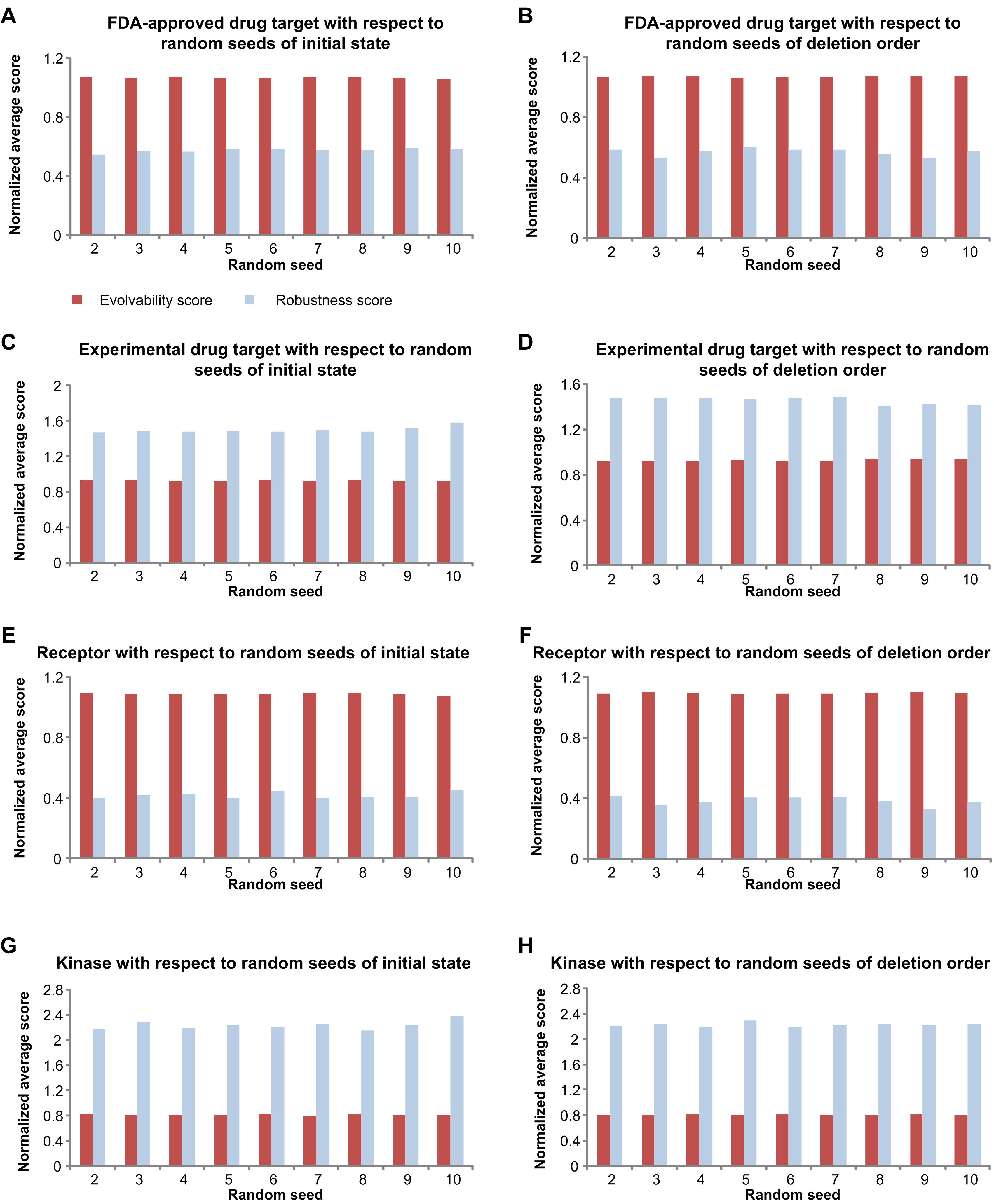

Supplement: Figure S11 — The normalized average evolvability and robustness scores of FDA-approved drug targets, experimental drug targets, receptors, and kinases with respect to different random seeds of initial states (A, C, E, and G) and deletion order (B, D, F, and H). This figure shows that the normalized average evolvability (robustness) score of the FDA-approved drug targets or receptors (experimental drug targets or kinases, respectively), irrespective of different random seeds, are higher than that of the random control group. (TIF) [file pcbi.1003763.s011.tif]

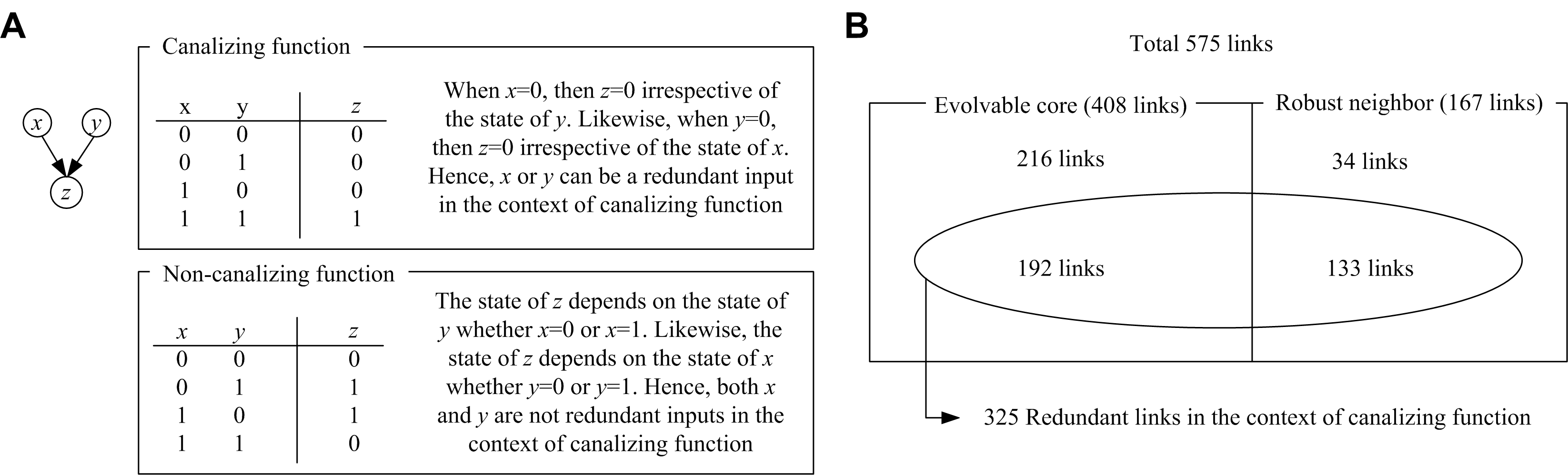

Supplement: Figure S12 — Comparison of robust neighbor and redundant links in the context of canalizing function. (A) Illustration of the canalizing function and non-canalizing function. (B) Venn diagram of the subgroups of links (evolvable core links and robust neighbor links) and redundant links in the context of canalizing function for the human signaling network. (TIF) [file pcbi.1003763.s012.tif]

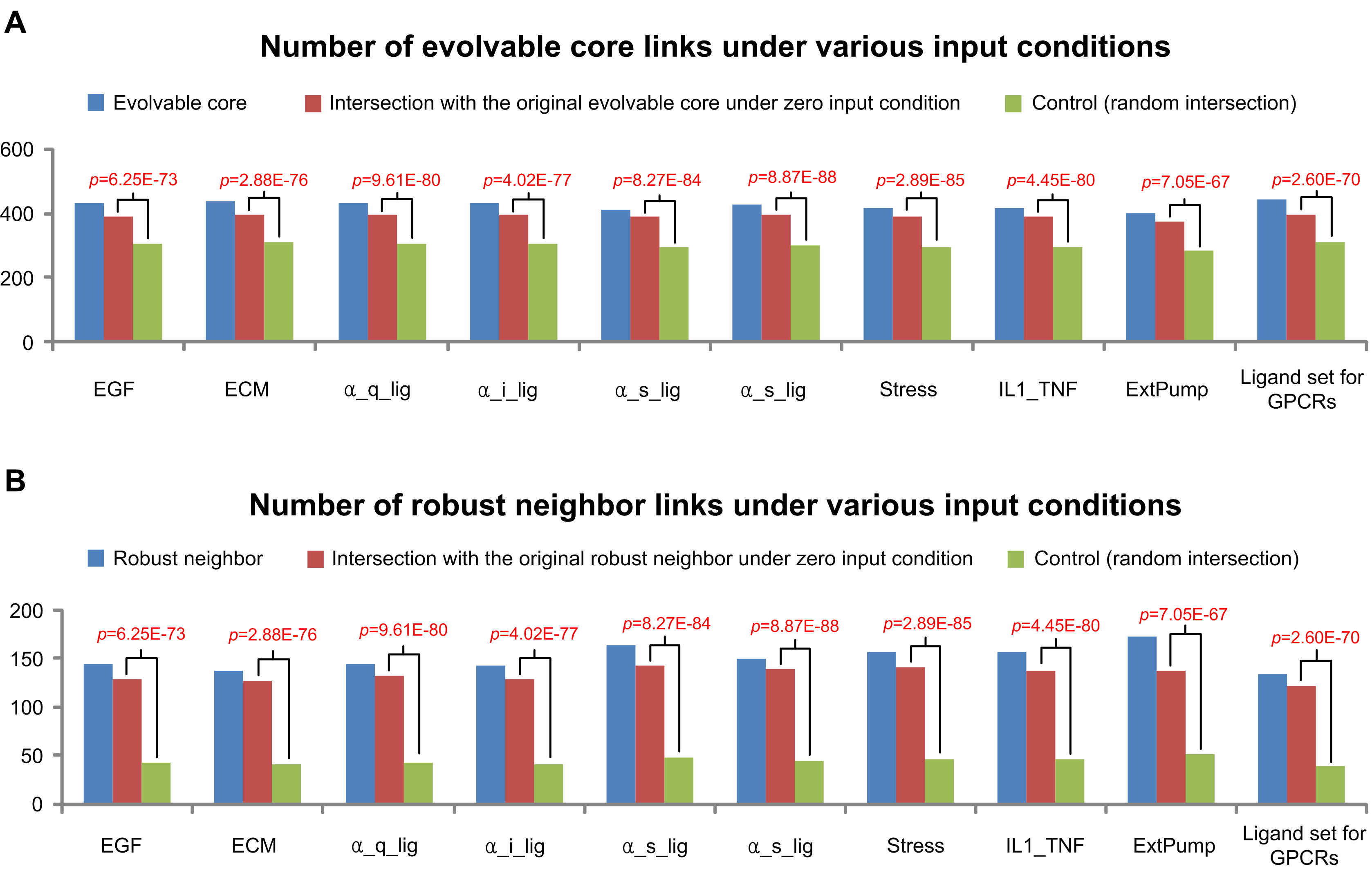

Supplement: Figure S13 — The number of evolvable core links and that of robust neighbor links under various input conditions. (A) The number of evolvable core links under various input conditions, the number of links included in the intersection with the original evolvable core under zero input condition, and the number of links in the control. (B) The number of robust neighbor links under various input conditions, the number of links included in the intersection with the original robust neighbor under zero input condition, and the number of links in the control. Here, the control (random intersection) is the intersection between the random subgroup of links of the same size with the original evolvable core (or the original robust neighbor) and another random subgroup of links of the same size with the newly obtained evolvable core (or the newly obtained robust neighbor) under various input conditions. In this comparison, we used the first seed for random deletion order and random initial states as shown in Figure S3 for the original evolvable core and robust neighbor under zero input condition. P-values were obtained from one-sided two sample Chi square tests for 2 (the number of evolvable core links or robust neighbor links under zero input condition)×2 (the number of evolvable core links or robust neighbor links obtained from the simulations with one of the state values of nine ligands or the set of ligands for GPCRs (α_q_lig, α_i_lig, α_s_lig, and α_12_13_lig) set to ‘ON’ alternatively) contingency tables. (TIF) [file pcbi.1003763.s013.tif]

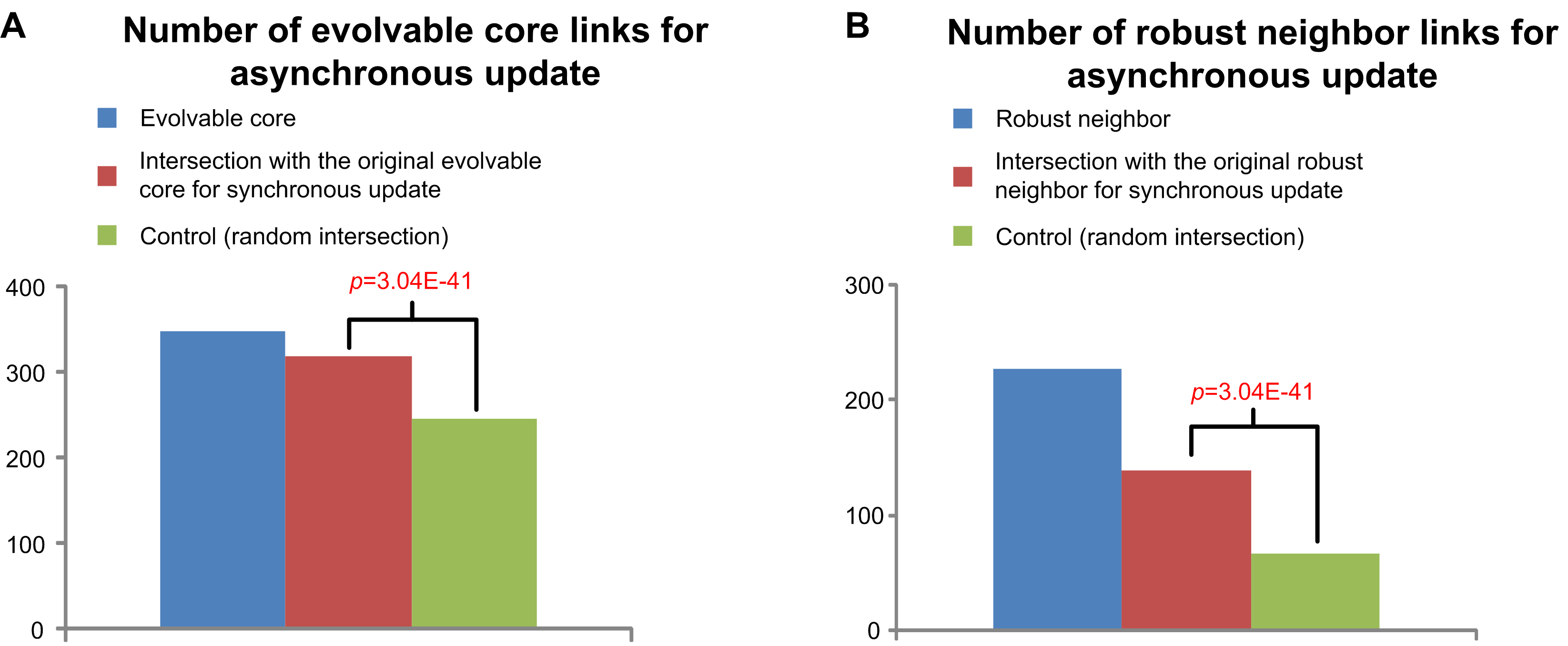

Supplement: Figure S14 — The number of evolvable core links and that of robust neighbor links for asynchronous update. (A) The number of evolvable core links for asynchronous update, the number of links included in the intersection with the original evolvable core for synchronous update, and the number of links in the control. (B) The number of robust neighbor links for asynchronous update, the number of links included in the intersection with the original robust neighbor for synchronous update, and the number of links in the control. Here, the control (random intersection) is the intersection between the random subgroup of links of the same size with the original evolvable core (or the original robust neighbor) and another random subgroup of links of the same size with the newly obtained evolvable core (or the newly obtained robust neighbor) for asynchronous update. In this comparison, we used the first seed for random deletion order and random initial states as shown in Figure S3 for the original evolvable core and robust neighbor for synchronous update. P-values were obtained from one-sided two sample Chi square tests for 2 (the number of evolvable core links or robust neighbor links for synchronous update)×2 (the number of evolvable core links or robust neighbor links for asynchronous update) contingency tables. (TIF) [file pcbi.1003763.s014.tif]

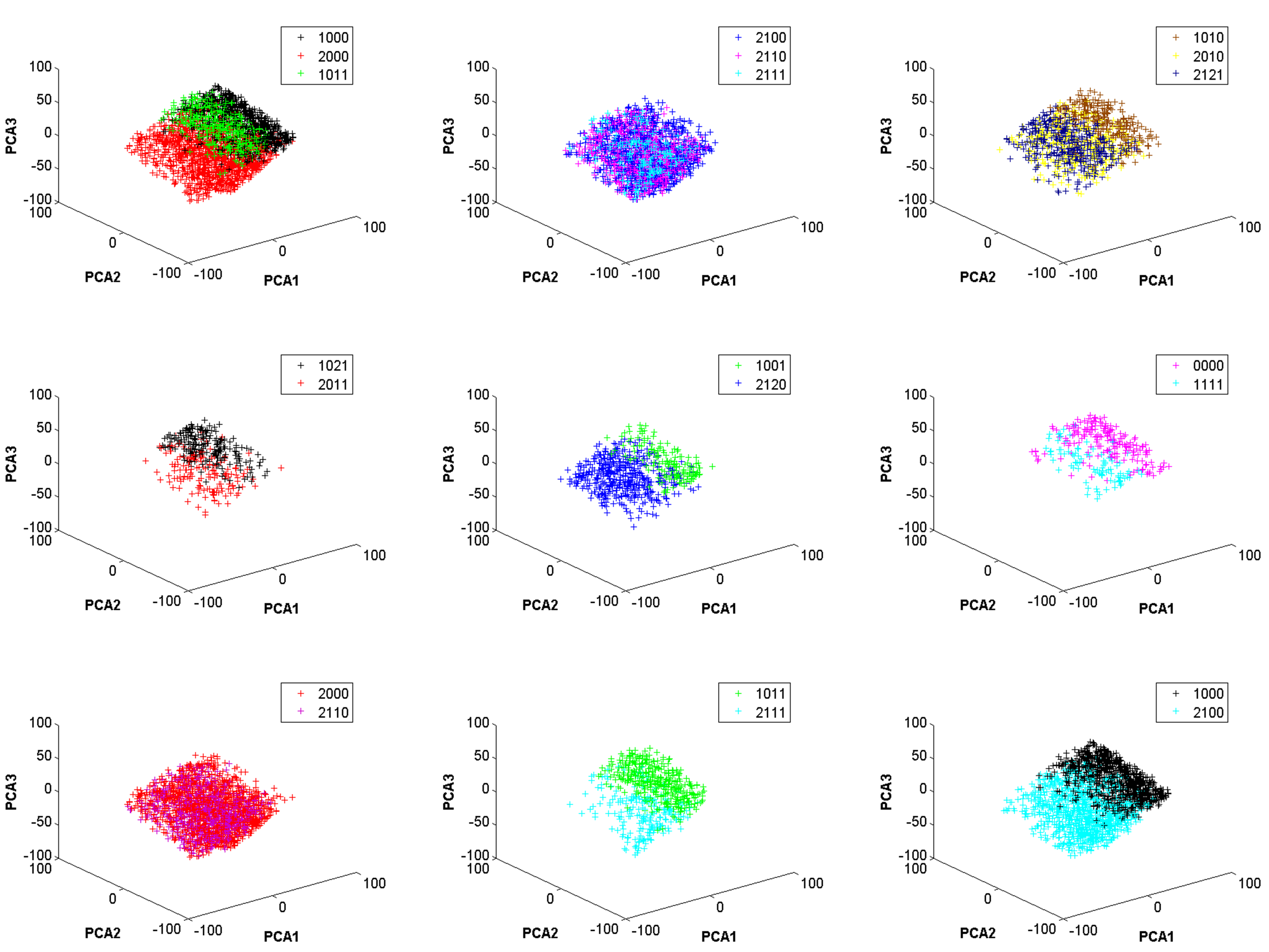

Supplement: Figure S15 — The emergent function of information processing in the evolvable core of the human signaling network. We followed the same procedure proposed by Helikar et al. [22]. These scatter plots show how the network system clusters combinations of 10,000 random inputs and then maps them to the global outputs. The input values (0∼100%) of three input nodes (‘EGF’, ‘ECM’, and ‘ExtPump’) associated with the 15 most common outputs of Helikar et al. [22] are plotted in 3-dimensional space using principle component analysis, where the input value denotes a percentage ‘ON’ for the input node in the Boolean iteration of 1,000 times and the output value denotes the average number of ‘ON’s over the last 100 iterations out of the Boolean iteration of 1,000 times. The output values were categorized by using three different ranges: 0 (0∼9%), 1 (10∼29%), and 2 (30∼100%). A four-tuple of numbers in the legends represents a category of the four output nodes (‘Akt’, ‘Erk’, ‘Rac’, and ‘Cdc42’). (TIF) [file pcbi.1003763.s015.tif]

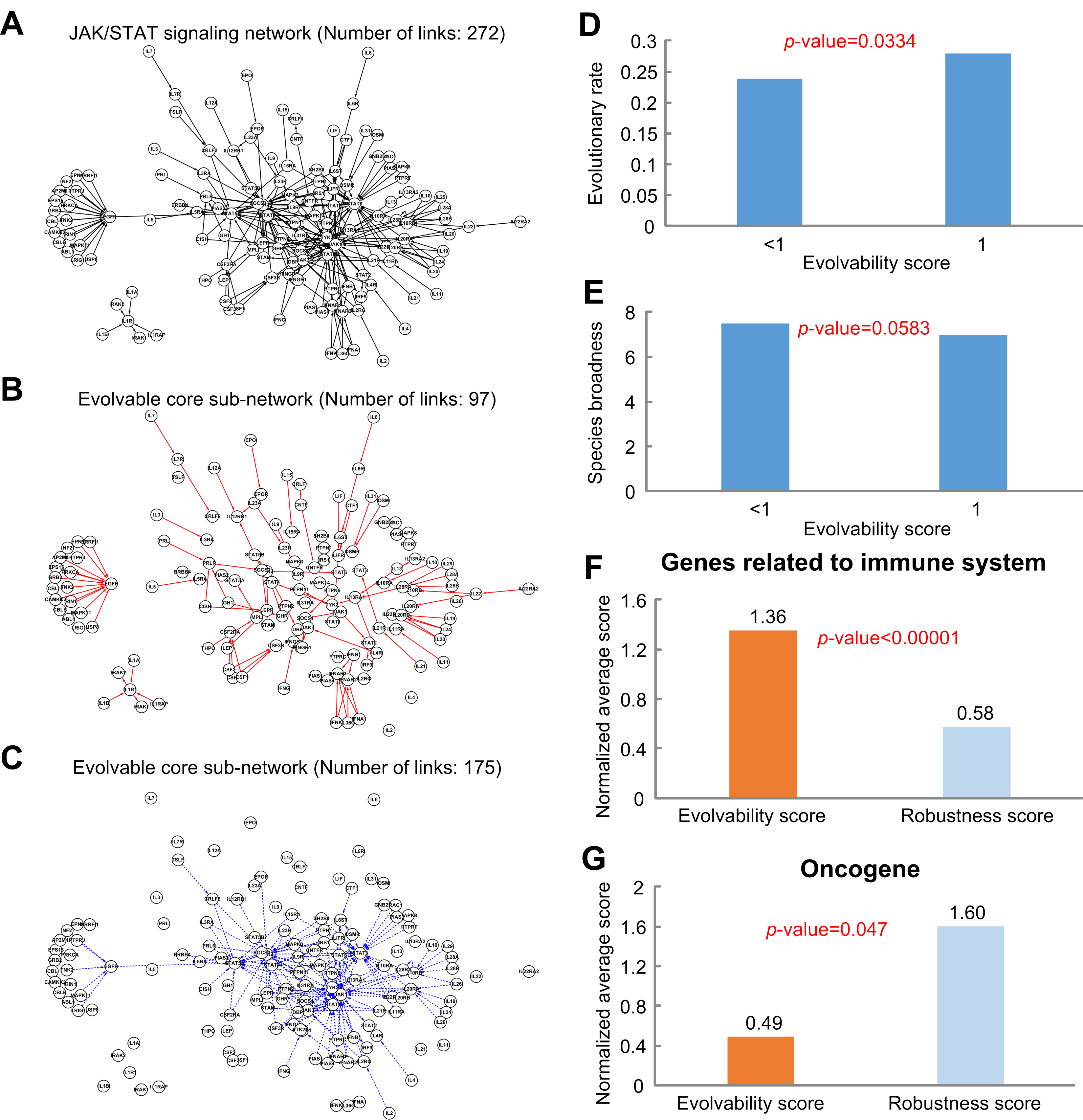

Supplement: Figure S16 — Decomposition of the JAK/STAT signaling network. (A) JAK/STAT signaling network. (B) Evolvable core. (C) Robust neighbor. (D) The correlation between evolutionary rate and evolvability score. (E) The correlation between species broadness and evolvability score. (F) The normalized average evolvability and robustness scores of the genes related to immune system. (G) The normalized average evolvability and robustness scores of the oncogenes. (TIF) [file pcbi.1003763.s016.tif]

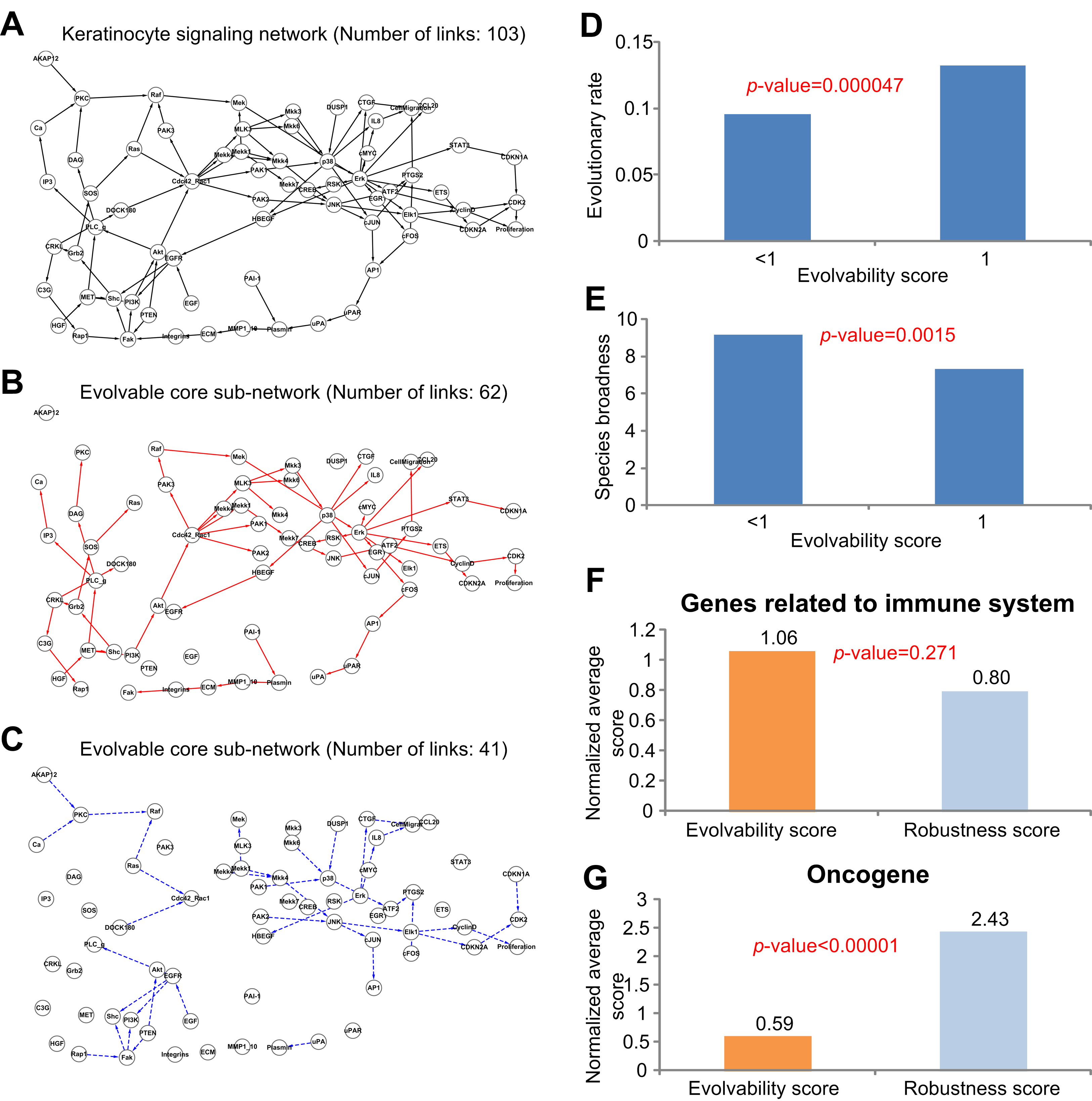

Supplement: Figure S17 — Decomposition of the Keratinocyte signaling network. (A) Keratinocyte signaling network. (B) Evolvable core. (C) Robust neighbor. (D) The correlation between evolutionary rate and evolvability score. (E) The correlation between species broadness and evolvability score. (F) The normalized average evolvability and robustness scores of the genes related to immune system. (G) The normalized average evolvability and robustness scores of the oncogenes. (TIF) [file pcbi.1003763.s017.tif]

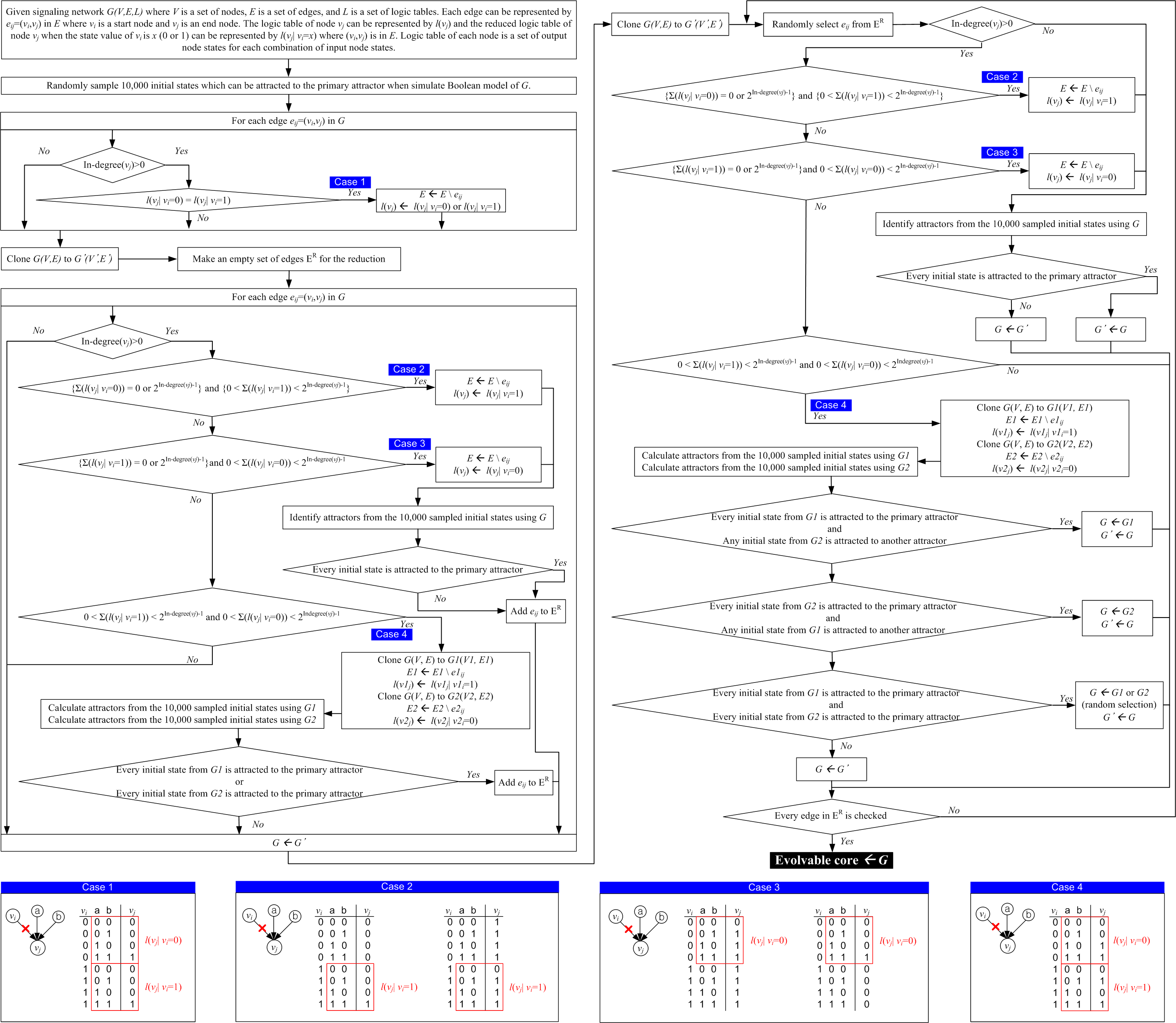

Supplement: Figure S18 — A flow diagram illustrating the proposed algorithm for identifying the evolvable core and robust neighbor. (TIF) [file pcbi.1003763.s018.tif]
