# Supplementary material for: Robustness and Evolvability of the Human Signaling Network
Source: PLoS Comput Biol. 2014 Jul 31;10(7):e1003763. doi: 10.1371/journal.pcbi.1003763 (PMC4117429; doi:10.1371/journal.pcbi.1003763)
Supplement: Table S2 — List of links in the evolvable core for the first seed of deletion order and the first seed of initial states as shown in Figure S3. (DOC) [file pcbi.1003763.s020.doc]

**Table S2**. List of links in the evolvable core for the first seed of deletion order and the first seed of initial states as shown in Figure S3.

| Name of source node | Name of target node | Name of source node | Name of target node |
| --- | --- | --- | --- |
| EGF | EGFR | B_Arrestin | Src |
| PKC | EGFR | Gai | Src |
| Ca | EGFR | Gas | Src |
| α_q_R | EGFR | α_s_R | Src |
| α_i_R | EGFR | Fak | Src |
| α_12_13_R | EGFR | PTP1b | Src |
| PKA | PLC_B | Cas | Src |
| Gaq | PLC_B | PTPa | Src |
| Gbg_i | PLC_B | EGFR | Src |
| PLC_B | PLC_B | SHP2 | Csk |
| PA | PLC_g | Cbp | Csk |
| AA | PLC_g | PKA | Csk |
| Src | PLC_g | Gbg_i | Csk |
| Fak | PLC_g | Gbg_q | Csk |
| EGFR | PLC_g | Gbg_12_13 | Csk |
| PIP3_345 | PLC_g | PAK | Myosin |
| PIP2_45 | IP3 | Arp_2_3 | Actin |
| PLC_B | IP3 | Myosin | Actin |
| PLC_g | IP3 | ILK | Integrins |
| DGK | DAG | Src | Integrins |
| DAG | DAG | Integrins | Integrins |
| PIP2_45 | DAG | PP2A | Integrins |
| PLC_B | DAG | ECM | Integrins |
| PLC_g | DAG | Talin | Integrins |
| ExtPump | Ca | PKC | RKIP |
| IP3R1 | Ca | Vinc | Vinc |
| Ca | CaM | Talin | Vinc |
| CaM | CaMKK | PTPPEST | Crk |
| CaM | CaMK | Src | Crk |
| CaMKK | CaMK | Fak | Crk |
| PIP3_345 | RGS | Cas | Crk |
| CaM | RGS | Cas | Nck |
| Src | Rap1 | PIP3_345 | DOCK180 |
| PTEN | Shc | RhoGDI | Rac |
| Shc | Shc | p190RhoGAP | Rac |
| Fak | Shc | RalBP1 | Rac |
| Src | Shc | Rac | Rac |
| EGFR | Shc | Integrins | Rac |
| Fak | Grb2 | ECM | Rac |
| Src | Grb2 | PAK | Rac |
| Shc | Grb2 | Tiam | Rac |
| EGFR | Grb2 | RasGRF_GRP | Rac |
| Erk | Sos | PIP2_34 | Tiam |
| PIP3_345 | Sos | B_Arrestin | RalGDS |
| Nck | Sos | Cas | AND_3_4 |
| Crk | Sos | CaM | Ral |
| Grb2 | Sos | Ral | RalBP1 |
| SHP2 | Ras | RhoGDI | Cdc42 |
| RasGRF_GRP | Ras | Cdc42 | Cdc42 |
| Sos | Ras | Rac | Cdc42 |
| PP2A | PKC | Src | Cdc42 |
| Trx | PKC | Pix_Cool | Cdc42 |
| PKC | PKC | Gbg_i | Cdc42 |
| PKC_primed | PKC | PAK | Cdc42 |
| AA | PKC | TAK1 | NIK |
| DAG | PKC | Src | Talin |
| Ca | PKC | Talin | Talin |
| PLA2 | AA | PIP2_45 | Talin |
| Erk | Raf | PIP3_345 | ILK |
| Akt | Raf | WASP | Arp_2_3 |
| PKA | Raf | B_Parvin | Pix_Cool |
| Raf | Raf | PIP2_34 | Pix_Cool |
| Ras | Raf | PIP3_345 | Pix_Cool |
| Raf_Loc | Raf | PKA | PAK |
| Src | Raf | PTP1b | PAK |
| RKIP | Raf | Akt | PAK |
| PAK | Raf | Nck | PAK |
| PP2A | Mek | Grb2 | PAK |
| Mek | Mek | Src | PAK |
| Mekk1 | Mek | PAK | PAK |
| Mekk2 | Mek | Cdc42 | PAK |
| Mekk3 | Mek | Rac | PAK |
| Raf | Mek | Fak | WASP |
| Tpl2 | Mek | Fak | Graf |
| MKPs | Erk | CaM | MLCK |
| PP2A | Erk | PKA | MLCP |
| Mek | Erk | IL1_TNF | IL1_TNFR |
| Erk | Erk | IL1_TNFR | Trafs |
| PIP2_45 | PLA2 | Trafs | GCK |
| PIP3_345 | PLA2 | SHP2 | Gab1 |
| Ca | PLA2 | Grb2 | Gab1 |
| Erk | PLA2 | EGFR | Gab1 |
| CaMK | PLA2 | Gab1 | Gab1 |
| Gbg_i | PI3K | PIP3_345 | Gab1 |
| Crk | PI3K | Trafs | Tab_1_2 |
| Fak | PI3K | Cdc42 | Mekk1 |
| Src | PI3K | Ras | Mekk1 |
| EGFR | PI3K | GCK | Mekk1 |
| Ras | PI3K | Grb2 | Mekk1 |
| Gab1 | PI3K | Shc | Mekk1 |
| PP2A | Akt | EGFR | Mekk2 |
| Akt | Akt | IL1_TNFR | Mekk3 |
| PIP2_34 | Akt | Cdc42 | Mekk4 |
| PIP3_345 | Akt | Trx | ASK1 |
| PDK1 | Akt | Trafs | Trx |
| CaMKK | Akt | Stress | Trx |
| ILK | Akt | Tab_1_2 | TAK1 |
| Src | Akt | Trafs | Tpl2 |
| Src | PDK1 | Cdc42 | MLK1 |
| p90RSK | PDK1 | SAPK | MLK2 |
| PIP_4 | PIP_4 | Rac | MLK3 |
| PIP2_45 | PIP2_45 | Stress | TAO_1_2 |
| PI4K | PIP2_45 | ASK1 | Sek1 |
| PI5K | PIP2_45 | ASK1 | MKK7 |
| PIP3_345 | PIP2_45 | ASK1 | MKK3 |
| PTEN | PIP2_45 | ASK1 | MKK6 |
| PI5K | PIP2_34 | SAPK | SAPK |
| PTEN | PIP2_34 | MKK7 | SAPK |
| PIP2_34 | PIP2_34 | Sek1 | SAPK |
| PI3K | PIP2_34 | PP2A | p38 |
| PI4K | PIP2_34 | p90RSK | p90RSK |
| PTEN | PIP3_345 | Erk | p90RSK |
| PIP3_345 | PIP3_345 | PDK1 | p90RSK |
| PI3K | PIP3_345 | EGFR | PP2A |
| PIP2_45 | PIP3_345 | Gab1 | SHP2 |
| PI5K | PIP3_345 | cAMP | MKPs |
| PIP2_34 | PIP3_345 | SAPK | MKPs |
| Rho | PI4K | p38 | MKPs |
| PKC | PI4K | Erk | MKPs |
| Gai | PI4K | Src | PTEN |
| Gaq | PI4K | PTEN | PTEN |
| ARF | PI4K | Stress | PTEN |
| RhoK | PI5K | Rho | PTEN |
| PA | PI5K | PI3K | PTEN |
| ARF | PI5K | Cdc42 | PTEN |
| PI5K | PI5K | Pix_Cool | PTEN |
| Talin | PI5K | Stress | PTP1b |
| Src | PI5K | PKC | PTPPEST |
| Fak | PI5K | PKA | PTPPEST |
| B_Arrestin | α_s_R | Integrins | PTPPEST |
| Pα_s_R | α_s_R | ECM | PTPPEST |
| α_s_R | α_s_R | PKC | PTPa |
| α_s_lig | α_s_R | Actin | PLD |
| Gas | Gbg_s | PIP3_345 | PLD |
| Gbg_s | Gbg_s | PIP2_45 | PLD |
| α_s_R | Gbg_s | ARF | PLD |
| Gbg_i | Gai | PKC | PLD |
| RGS | Gai | PLD | PA |
| PKA | Gai | PIP3_345 | ARF |
| Gai | Gai | PIP2_45 | ARF |
| α_s_lig | Gai | EGFR | DGK |
| α_s_R | Gai | DAG | DGK |
| α_i_R | Gai | PKC | DGK |
| Gbg_s | Gas | Src | DGK |
| PKA | Gas | Ca | DGK |
| RGS | Gas | PA | DGK |
| Gas | Gas | ILK | B_Parvin |
| α_s_R | Gas | B_Arrestin | α_i_R |
| Gbg_q | Gaq | Pα_i_R | α_i_R |
| RGS | Gaq | α_i_R | α_i_R |
| PLC_B | Gaq | α_i_lig | α_i_R |
| Gaq | Gaq | B_Arrestin | α_q_R |
| α_q_R | Gaq | Pα_i_R | α_q_R |
| RKIP | GRK | α_q_R | α_q_R |
| Erk | GRK | α_q_lig | α_q_R |
| Src | GRK | B_Arrestin | α_12_13_R |
| B_Arrestin | GRK | Pα_12_13_R | α_12_13_R |
| PIP2_45 | GRK | α_12_13_R | α_12_13_R |
| Gbg_s | GRK | α_12_13_lig | α_12_13_R |
| Gbg_i | GRK | Gbg_12_13 | Ga_12_13 |
| Gbg_q | GRK | p115RhoGEF | Ga_12_13 |
| Gbg_12_13 | GRK | Ga_12_13 | Ga_12_13 |
| Pα_s_R | B_Arrestin | α_12_13_R | Ga_12_13 |
| Pα_q_R | B_Arrestin | Gai | Gbg_i |
| Pα_i_R | B_Arrestin | Gbg_i | Gbg_i |
| Pα_12_13_R | B_Arrestin | α_i_R | Gbg_i |
| ECM | AC | Gaq | Gbg_q |
| Integrins | AC | Gbg_q | Gbg_q |
| Gbg_i | AC | α_q_R | Gbg_q |
| Gas | AC | Ga_12_13 | Gbg_12_13 |
| PDE4 | cAMP | Gbg_12_13 | Gbg_12_13 |
| cAMP | cAMP | α_12_13_R | Gbg_12_13 |
| AC | cAMP | Erk | PDE4 |
| PP2A | PKA | B_Arrestin | PDE4 |
| PKA | PKA | PKA | PDE4 |
| cAMP | PKA | SHP2 | Cbp |
| PDK1 | PKA | Src | Cbp |
| Cdc42 | RasGRF_GRP | PP2A | IP3R1 |
| DAG | RasGRF_GRP | CaM | IP3R1 |
| CaM | RasGRF_GRP | Ca | IP3R1 |
| Src | p120RasGAP | IP3R1 | IP3R1 |
| PKA | Rho | IP3 | IP3R1 |
| p190RhoGAP | Rho | Gbg_i | IP3R1 |
| RhoGDI | Rho | PKA | IP3R1 |
| Rho | Rho | Raf_Loc | Raf_DeP |
| p115RhoGEF | Rho | Raf_DeP | Raf_DeP |
| PKC | RhoGDI | Raf_Rest | Raf_DeP |
| PIP2_45 | RhoGDI | PP2A | Raf_DeP |
| AA | RhoGDI | PKC | PKC_primed |
| Src | p190RhoGAP | PKC_primed | PKC_primed |
| PIP3_345 | p115RhoGEF | PDK1 | PKC_primed |
| Ga_12_13 | p115RhoGEF | Raf | Raf_Loc |
| Rho | RhoK | Raf_Loc | Raf_Loc |
| PTEN | Fak | Raf_DeP | Raf_Loc |
| Src | Fak | Ras | Raf_Loc |
| Fak | Fak | Raf_DeP | Raf_Rest |
| Talin | Fak | GRK | Pα_s_R |
| Integrins | Fak | α_s_R | Pα_s_R |
| PTPPEST | Cas | GRK | Pα_q_R |
| Cas | Cas | α_q_R | Pα_q_R |
| Src | Cas | GRK | Pα_i_R |
| Fak | Cas | α_i_R | Pα_i_R |
| Csk | Src | GRK | Pα_12_13_R |
| Src | Src | α_12_13_R | Pα_12_13_R |
