# Supplementary material for: Robustness and Evolvability of the Human Signaling Network
Source: PLoS Comput Biol. 2014 Jul 31;10(7):e1003763. doi: 10.1371/journal.pcbi.1003763 (PMC4117429; doi:10.1371/journal.pcbi.1003763)
Supplement: Table S3 — List of links in the robust neighbor for the first seed of deletion order and the first seed of initial states as shown in Figure S3. (DOC) [file pcbi.1003763.s021.doc]

**Table S3**. List of links in the robust neighbor for the first seed of deletion order and the first seed of initial states as shown in Figure S3.

| Name of source node | Name of target node | Name of source node | Name of target node |
| --- | --- | --- | --- |
| EGFR | EGFR | Nck | WASP |
| PTP1b | EGFR | Grb2 | WASP |
| PKA | CaMKK | PIP2_45 | WASP |
| PP2A | CaMK | Src | Graf |
| Gai | Rap1 | PAK | MLCK |
| Rap1 | Rap1 | PKA | MLCK |
| cAMP | Rap1 | Erk | MLCK |
| PKA | Rap1 | RhoK | MLCP |
| CaMK | Rap1 | PKC | MLCP |
| PIP2_45 | Sos | ILK | MLCP |
| Ras | Sos | PAK | MLCP |
| p120RasGAP | Ras | Raf | MLCP |
| PAK | Mek | p38 | Tab_1_2 |
| PI3K | PIP_4 | NIK | Mekk1 |
| PI5K | PIP_4 | Rho | Mekk1 |
| PIP2_34 | PIP_4 | Rac | Mekk1 |
| PTEN | PIP_4 | Trafs | Mekk1 |
| PI4K | PIP_4 | Mekk2 | Mekk2 |
| PI3K | PIP2_45 | Src | Mekk2 |
| PLC_B | PIP2_45 | PI3K | Mekk2 |
| PLC_g | PIP2_45 | Grb2 | Mekk2 |
| Rho | PI5K | PLC_g | Mekk2 |
| Rac | PI5K | Gab1 | Mekk3 |
| PKC | GRK | Trafs | Mekk3 |
| PKC | AC | Rac | Mekk3 |
| Fak | p120RasGAP | Rac | Mekk4 |
| SHP2 | p120RasGAP | Akt | ASK1 |
| Ca | p120RasGAP | Rac | MLK1 |
| PIP2_45 | p120RasGAP | Cdc42 | MLK2 |
| PIP2_34 | p120RasGAP | Rac | MLK2 |
| PIP3_345 | p120RasGAP | IL1_TNFR | MLK3 |
| EGFR | p120RasGAP | Cdc42 | MLK3 |
| Graf | Rho | Tpl2 | Sek1 |
| p120RasGAP | p190RhoGAP | TAK1 | Sek1 |
| p190RhoGAP | p190RhoGAP | MLK3 | Sek1 |
| Fak | p190RhoGAP | MLK2 | Sek1 |
| PKA | Src | MLK1 | Sek1 |
| Src | Csk | Mekk4 | Sek1 |
| Fak | Csk | Mekk3 | Sek1 |
| MLCP | Myosin | Mekk2 | Sek1 |
| Myosin | Myosin | Mekk1 | Sek1 |
| RhoK | Myosin | MLK3 | MKK7 |
| ILK | Myosin | MLK2 | MKK7 |
| MLCK | Myosin | MLK1 | MKK7 |
| CaM | Myosin | Mekk4 | MKK7 |
| PIP2_45 | Vinc | Mekk3 | MKK7 |
| Src | Vinc | Mekk2 | MKK7 |
| Actin | Vinc | Mekk1 | MKK7 |
| EGFR | Nck | PAK | MKK3 |
| Crk | DOCK180 | TAO_1_2 | MKK3 |
| Cas | DOCK180 | Tpl2 | MKK3 |
| Akt | Rac | TAK1 | MKK3 |
| Gbg_i | Rac | MLK3 | MKK3 |
| Cdc42 | Rac | MLK2 | MKK3 |
| Pix_Cool | Rac | MLK1 | MKK3 |
| DOCK180 | Rac | Mekk4 | MKK3 |
| Rap1 | Tiam | Mekk3 | MKK3 |
| Ras | Tiam | Mekk2 | MKK3 |
| PIP2_45 | Tiam | PAK | MKK6 |
| PIP3_345 | Tiam | TAO_1_2 | MKK6 |
| CaMK | Tiam | Tpl2 | MKK6 |
| Src | Tiam | TAK1 | MKK6 |
| PKC | Tiam | Mekk4 | MKK6 |
| PKC | RalGDS | MLK3 | MKK6 |
| PDK1 | RalGDS | MKPs | SAPK |
| Ras | RalGDS | PP2A | SAPK |
| PIP3_345 | RalGDS | MKPs | p38 |
| α_12_13_R | RalGDS | p38 | p38 |
| α_s_R | RalGDS | Sek1 | p38 |
| α_q_R | RalGDS | MKK6 | p38 |
| α_i_R | RalGDS | MKK3 | p38 |
| AND_3_4 | Ral | Src | PP2A |
| RalGDS | Ral | PP2A | PP2A |
| RalBP1 | Cdc42 | cAMP | PP2A |
| p190RhoGAP | Cdc42 | EGF | PTP1b |
| Graf | Cdc42 | EGFR | PTP1b |
| Nck | NIK | Ral | PLD |
| Tpl2 | NIK | Rac | PLD |
| PKC | Talin | Rho | PLD |
| PDK1 | PAK | Cdc42 | PLD |
| PTPPEST | WASP | PKC | IP3R1 |
| Cdc42 | WASP | Raf_Rest | Raf_Rest |
| Crk | WASP | Raf | Raf_Rest |
| Src | WASP |  |  |
