# Supplementary material for: Robustness and Evolvability of the Human Signaling Network
Source: PLoS Comput Biol. 2014 Jul 31;10(7):e1003763. doi: 10.1371/journal.pcbi.1003763 (PMC4117429; doi:10.1371/journal.pcbi.1003763)
Supplement: Table S4 — The name, EnetrezGene IDs, degree, evolvability score, and robustness score for each node of the network. (DOC) [file pcbi.1003763.s022.doc]

**Table S4**. The name, EnetrezGene IDs, degree, evolvability score, and robustness score for each node of the network.

| Name | EntrezGene ID | Degree in the original network | Evolvability score | Robustness score |
| --- | --- | --- | --- | --- |
| AA |  | 4 | 1 | 0 |
| α_12_13_lig | 2147 | 1 | 1 | 0 |
| α_i_lig | 10344;10563;10850;2919;2920;2921;3576;3627;388372;414062;4283;5196;5197;5473;56477;58191;6346;6347;6348;6349;6351;6352;6354;6355;6356;6357;6358;6359;6360;6361;6362;6363;6364;6366;6367;6368;6369;6370;6372;6373;6374;6375;6376;6387;6846;9547;9560 | 1 | 1 | 0 |
| α_q_lig |  | 1 | 1 | 0 |
| α_s_lig |  | 2 | 1 | 0 |
| ARF | 1029 | 5 | 1 | 0 |
| Arp_2_3 | 10092;10093;10094;10095;10109;10552;653888;81873 | 2 | 1 | 0 |
| B_Arrestin | 408;409 | 12 | 1 | 0 |
| B_Parvin | 29780 | 2 | 1 | 0 |
| Cbp | 55824 | 3 | 1 | 0 |
| DAG |  | 9 | 1 | 0 |
| DGK | 1606;1607;1608;160851;1609;8525;8526;8527;9162 | 7 | 1 | 0 |
| ECM | 10319;1101;1277;1278;1280;1281;1282;1284;1286;1287;1288;1289;1290;1291;1292;1293;1301;1302;1311;131873;22798;2335;284217;3371;3381;3908;3909;3910;3911;3912;3913;3914;3915;3918;50509;5649;63923;6696;7057;7058;7059;7060;7143;7148;7448;7450 | 4 | 1 | 0 |
| ExtPump | 490;491;491;493 | 1 | 1 | 0 |
| Ga_12_13 | 10672;2768 | 7 | 1 | 0 |
| Gaq | 2767;2769;2776;9630 | 9 | 1 | 0 |
| Gas | 2774;2778 | 9 | 1 | 0 |
| Gbg_12_13 | 10681;2782;2783;2784;2785;2786;2787;2788;2790;2791;2792;2793;51764;54331;55970;59345;94235 | 7 | 1 | 0 |
| Gbg_q | 10681;2782;2783;2784;2785;2786;2787;2788;2790;2791;2792;2793;51764;54331;55970;59345;94235 | 7 | 1 | 0 |
| Gbg_s | 10681;2782;2783;2784;2785;2786;2787;2788;2790;2791;2792;2793;51764;54331;55970;59345;94235 | 6 | 1 | 0 |
| GCK | 5871 | 2 | 1 | 0 |
| IL1_TNF | 7124;3552;3553 | 1 | 1 | 0 |
| Integrins | 22801;3655;3672;3673;3674;3675;3676;3678;3679;3680;3685;8515;8516;3688;3690;3691;3693;3694;3695;3696 | 11 | 1 | 0 |
| IP3 |  | 4 | 1 | 0 |
| p115RhoGEF | 9138 | 4 | 1 | 0 |
| p90RSK | 27330;6195;6196;6197 | 5 | 1 | 0 |
| PA |  | 4 | 1 | 0 |
| Pα_12_13_R | 2149;929 | 4 | 1 | 0 |
| Pα_i_R | 10663;10803;1230;1232;1233;1234;1235;1236;1237;1524;2826;2829;2833;3577;3579;643;729230;7852 | 5 | 1 | 0 |
| Pα_q_R | 10800;1128;1129;1131;146;147;148;185;1909;1910;2149;2911;2915;2925;3269;3356;3357;3358;3973;4923;5021;552;553;56413;57105;5724;5731;5733;5737;623;624;6865;6869;6870;6915;7201;886;887 | 3 | 1 | 0 |
| Pα_s_R | 1128;1131;1133;135;136;153;154;155;1812;1816;3274;3360;3361;3362;3363 | 4 | 1 | 0 |
| PDE4 | 5141;5142;5143;5144 | 4 | 1 | 0 |
| PKC_primed | 5578;5579;5582 | 5 | 1 | 0 |
| PLA2 | 100137049;123745;26279;30814;391013;50487;5319;5320;5321;5322;64600;81579;8398;8399;84647;8681 | 6 | 1 | 0 |
| PTPa | 5786 | 2 | 1 | 0 |
| Raf_DeP | 5894 | 7 | 1 | 0 |
| Raf_Loc | 5894 | 7 | 1 | 0 |
| RasGRF_GRP | 5923;5924;10125;10235;115727;25780 | 5 | 1 | 0 |
| RGS | 6004;6002;8787;9628;5999;5997;5998;10287;6000;6003;8490;8601;64407;5996;6001;85397;431704;26575;26166 | 5 | 1 | 0 |
| RhoGDI | 396;397 | 6 | 1 | 0 |
| RKIP | 5037 | 3 | 1 | 0 |
| Shc | 25759;399694;53358;6464 | 8 | 1 | 0 |
| Stress |  | 4 | 1 | 0 |
| Trx | 7295 | 4 | 1 | 0 |
| AC | 107;108;109;113;114;115;196883 | 6 | 0.833 | 0.167 |
| Actin | 58 | 4 | 0.75 | 0.25 |
| α_12_13_R | 2149;929 | 10 | 0.9 | 0.1 |
| α_i_R | 10663;10803;1230;1232;1233;1234;1235;1236;1237;1524;2826;2829;2833;3577;3579;643;729230;7852 | 10 | 0.9 | 0.1 |
| α_q_R | 10800;1128;1129;1131;146;147;148;185;1909;1910;2149;2911;2915;2925;3269;3356;3357;3358;3973;4923;5021;552;553;56413;57105;5724;5731;5733;5737;623;624;6865;6869;6870;6915;7201;886;887 | 10 | 0.9 | 0.1 |
| α_s_R | 1128;1131;1133;135;136;153;154;155;1812;1816;3274;3360;3361;3362;3363 | 11 | 0.909 | 0.091 |
| AND_3_4 | 8412 | 2 | 0.5 | 0.5 |
| ASK1 | 4217 | 6 | 0.833 | 0.167 |
| Ca |  | 9 | 0.889 | 0.111 |
| CaM | 801;805;808 | 9 | 0.889 | 0.111 |
| CaMKK | 10645;84254 | 4 | 0.75 | 0.25 |
| Cas | 9564 | 10 | 0.9 | 0.1 |
| EGF | 1950 | 2 | 0.5 | 0.5 |
| Erk | 5594;5595 | 13 | 0.923 | 0.077 |
| Gab1 | 2549 | 9 | 0.889 | 0.111 |
| Gai | 2770;2771;2773 | 12 | 0.917 | 0.083 |
| Gbg_i | 10681;2782;2783;2784;2785;2786;2787;2788;2790;2791;2792;2793;51764;54331;55970;59345;94235 | 13 | 0.923 | 0.077 |
| GRK | 156;131890;157;2868;2869;2870;6011 | 14 | 0.929 | 0.071 |
| IL1_TNFR | 7132;3554;3556 | 4 | 0.75 | 0.25 |
| IP3R1 | 3708 | 10 | 0.9 | 0.1 |
| Mek | 5604;5605 | 10 | 0.9 | 0.1 |
| PI4K | 5297;5298;55300;55361 | 8 | 0.875 | 0.125 |
| Pix_Cool | 9459 | 6 | 0.833 | 0.167 |
| PLC_B | 23236;5330;5331;5332 | 9 | 0.889 | 0.111 |
| PTEN | 5728 | 14 | 0.929 | 0.071 |
| PTPPEST | 5782 | 7 | 0.857 | 0.143 |
| RalBP1 | 10928 | 3 | 0.667 | 0.333 |
| SHP2 | 5781 | 6 | 0.833 | 0.167 |
| Tab_1_2 | 10454;23118 | 3 | 0.667 | 0.333 |
| Talin | 7094;83660 | 9 | 0.889 | 0.111 |
| Akt | 10000;207;208 | 13 | 0.846 | 0.154 |
| cAMP |  | 8 | 0.75 | 0.25 |
| Crk | 1398;1399 | 8 | 0.75 | 0.25 |
| Csk | 1445 | 9 | 0.778 | 0.222 |
| Grb2 | 2885 | 10 | 0.8 | 0.2 |
| ILK | 3611 | 6 | 0.667 | 0.333 |
| MKPs | 11072;11221;1843;1844;1845;1846;1847;1848;1849;1850;1852;56940;80824 | 7 | 0.714 | 0.286 |
| PDK1 | 5163 | 8 | 0.75 | 0.25 |
| PIP2_34 |  | 12 | 0.833 | 0.167 |
| PLC_g | 5335;5336 | 10 | 0.8 | 0.2 |
| Raf | 5894 | 14 | 0.857 | 0.143 |
| RhoK | 6011 | 4 | 0.5 | 0.5 |
| SAPK | 5601 | 8 | 0.75 | 0.25 |
| Sos | 6654;6655 | 8 | 0.75 | 0.25 |
| TAO_1_2 | 51347;57551;9344 | 3 | 0.333 | 0.667 |
| Trafs | 7185;7186;7187;7188;7189;9618 | 7 | 0.714 | 0.286 |
| CaMK | 814;815;816;817;818 | 6 | 0.5 | 0.5 |
| DOCK180 | 1793 | 4 | 0.25 | 0.75 |
| Fak | 5747 | 19 | 0.842 | 0.158 |
| Graf | 23092;79658;143872 | 4 | 0.25 | 0.75 |
| Nck | 4690;8440 | 6 | 0.5 | 0.5 |
| NIK | 9020 | 4 | 0.25 | 0.75 |
| PI3K | 23533;5290;5291;5293;5294;5295;5296;8503 | 13 | 0.769 | 0.231 |
| PI5K | 200576;23396;5305;79837;8394;8395;8396 | 14 | 0.786 | 0.214 |
| PIP3_345 |  | 23 | 0.870 | 0.130 |
| PTP1b | 5770 | 6 | 0.5 | 0.5 |
| Raf_Rest | 5894 | 5 | 0.4 | 0.6 |
| Ral | 5898;5899 | 5 | 0.4 | 0.6 |
| TAK1 | 7182 | 5 | 0.4 | 0.6 |
| Vinc | 7414 | 6 | 0.5 | 0.5 |
| MLCK | 4638;85366;91807 | 5 | 0.2 | 0.8 |
| MLK1 | 4293 | 5 | 0.2 | 0.8 |
| PKA | 5566;5567;5568 | 20 | 0.8 | 0.2 |
| PLD | 5337;5338 | 10 | 0.6 | 0.4 |
| Ras | 3265;3845;4893 | 11 | 0.636 | 0.364 |
| Rho | 29984;399;57381;58480;390;388;389;171177;387;391;23433;54509 | 13 | 0.692 | 0.308 |
| Tpl2 | 1326 | 6 | 0.333 | 0.667 |
| Mekk4 | 4216 | 6 | 0.167 | 0.833 |
| MLK2 | 4294 | 6 | 0.167 | 0.833 |
| p190RhoGAP | 2909 | 8 | 0.375 | 0.625 |
| PIP_4 |  | 7 | 0.286 | 0.714 |
| EGFR | 1956 | 21 | 0.714 | 0.286 |
| Mekk1 | 4214 | 12 | 0.5 | 0.5 |
| Mekk3 | 4215 | 8 | 0.25 | 0.75 |
| MLCP | 4659;4660;5499;5500;5501 | 7 | 0.143 | 0.857 |
| MLK3 | 4296 | 7 | 0.143 | 0.857 |
| PAK | 10298;5058;5062;5063;56924;57144 | 20 | 0.7 | 0.3 |
| PP2A | 5515;5516;5518;5519 | 16 | 0.625 | 0.375 |
| MKK6 | 5608 | 8 | 0.125 | 0.875 |
| MKK7 | 5609 | 9 | 0.222 | 0.778 |
| Myosin | 10398;140465;4637;4629 | 9 | 0.222 | 0.778 |
| p38 | 1432;5600;5603;6300 | 9 | 0.222 | 0.778 |
| PKC | 5578;5579;5582 | 24 | 0.708 | 0.292 |
| Rap1 | 5906;5908 | 8 | 0.125 | 0.875 |
| Tiam | 7074;26230 | 9 | 0.222 | 0.778 |
| WASP | 7454;8976 | 9 | 0.222 | 0.778 |
| Cdc42 | 998 | 22 | 0.636 | 0.364 |
| PIP2_45 |  | 23 | 0.652 | 0.348 |
| Src | 6714 | 42 | 0.810 | 0.190 |
| Mekk2 | 10746 | 11 | 0.182 | 0.818 |
| p120RasGAP | 5921 | 10 | 0.1 | 0.9 |
| RalGDS | 5900 | 10 | 0.1 | 0.9 |
| Sek1 | 6416 | 12 | 0.167 | 0.833 |
| MKK3 | 5606 | 12 | 0.083 | 0.917 |
| Rac | 5879;5880;5881 | 25 | 0.52 | 0.48 |
