# Supplementary material for: Robustness and Evolvability of the Human Signaling Network
Source: PLoS Comput Biol. 2014 Jul 31;10(7):e1003763. doi: 10.1371/journal.pcbi.1003763 (PMC4117429; doi:10.1371/journal.pcbi.1003763)
Supplement: Table S5 — The standard deviation of the evolvability scores of the targets of multi-target drug. (DOC) [file pcbi.1003763.s023.doc]

**Table S5**. The standard deviation of the evolvability scores of the targets of multi-target drug.

| Drug name | Drug type | Standard deviation of evolvability scores of multiple targets |
| --- | --- | --- |
| Alteplase | Approved | 0.0000 |
| Goserelin | Approved | 0.0556 |
| Reteplase | Approved | 0.0000 |
| Antihemophilic Factor | Approved | 0.0000 |
| Anakinra | Approved | 0.1250 |
| Anistreplase | Approved | 0.0000 |
| Tenecteplase | Approved | 0.0000 |
| Menotropins | Approved | 0.0556 |
| Desmopressin | Approved | 0.0556 |
| Palifermin | Approved | 0.0000 |
| Lutropin alfa | Approved | 0.0556 |
| Collagenase | Approved | 0.0000 |
| Cetrorelix | Approved | 0.0556 |
| Drotrecogin alfa | Approved | 0.0000 |
| Pegademase bovine | Approved | 0.0700 |
| Vasopressin | Approved | 0.0556 |
| Streptokinase | Approved | 0.0556 |
| Felypressin | Approved | 0.0556 |
| Choriogonadotropin alfa | Approved | 0.0556 |
| Octreotide | Approved | 0.0556 |
| Choline | Approved | 0.0000 |
| Adenosine monophosphate | Approved | 0.0786 |
| L-Glutamic Acid | Approved | 0.0556 |
| Phosphatidylserine | Approved | 0.1043 |
| Vitamin E | Approved | 0.1496 |
| Adenosine triphosphate | Approved | 0.0476 |
| Fluvoxamine | Approved | 0.0556 |
| Valsartan | Approved | 0.0556 |
| Amphetamine | Approved | 0.0556 |
| Pentagastrin | Approved | 0.0556 |
| Cevimeline | Approved | 0.0529 |
| Esmolol | Approved | 0.0500 |
| Tramadol | Approved | 0.0556 |
| Betaxolol | Approved | 0.0500 |
| Caffeine | Approved | 0.0471 |
| Succinylcholine | Approved | 0.0529 |
| Trospium | Approved | 0.0529 |
| Midodrine | Approved | 0.0556 |
| Eletriptan | Approved | 0.0529 |
| Bethanidine | Approved | 0.0556 |
| Oxyphenonium | Approved | 0.0529 |
| Isoetharine | Approved | 0.0500 |
| Guanadrel Sulfate | Approved | 0.0556 |
| Benztropine | Approved | 0.0529 |
| Ziprasidone | Approved | 0.0538 |
| Methysergide | Approved | 0.0538 |
| Metoprolol | Approved | 0.0500 |
| Betazole | Approved | 0.0500 |
| Olmesartan | Approved | 0.0556 |
| Theophylline | Approved | 0.0471 |
| Clemastine | Approved | 0.0556 |
| Venlafaxine | Approved | 0.0556 |
| Travoprost | Approved | 0.0556 |
| Bupivacaine | Approved | 0.0556 |
| Dapiprazole | Approved | 0.0556 |
| Zolmitriptan | Approved | 0.0500 |
| Dihydroergotamine | Approved | 0.0556 |
| Ipratropium | Approved | 0.0538 |
| Olanzapine | Approved | 0.0537 |
| Atenolol | Approved | 0.0500 |
| Metixene | Approved | 0.0529 |
| Cetirizine | Approved | 0.0556 |
| Terfenadine | Approved | 0.0556 |
| Alfuzosin | Approved | 0.0556 |
| Methylergonovine | Approved | 0.0500 |
| Buclizine | Approved | 0.0538 |
| Clozapine | Approved | 0.0543 |
| Doxylamine | Approved | 0.0538 |
| Norepinephrine | Approved | 0.0529 |
| Mirtazapine | Approved | 0.0556 |
| Thiethylperazine | Approved | 0.0535 |
| Timolol | Approved | 0.0500 |
| Trihexyphenidyl | Approved | 0.0529 |
| Oxyphencyclimine | Approved | 0.0535 |
| Procyclidine | Approved | 0.0538 |
| Phenylephrine | Approved | 0.0556 |
| Ethopropazine | Approved | 0.0529 |
| Phenylpropanolamine | Approved | 0.0556 |
| Sorafenib | Approved | 0.2042 |
| Ceruletide | Approved | 0.0556 |
| Dexbrompheniramine | Approved | 0.0556 |
| Loxapine | Approved | 0.0500 |
| Carbachol | Approved | 0.0538 |
| Promazine | Approved | 0.0540 |
| Spironolactone | Approved | 0.0556 |
| Hyoscyamine | Approved | 0.0538 |
| Triprolidine | Approved | 0.0556 |
| Carboprost Tromethamine | Approved | 0.0556 |
| Prochlorperazine | Approved | 0.0538 |
| Cyproheptadine | Approved | 0.0556 |
| Framycetin | Approved | 0.0556 |
| Loratadine | Approved | 0.0556 |
| Prazosin | Approved | 0.0556 |
| Methylscopolamine | Approved | 0.0529 |
| Montelukast | Approved | 0.0556 |
| Chlorpromazine | Approved | 0.0556 |
| Gallamine Triethiodide | Approved | 0.0556 |
| Darifenacin | Approved | 0.0529 |
| Cimetidine | Approved | 0.0500 |
| Haloperidol | Approved | 0.0500 |
| Tridihexethyl | Approved | 0.0535 |
| Norgestrel | Approved | 0.0556 |
| Triflupromazine | Approved | 0.0535 |
| Anisotropine Methylbromide | Approved | 0.0535 |
| Carteolol | Approved | 0.0500 |
| Zafirlukast | Approved | 0.0556 |
| Hydroxyzine | Approved | 0.0556 |
| Bosentan | Approved | 0.0556 |
| Cinnarizine | Approved | 0.0556 |
| Propranolol | Approved | 0.0500 |
| Atropine | Approved | 0.0529 |
| Clonidine | Approved | 0.0556 |
| Nizatidine | Approved | 0.0500 |
| Cinalukast | Approved | 0.0556 |
| Lisuride | Approved | 0.0500 |
| Doxazosin | Approved | 0.0556 |
| Labetalol | Approved | 0.0529 |
| Cisapride | Approved | 0.0529 |
| Sulindac | Approved | 0.1735 |
| Metaraminol | Approved | 0.0556 |
| Bisoprolol | Approved | 0.0500 |
| Fluphenazine | Approved | 0.0500 |
| Astemizole | Approved | 0.0556 |
| Adenosine | Approved | 0.0833 |
| Simvastatin | Approved | 0.1768 |
| Dyphylline | Approved | 0.0000 |
| Latanoprost | Approved | 0.0556 |
| Trazodone | Approved | 0.0556 |
| Acamprosate | Approved | 0.0556 |
| Nilutamide | Approved | 0.0556 |
| Histamine Phosphate | Approved | 0.0529 |
| Epinephrine | Approved | 0.0520 |
| Sumatriptan | Approved | 0.0556 |
| Pirenzepine | Approved | 0.0529 |
| Aprepitant | Approved | 0.0556 |
| Losartan | Approved | 0.0556 |
| Thioridazine | Approved | 0.0538 |
| Ergotamine | Approved | 0.0556 |
| Nicergoline | Approved | 0.0556 |
| Tamsulosin | Approved | 0.0556 |
| Apomorphine | Approved | 0.0500 |
| Paroxetine | Approved | 0.0556 |
| Nedocromil | Approved | 0.0556 |
| Azatadine | Approved | 0.0556 |
| Methoxamine | Approved | 0.0556 |
| Homatropine Methylbromide | Approved | 0.0529 |
| Rocuronium | Approved | 0.0556 |
| Diphemanil Methylsulfate | Approved | 0.0529 |
| Risperidone | Approved | 0.0545 |
| Meclizine | Approved | 0.0556 |
| Modafinil | Approved | 0.0556 |
| Scopolamine | Approved | 0.0529 |
| Carbinoxamine | Approved | 0.0538 |
| Epinastine | Approved | 0.0543 |
| Tranylcypromine | Approved | 0.0556 |
| Benzquinamide | Approved | 0.0533 |
| Olopatadine | Approved | 0.0556 |
| Alprostadil | Approved | 0.0556 |
| Clidinium | Approved | 0.0529 |
| Tirofiban | Approved | 0.0000 |
| Propiomazine | Approved | 0.0540 |
| Propantheline | Approved | 0.0529 |
| Cryptenamine | Approved | 0.0529 |
| Tripelennamine | Approved | 0.0556 |
| Candesartan | Approved | 0.0556 |
| Tolazoline | Approved | 0.0556 |
| Fenoldopam | Approved | 0.0500 |
| Dicyclomine | Approved | 0.0529 |
| Minaprine | Approved | 0.0543 |
| Pentoxifylline | Approved | 0.0000 |
| Biperiden | Approved | 0.0529 |
| Orciprenaline | Approved | 0.0500 |
| Enprofylline | Approved | 0.0433 |
| Trifluoperazine | Approved | 0.0556 |
| Brompheniramine | Approved | 0.0556 |
| Dobutamine | Approved | 0.0500 |
| Donepezil | Approved | 0.0556 |
| Perphenazine | Approved | 0.0528 |
| Pseudoephedrine | Approved | 0.0529 |
| Ranitidine | Approved | 0.0500 |
| Benzphetamine | Approved | 0.0556 |
| Alprenolol | Approved | 0.0500 |
| Ritodrine | Approved | 0.0500 |
| Terbutaline | Approved | 0.0500 |
| Conivaptan | Approved | 0.0556 |
| Flupenthixol | Approved | 0.0529 |
| Eprosartan | Approved | 0.0556 |
| Pemirolast | Approved | 0.0556 |
| Granisetron | Approved | 0.0500 |
| Methdilazine | Approved | 0.0556 |
| Ondansetron | Approved | 0.0500 |
| Bimatoprost | Approved | 0.0556 |
| Cocaine | Approved | 0.0500 |
| Amantadine | Approved | 0.0500 |
| Dinoprostone | Approved | 0.0556 |
| Ketotifen | Approved | 0.0556 |
| Cyclobenzaprine | Approved | 0.0556 |
| Phenoxybenzamine | Approved | 0.0524 |
| Famotidine | Approved | 0.0500 |
| Mesoridazine | Approved | 0.0556 |
| Maprotiline | Approved | 0.0556 |
| Oxymetazoline | Approved | 0.0556 |
| Salmeterol | Approved | 0.0500 |
| Methantheline | Approved | 0.0529 |
| Cycrimine | Approved | 0.0529 |
| Fexofenadine | Approved | 0.0556 |
| Rizatriptan | Approved | 0.0500 |
| Pindolol | Approved | 0.0500 |
| Telmisartan | Approved | 0.0556 |
| Desloratadine | Approved | 0.0556 |
| Azelastine | Approved | 0.0556 |
| Cyclopentolate | Approved | 0.0529 |
| Formoterol | Approved | 0.0500 |
| Dimenhydrinate | Approved | 0.0556 |
| Glycopyrrolate | Approved | 0.0529 |
| Dopamine | Approved | 0.0500 |
| Salbutamol | Approved | 0.0471 |
| Guanfacine | Approved | 0.0556 |
| Bethanechol | Approved | 0.0529 |
| Amlexanox | Approved | 0.0556 |
| Irbesartan | Approved | 0.0556 |
| Tolterodine | Approved | 0.0535 |
| Carphenazine | Approved | 0.0500 |
| Oxybutynin | Approved | 0.0535 |
| Acetophenazine | Approved | 0.0500 |
| Isoproterenol | Approved | 0.0776 |
| Promethazine | Approved | 0.0539 |
| Mequitazine | Approved | 0.0556 |
| Diphenhydramine | Approved | 0.0556 |
| Atorvastatin | Approved | 0.1571 |
| Emedastine | Approved | 0.0556 |
| Pilocarpine | Approved | 0.0538 |
| Arbutamine | Approved | 0.0500 |
| Quinacrine | Approved | 0.0499 |
| Levocabastine | Approved | 0.0556 |
| Chlorpheniramine | Approved | 0.0556 |
| Amiodarone | Approved | 0.0529 |
| Doxacurium chloride | Approved | 0.0556 |
| Carvedilol | Approved | 0.0520 |
| Doxepin | Approved | 0.0529 |
| Diphenylpyraline | Approved | 0.0556 |
| Flavoxate | Approved | 0.0538 |
| Nefazodone | Approved | 0.0556 |
| Desipramine | Approved | 0.0529 |
| Bretylium | Approved | 0.0500 |
| Halothane | Approved | 0.0361 |
| Terazosin | Approved | 0.0556 |
| Cilostazol | Approved | 0.0000 |
| Arsenic trioxide | Approved | 0.0393 |
| Guanethidine | Approved | 0.0556 |
| Orphenadrine | Approved | 0.0556 |
| Cyclizine | Approved | 0.0538 |
| Pergolide | Approved | 0.0500 |
| Dexfenfluramine | Approved | 0.0556 |
| Acebutolol | Approved | 0.0500 |
| Nadolol | Approved | 0.0500 |
| Ridogrel | Approved | 0.0556 |
| Levobunolol | Approved | 0.0500 |
| Metipranolol | Approved | 0.0500 |
| Ketamine | Approved | 0.0556 |
| Quetiapine | Approved | 0.0547 |
| Mivacurium | Approved | 0.0556 |
| Diphenidol | Approved | 0.0535 |
| Metoclopramide | Approved | 0.0529 |
| Levodopa | Approved | 0.0500 |
| Bromodiphenhydramine | Approved | 0.0556 |
| Aripiprazole | Approved | 0.0556 |
| Chlorprothixene | Approved | 0.0545 |
| Clomipramine | Approved | 0.0556 |
| Trimeprazine | Approved | 0.0556 |
| Lisdexamfetamine | Approved | 0.0556 |
| Paliperidone | Approved | 0.0556 |
| Arformoterol | Approved | 0.0500 |
| Carbetocin | Approved | 0.0556 |
| Fenoterol | Approved | 0.0500 |
| Pirbuterol | Approved | 0.0500 |
| Bevantolol | Approved | 0.0500 |
| Practolol | Approved | 0.0500 |
| Doxacurium | Approved | 0.0556 |
| Metocurine | Approved | 0.0556 |
| Forasartan | Approved | 0.0556 |
| Saprisartan | Approved | 0.0556 |
| Tasosartan | Approved | 0.0556 |
| Penbutolol | Approved | 0.0500 |
| Mephentermine | Approved | 0.0556 |
| Procaterol | Approved | 0.0433 |
| Methotrimeprazine | Approved | 0.0542 |
| Clenbuterol | Approved | 0.0471 |
| Bambuterol | Approved | 0.0500 |
| Tiotropium | Approved | 0.0529 |
| Pranlukast | Approved | 0.0524 |
| Amrinone | Approved | 0.0000 |
| Dextroamphetamine | Approved | 0.0556 |
| Methamphetamine | Approved | 0.0556 |
| Phendimetrazine | Approved | 0.0556 |
| Oxprenolol | Approved | 0.0500 |
| Solifenacin | Approved | 0.0529 |
| Propericiazine | Approved | 0.0556 |
| Aceprometazine | Approved | 0.0556 |
| Phenindamine | Approved | 0.0556 |
| Pheniramine | Approved | 0.0556 |
| Zuclopenthixol | Approved | 0.0500 |
| Terlipressin | Approved | 0.0556 |
| Maraviroc | Approved | 0.0556 |
| Chlophedianol | Approved | 0.0556 |
| Debrisoquin | Approved | 0.0556 |
| Flunarizine | Approved | 0.0524 |
| Nebivolol | Approved | 0.0500 |
| Bepotastine | Approved | 0.0556 |
| Vapreotide | Approved | 0.0556 |
| Defibrotide | Approved | 0.0500 |
| Sertindole | Approved | 0.0547 |
| Mianserin | Approved | 0.0556 |
| Icatibant | Approved | 0.0556 |
| Droxidopa | Approved | 0.0529 |
| Calcium | Experimental | 0.0556 |
| MMDA | Experimental | 0.0556 |
| 4-Methoxyamphetamine | Experimental | 0.0556 |
| Dimethyltryptamine | Experimental | 0.0500 |
| 4-[3-(Cyclopentyloxy)-4-Methoxyphenyl]-2-Pyrrolidinone | Experimental | 0.0000 |
| Staurosporine | Experimental | 0.0043 |
| Phosphonothreonine | Experimental | 0.2434 |
| Purvalanol | Experimental | 0.0000 |
| Acetic Acid | Experimental | 0.2500 |
| Adenosine-5'-Diphosphate | Experimental | 0.2639 |
| (S)-Rolipram | Experimental | 0.0000 |
| 2-Methyl-2,4-Pentanediol | Experimental | 0.2055 |
| (R)-Rolipram | Experimental | 0.0000 |
| Acetate Ion | Experimental | 0.2357 |
| Guanosine-5'-Diphosphate | Experimental | 0.1059 |
| Arecoline | Experimental | 0.0529 |
| Cacodylate Ion | Experimental | 0.0000 |
| Aniracetam | Experimental | 0.0556 |
