# Supplementary material for: Robustness and Evolvability of the Human Signaling Network
Source: PLoS Comput Biol. 2014 Jul 31;10(7):e1003763. doi: 10.1371/journal.pcbi.1003763 (PMC4117429; doi:10.1371/journal.pcbi.1003763)
Supplement: Table S6 — The normalized proportion of the paths in the evolvable core from perturbed nodes to output nodes over such paths in the original network. The normalized proportion () of the paths in the evolvable core from a node i to a node j is defined as follows: , where , denotes the number of paths in the evolvable core from a node i to a node j, denotes the number of paths in the original network from a node i to a node j, and n denotes the number of all the nodes in the original network. (DOC) [file pcbi.1003763.s024.doc]

**Table S6. The normalized proportion of the paths in the evolvable core from perturbed nodes to output nodes over such paths in the original network. The nomalized proportion ($NP_{ij}$) of the paths in the evolvable core from a node *i* to a node *j* is defined as follows: $NP_{ij}=\frac{P_{ij}}{\max_{1\leq k\leq n} \left(P_{kj}\right)}$, where $P_{ij}=\frac{N_{ij}^{E}}{N_{ij}^{O}}$, $N_{ij}^{E}$ denotes the number of paths in the evolvable core from a node *i* to a node *j*, $N_{ij}^{O}$ denotes the number of paths in the original network from a node *i* to a node *j*, and *n* denotes the number of all the nodes in the original network.**

|  | | Output node | | | |
| --- | --- | --- | --- | --- | --- |
|  |  | Erk | p38 | Akt | SAPK |
| Perturbed node | ASK1 | 1.000 | 0.374 | 1.000 | 0.619 |
|  | Raf | 0.144 | 0.834 | 0.000 | 0.370 |
|  | Actin | 0.064 | 0.606 | 0.000 | 0.213 |
|  | Ras | 0.059 | 0.373 | 0.000 | 0.131 |
|  | Src | 0.039 | 0.344 | 0.000 | 0.121 |
|  | PI3K | 0.037 | 0.313 | 0.000 | 0.113 |
