# Supplementary material for: Robustness and Evolvability of the Human Signaling Network
Source: PLoS Comput Biol. 2014 Jul 31;10(7):e1003763. doi: 10.1371/journal.pcbi.1003763 (PMC4117429; doi:10.1371/journal.pcbi.1003763)
Supplement: Table S7 — List of evolvable core links, robust neighbor links, and redundant links in the context of canalizing function. (DOC) [file pcbi.1003763.s025.doc]

**Table S7. List of evolvable core links, robust neighbor links, and redundant links in the context of canalizing function.**

| Name of source node | Name of target node | Evolvable core/Robust neighbor | Redundancy in the context of canalization |
| --- | --- | --- | --- |
| PKA | PLC_B | Evolvable core | Redundant |
| Gbg_i | PLC_B | Evolvable core | Redundant |
| PLC_B | PLC_B | Evolvable core | Redundant |
| PLC_B | IP3 | Evolvable core | Redundant |
| PLC_g | IP3 | Evolvable core | Redundant |
| ExtPump | Ca | Evolvable core | Redundant |
| IP3R1 | Ca | Evolvable core | Redundant |
| CaM | CaMK | Evolvable core | Redundant |
| CaMKK | CaMK | Evolvable core | Redundant |
| PIP3_345 | RGS | Evolvable core | Redundant |
| CaM | RGS | Evolvable core | Redundant |
| Src | Rap1 | Evolvable core | Redundant |
| PTEN | Shc | Evolvable core | Redundant |
| Shc | Shc | Evolvable core | Redundant |
| Fak | Shc | Evolvable core | Redundant |
| Src | Shc | Evolvable core | Redundant |
| EGFR | Shc | Evolvable core | Redundant |
| Fak | Grb2 | Evolvable core | Redundant |
| Src | Grb2 | Evolvable core | Redundant |
| Shc | Grb2 | Evolvable core | Redundant |
| EGFR | Grb2 | Evolvable core | Redundant |
| Erk | Sos | Evolvable core | Redundant |
| Nck | Sos | Evolvable core | Redundant |
| Crk | Sos | Evolvable core | Redundant |
| Grb2 | Sos | Evolvable core | Redundant |
| SHP2 | Ras | Evolvable core | Redundant |
| RasGRF_GRP | Ras | Evolvable core | Redundant |
| Sos | Ras | Evolvable core | Redundant |
| MKPs | Erk | Evolvable core | Redundant |
| PP2A | Erk | Evolvable core | Redundant |
| Erk | Erk | Evolvable core | Redundant |
| Gbg_i | PI3K | Evolvable core | Redundant |
| Crk | PI3K | Evolvable core | Redundant |
| Fak | PI3K | Evolvable core | Redundant |
| Src | PI3K | Evolvable core | Redundant |
| EGFR | PI3K | Evolvable core | Redundant |
| Ras | PI3K | Evolvable core | Redundant |
| Gab1 | PI3K | Evolvable core | Redundant |
| Src | PDK1 | Evolvable core | Redundant |
| p90RSK | PDK1 | Evolvable core | Redundant |
| PI4K | PIP2_45 | Evolvable core | Redundant |
| PI5K | PIP2_45 | Evolvable core | Redundant |
| PIP3_345 | PIP2_45 | Evolvable core | Redundant |
| PTEN | PIP2_45 | Evolvable core | Redundant |
| Rho | PI4K | Evolvable core | Redundant |
| PKC | PI4K | Evolvable core | Redundant |
| Gai | PI4K | Evolvable core | Redundant |
| Gaq | PI4K | Evolvable core | Redundant |
| ARF | PI4K | Evolvable core | Redundant |
| RhoK | PI5K | Evolvable core | Redundant |
| PA | PI5K | Evolvable core | Redundant |
| ARF | PI5K | Evolvable core | Redundant |
| PI5K | PI5K | Evolvable core | Redundant |
| Talin | PI5K | Evolvable core | Redundant |
| Src | PI5K | Evolvable core | Redundant |
| Fak | PI5K | Evolvable core | Redundant |
| Gbg_s | Gbg_s | Evolvable core | Redundant |
| alpha_s_R | Gbg_s | Evolvable core | Redundant |
| RKIP | GRK | Evolvable core | Redundant |
| Erk | GRK | Evolvable core | Redundant |
| Src | GRK | Evolvable core | Redundant |
| B_Arrestin | GRK | Evolvable core | Redundant |
| PIP2_45 | GRK | Evolvable core | Redundant |
| Gbg_s | GRK | Evolvable core | Redundant |
| Gbg_i | GRK | Evolvable core | Redundant |
| Gbg_q | GRK | Evolvable core | Redundant |
| Gbg_12_13 | GRK | Evolvable core | Redundant |
| Palpha_s_R | B_Arrestin | Evolvable core | Redundant |
| Palpha_q_R | B_Arrestin | Evolvable core | Redundant |
| Palpha_i_R | B_Arrestin | Evolvable core | Redundant |
| Palpha_12_13_R | B_Arrestin | Evolvable core | Redundant |
| ECM | AC | Evolvable core | Redundant |
| Integrins | AC | Evolvable core | Redundant |
| Gbg_i | AC | Evolvable core | Redundant |
| Gas | AC | Evolvable core | Redundant |
| cAMP | cAMP | Evolvable core | Redundant |
| AC | cAMP | Evolvable core | Redundant |
| PP2A | PKA | Evolvable core | Redundant |
| PKA | PKA | Evolvable core | Redundant |
| PDK1 | PKA | Evolvable core | Redundant |
| DAG | RasGRF_GRP | Evolvable core | Redundant |
| CaM | RasGRF_GRP | Evolvable core | Redundant |
| Src | p120RasGAP | Evolvable core | Redundant |
| PKC | RhoGDI | Evolvable core | Redundant |
| PIP2_45 | RhoGDI | Evolvable core | Redundant |
| AA | RhoGDI | Evolvable core | Redundant |
| PIP3_345 | p115RhoGEF | Evolvable core | Redundant |
| Ga_12_13 | p115RhoGEF | Evolvable core | Redundant |
| PTPPEST | Cas | Evolvable core | Redundant |
| Cas | Cas | Evolvable core | Redundant |
| Src | Cas | Evolvable core | Redundant |
| Fak | Cas | Evolvable core | Redundant |
| SHP2 | Csk | Evolvable core | Redundant |
| PKA | Csk | Evolvable core | Redundant |
| Gbg_i | Csk | Evolvable core | Redundant |
| Gbg_q | Csk | Evolvable core | Redundant |
| Gbg_12_13 | Csk | Evolvable core | Redundant |
| Arp_2_3 | Actin | Evolvable core | Redundant |
| Myosin | Actin | Evolvable core | Redundant |
| Vinc | Vinc | Evolvable core | Redundant |
| PTPPEST | Crk | Evolvable core | Redundant |
| Src | Crk | Evolvable core | Redundant |
| Fak | Crk | Evolvable core | Redundant |
| Cas | Crk | Evolvable core | Redundant |
| Cas | Nck | Evolvable core | Redundant |
| PIP3_345 | DOCK180 | Evolvable core | Redundant |
| RhoGDI | Rac | Evolvable core | Redundant |
| p190RhoGAP | Rac | Evolvable core | Redundant |
| RalBP1 | Rac | Evolvable core | Redundant |
| Rac | Rac | Evolvable core | Redundant |
| Integrins | Rac | Evolvable core | Redundant |
| ECM | Rac | Evolvable core | Redundant |
| PAK | Rac | Evolvable core | Redundant |
| Tiam | Rac | Evolvable core | Redundant |
| RasGRF_GRP | Rac | Evolvable core | Redundant |
| B_Arrestin | RalGDS | Evolvable core | Redundant |
| CaM | Ral | Evolvable core | Redundant |
| RhoGDI | Cdc42 | Evolvable core | Redundant |
| Cdc42 | Cdc42 | Evolvable core | Redundant |
| Rac | Cdc42 | Evolvable core | Redundant |
| Src | Cdc42 | Evolvable core | Redundant |
| Gbg_i | Cdc42 | Evolvable core | Redundant |
| PAK | Cdc42 | Evolvable core | Redundant |
| TAK1 | NIK | Evolvable core | Redundant |
| PIP2_34 | Pix_Cool | Evolvable core | Redundant |
| PIP3_345 | Pix_Cool | Evolvable core | Redundant |
| PTP1b | PAK | Evolvable core | Redundant |
| Akt | PAK | Evolvable core | Redundant |
| Nck | PAK | Evolvable core | Redundant |
| Grb2 | PAK | Evolvable core | Redundant |
| Src | PAK | Evolvable core | Redundant |
| PAK | PAK | Evolvable core | Redundant |
| Cdc42 | PAK | Evolvable core | Redundant |
| Rac | PAK | Evolvable core | Redundant |
| Fak | WASP | Evolvable core | Redundant |
| Fak | Graf | Evolvable core | Redundant |
| CaM | MLCK | Evolvable core | Redundant |
| PKA | MLCP | Evolvable core | Redundant |
| SHP2 | Gab1 | Evolvable core | Redundant |
| Grb2 | Gab1 | Evolvable core | Redundant |
| EGFR | Gab1 | Evolvable core | Redundant |
| Gab1 | Gab1 | Evolvable core | Redundant |
| PIP3_345 | Gab1 | Evolvable core | Redundant |
| Trafs | Tab_1_2 | Evolvable core | Redundant |
| Cdc42 | Mekk1 | Evolvable core | Redundant |
| Ras | Mekk1 | Evolvable core | Redundant |
| GCK | Mekk1 | Evolvable core | Redundant |
| Grb2 | Mekk1 | Evolvable core | Redundant |
| Shc | Mekk1 | Evolvable core | Redundant |
| EGFR | Mekk2 | Evolvable core | Redundant |
| IL1_TNFR | Mekk3 | Evolvable core | Redundant |
| Cdc42 | Mekk4 | Evolvable core | Redundant |
| Trafs | Trx | Evolvable core | Redundant |
| Stress | Trx | Evolvable core | Redundant |
| Cdc42 | MLK1 | Evolvable core | Redundant |
| Rac | MLK3 | Evolvable core | Redundant |
| PP2A | p38 | Evolvable core | Redundant |
| p90RSK | p90RSK | Evolvable core | Redundant |
| Erk | p90RSK | Evolvable core | Redundant |
| PDK1 | p90RSK | Evolvable core | Redundant |
| SAPK | MKPs | Evolvable core | Redundant |
| p38 | MKPs | Evolvable core | Redundant |
| Erk | MKPs | Evolvable core | Redundant |
| PKC | PTPPEST | Evolvable core | Redundant |
| PKA | PTPPEST | Evolvable core | Redundant |
| Integrins | PTPPEST | Evolvable core | Redundant |
| ECM | PTPPEST | Evolvable core | Redundant |
| PIP3_345 | ARF | Evolvable core | Redundant |
| PIP2_45 | ARF | Evolvable core | Redundant |
| DAG | DGK | Evolvable core | Redundant |
| PKC | DGK | Evolvable core | Redundant |
| Src | DGK | Evolvable core | Redundant |
| Ca | DGK | Evolvable core | Redundant |
| PA | DGK | Evolvable core | Redundant |
| Gbg_i | Gbg_i | Evolvable core | Redundant |
| alpha_i_R | Gbg_i | Evolvable core | Redundant |
| Gbg_q | Gbg_q | Evolvable core | Redundant |
| alpha_q_R | Gbg_q | Evolvable core | Redundant |
| Gbg_12_13 | Gbg_12_13 | Evolvable core | Redundant |
| alpha_12_13_R | Gbg_12_13 | Evolvable core | Redundant |
| Erk | PDE4 | Evolvable core | Redundant |
| PKA | PDE4 | Evolvable core | Redundant |
| SHP2 | Cbp | Evolvable core | Redundant |
| Src | Cbp | Evolvable core | Redundant |
| GRK | Palpha_s_R | Evolvable core | Redundant |
| alpha_s_R | Palpha_s_R | Evolvable core | Redundant |
| GRK | Palpha_q_R | Evolvable core | Redundant |
| alpha_q_R | Palpha_q_R | Evolvable core | Redundant |
| GRK | Palpha_i_R | Evolvable core | Redundant |
| alpha_i_R | Palpha_i_R | Evolvable core | Redundant |
| GRK | Palpha_12_13_R | Evolvable core | Redundant |
| alpha_12_13_R | Palpha_12_13_R | Evolvable core | Redundant |
| EGF | EGFR | Evolvable core | Non-redundant |
| PKC | EGFR | Evolvable core | Non-redundant |
| Ca | EGFR | Evolvable core | Non-redundant |
| alpha_q_R | EGFR | Evolvable core | Non-redundant |
| alpha_i_R | EGFR | Evolvable core | Non-redundant |
| alpha_12_13_R | EGFR | Evolvable core | Non-redundant |
| Gaq | PLC_B | Evolvable core | Non-redundant |
| PA | PLC_g | Evolvable core | Non-redundant |
| AA | PLC_g | Evolvable core | Non-redundant |
| Src | PLC_g | Evolvable core | Non-redundant |
| Fak | PLC_g | Evolvable core | Non-redundant |
| EGFR | PLC_g | Evolvable core | Non-redundant |
| PIP3_345 | PLC_g | Evolvable core | Non-redundant |
| PIP2_45 | IP3 | Evolvable core | Non-redundant |
| DGK | DAG | Evolvable core | Non-redundant |
| DAG | DAG | Evolvable core | Non-redundant |
| PIP2_45 | DAG | Evolvable core | Non-redundant |
| PLC_B | DAG | Evolvable core | Non-redundant |
| PLC_g | DAG | Evolvable core | Non-redundant |
| Ca | CaM | Evolvable core | Non-redundant |
| CaM | CaMKK | Evolvable core | Non-redundant |
| PIP3_345 | Sos | Evolvable core | Non-redundant |
| PP2A | PKC | Evolvable core | Non-redundant |
| Trx | PKC | Evolvable core | Non-redundant |
| PKC | PKC | Evolvable core | Non-redundant |
| PKC_primed | PKC | Evolvable core | Non-redundant |
| AA | PKC | Evolvable core | Non-redundant |
| DAG | PKC | Evolvable core | Non-redundant |
| Ca | PKC | Evolvable core | Non-redundant |
| PLA2 | AA | Evolvable core | Non-redundant |
| Erk | Raf | Evolvable core | Non-redundant |
| Akt | Raf | Evolvable core | Non-redundant |
| PKA | Raf | Evolvable core | Non-redundant |
| Raf | Raf | Evolvable core | Non-redundant |
| Ras | Raf | Evolvable core | Non-redundant |
| Raf_Loc | Raf | Evolvable core | Non-redundant |
| Src | Raf | Evolvable core | Non-redundant |
| RKIP | Raf | Evolvable core | Non-redundant |
| PAK | Raf | Evolvable core | Non-redundant |
| PP2A | Mek | Evolvable core | Non-redundant |
| Mek | Mek | Evolvable core | Non-redundant |
| Mekk1 | Mek | Evolvable core | Non-redundant |
| Mekk2 | Mek | Evolvable core | Non-redundant |
| Mekk3 | Mek | Evolvable core | Non-redundant |
| Raf | Mek | Evolvable core | Non-redundant |
| Tpl2 | Mek | Evolvable core | Non-redundant |
| Mek | Erk | Evolvable core | Non-redundant |
| PIP2_45 | PLA2 | Evolvable core | Non-redundant |
| PIP3_345 | PLA2 | Evolvable core | Non-redundant |
| Ca | PLA2 | Evolvable core | Non-redundant |
| Erk | PLA2 | Evolvable core | Non-redundant |
| CaMK | PLA2 | Evolvable core | Non-redundant |
| PP2A | Akt | Evolvable core | Non-redundant |
| Akt | Akt | Evolvable core | Non-redundant |
| PIP2_34 | Akt | Evolvable core | Non-redundant |
| PIP3_345 | Akt | Evolvable core | Non-redundant |
| PDK1 | Akt | Evolvable core | Non-redundant |
| CaMKK | Akt | Evolvable core | Non-redundant |
| ILK | Akt | Evolvable core | Non-redundant |
| Src | Akt | Evolvable core | Non-redundant |
| PIP_4 | PIP_4 | Evolvable core | Non-redundant |
| PIP2_45 | PIP2_45 | Evolvable core | Non-redundant |
| PI5K | PIP2_34 | Evolvable core | Non-redundant |
| PTEN | PIP2_34 | Evolvable core | Non-redundant |
| PIP2_34 | PIP2_34 | Evolvable core | Non-redundant |
| PI3K | PIP2_34 | Evolvable core | Non-redundant |
| PI4K | PIP2_34 | Evolvable core | Non-redundant |
| PTEN | PIP3_345 | Evolvable core | Non-redundant |
| PIP3_345 | PIP3_345 | Evolvable core | Non-redundant |
| PI3K | PIP3_345 | Evolvable core | Non-redundant |
| PIP2_45 | PIP3_345 | Evolvable core | Non-redundant |
| PI5K | PIP3_345 | Evolvable core | Non-redundant |
| PIP2_34 | PIP3_345 | Evolvable core | Non-redundant |
| B_Arrestin | alpha_s_R | Evolvable core | Non-redundant |
| Palpha_s_R | alpha_s_R | Evolvable core | Non-redundant |
| alpha_s_R | alpha_s_R | Evolvable core | Non-redundant |
| alpha_s_lig | alpha_s_R | Evolvable core | Non-redundant |
| Gas | Gbg_s | Evolvable core | Non-redundant |
| Gbg_i | Gai | Evolvable core | Non-redundant |
| RGS | Gai | Evolvable core | Non-redundant |
| PKA | Gai | Evolvable core | Non-redundant |
| Gai | Gai | Evolvable core | Non-redundant |
| alpha_s_lig | Gai | Evolvable core | Non-redundant |
| alpha_s_R | Gai | Evolvable core | Non-redundant |
| alpha_i_R | Gai | Evolvable core | Non-redundant |
| Gbg_s | Gas | Evolvable core | Non-redundant |
| PKA | Gas | Evolvable core | Non-redundant |
| RGS | Gas | Evolvable core | Non-redundant |
| Gas | Gas | Evolvable core | Non-redundant |
| alpha_s_R | Gas | Evolvable core | Non-redundant |
| Gbg_q | Gaq | Evolvable core | Non-redundant |
| RGS | Gaq | Evolvable core | Non-redundant |
| PLC_B | Gaq | Evolvable core | Non-redundant |
| Gaq | Gaq | Evolvable core | Non-redundant |
| alpha_q_R | Gaq | Evolvable core | Non-redundant |
| PDE4 | cAMP | Evolvable core | Non-redundant |
| cAMP | PKA | Evolvable core | Non-redundant |
| Cdc42 | RasGRF_GRP | Evolvable core | Non-redundant |
| PKA | Rho | Evolvable core | Non-redundant |
| p190RhoGAP | Rho | Evolvable core | Non-redundant |
| RhoGDI | Rho | Evolvable core | Non-redundant |
| Rho | Rho | Evolvable core | Non-redundant |
| p115RhoGEF | Rho | Evolvable core | Non-redundant |
| Src | p190RhoGAP | Evolvable core | Non-redundant |
| Rho | RhoK | Evolvable core | Non-redundant |
| PTEN | Fak | Evolvable core | Non-redundant |
| Src | Fak | Evolvable core | Non-redundant |
| Fak | Fak | Evolvable core | Non-redundant |
| Talin | Fak | Evolvable core | Non-redundant |
| Integrins | Fak | Evolvable core | Non-redundant |
| Csk | Src | Evolvable core | Non-redundant |
| Src | Src | Evolvable core | Non-redundant |
| B_Arrestin | Src | Evolvable core | Non-redundant |
| Gai | Src | Evolvable core | Non-redundant |
| Gas | Src | Evolvable core | Non-redundant |
| alpha_s_R | Src | Evolvable core | Non-redundant |
| Fak | Src | Evolvable core | Non-redundant |
| PTP1b | Src | Evolvable core | Non-redundant |
| Cas | Src | Evolvable core | Non-redundant |
| PTPa | Src | Evolvable core | Non-redundant |
| EGFR | Src | Evolvable core | Non-redundant |
| Cbp | Csk | Evolvable core | Non-redundant |
| PAK | Myosin | Evolvable core | Non-redundant |
| ILK | Integrins | Evolvable core | Non-redundant |
| Src | Integrins | Evolvable core | Non-redundant |
| Integrins | Integrins | Evolvable core | Non-redundant |
| PP2A | Integrins | Evolvable core | Non-redundant |
| ECM | Integrins | Evolvable core | Non-redundant |
| Talin | Integrins | Evolvable core | Non-redundant |
| PKC | RKIP | Evolvable core | Non-redundant |
| Talin | Vinc | Evolvable core | Non-redundant |
| PIP2_34 | Tiam | Evolvable core | Non-redundant |
| Cas | AND_3_4 | Evolvable core | Non-redundant |
| Ral | RalBP1 | Evolvable core | Non-redundant |
| Pix_Cool | Cdc42 | Evolvable core | Non-redundant |
| Src | Talin | Evolvable core | Non-redundant |
| Talin | Talin | Evolvable core | Non-redundant |
| PIP2_45 | Talin | Evolvable core | Non-redundant |
| PIP3_345 | ILK | Evolvable core | Non-redundant |
| WASP | Arp_2_3 | Evolvable core | Non-redundant |
| B_Parvin | Pix_Cool | Evolvable core | Non-redundant |
| PKA | PAK | Evolvable core | Non-redundant |
| IL1_TNF | IL1_TNFR | Evolvable core | Non-redundant |
| IL1_TNFR | Trafs | Evolvable core | Non-redundant |
| Trafs | GCK | Evolvable core | Non-redundant |
| Trx | ASK1 | Evolvable core | Non-redundant |
| Tab_1_2 | TAK1 | Evolvable core | Non-redundant |
| Trafs | Tpl2 | Evolvable core | Non-redundant |
| SAPK | MLK2 | Evolvable core | Non-redundant |
| Stress | TAO_1_2 | Evolvable core | Non-redundant |
| ASK1 | Sek1 | Evolvable core | Non-redundant |
| ASK1 | MKK7 | Evolvable core | Non-redundant |
| ASK1 | MKK3 | Evolvable core | Non-redundant |
| ASK1 | MKK6 | Evolvable core | Non-redundant |
| SAPK | SAPK | Evolvable core | Non-redundant |
| MKK7 | SAPK | Evolvable core | Non-redundant |
| Sek1 | SAPK | Evolvable core | Non-redundant |
| EGFR | PP2A | Evolvable core | Non-redundant |
| Gab1 | SHP2 | Evolvable core | Non-redundant |
| cAMP | MKPs | Evolvable core | Non-redundant |
| Src | PTEN | Evolvable core | Non-redundant |
| PTEN | PTEN | Evolvable core | Non-redundant |
| Stress | PTEN | Evolvable core | Non-redundant |
| Rho | PTEN | Evolvable core | Non-redundant |
| PI3K | PTEN | Evolvable core | Non-redundant |
| Cdc42 | PTEN | Evolvable core | Non-redundant |
| Pix_Cool | PTEN | Evolvable core | Non-redundant |
| Stress | PTP1b | Evolvable core | Non-redundant |
| PKC | PTPa | Evolvable core | Non-redundant |
| Actin | PLD | Evolvable core | Non-redundant |
| PIP3_345 | PLD | Evolvable core | Non-redundant |
| PIP2_45 | PLD | Evolvable core | Non-redundant |
| ARF | PLD | Evolvable core | Non-redundant |
| PKC | PLD | Evolvable core | Non-redundant |
| PLD | PA | Evolvable core | Non-redundant |
| EGFR | DGK | Evolvable core | Non-redundant |
| ILK | B_Parvin | Evolvable core | Non-redundant |
| B_Arrestin | alpha_i_R | Evolvable core | Non-redundant |
| Palpha_i_R | alpha_i_R | Evolvable core | Non-redundant |
| alpha_i_R | alpha_i_R | Evolvable core | Non-redundant |
| alpha_i_lig | alpha_i_R | Evolvable core | Non-redundant |
| B_Arrestin | alpha_q_R | Evolvable core | Non-redundant |
| Palpha_i_R | alpha_q_R | Evolvable core | Non-redundant |
| alpha_q_R | alpha_q_R | Evolvable core | Non-redundant |
| alpha_q_lig | alpha_q_R | Evolvable core | Non-redundant |
| B_Arrestin | alpha_12_13_R | Evolvable core | Non-redundant |
| Palpha_12_13_R | alpha_12_13_R | Evolvable core | Non-redundant |
| alpha_12_13_R | alpha_12_13_R | Evolvable core | Non-redundant |
| alpha_12_13_lig | alpha_12_13_R | Evolvable core | Non-redundant |
| Gbg_12_13 | Ga_12_13 | Evolvable core | Non-redundant |
| p115RhoGEF | Ga_12_13 | Evolvable core | Non-redundant |
| Ga_12_13 | Ga_12_13 | Evolvable core | Non-redundant |
| alpha_12_13_R | Ga_12_13 | Evolvable core | Non-redundant |
| Gai | Gbg_i | Evolvable core | Non-redundant |
| Gaq | Gbg_q | Evolvable core | Non-redundant |
| Ga_12_13 | Gbg_12_13 | Evolvable core | Non-redundant |
| B_Arrestin | PDE4 | Evolvable core | Non-redundant |
| PP2A | IP3R1 | Evolvable core | Non-redundant |
| CaM | IP3R1 | Evolvable core | Non-redundant |
| Ca | IP3R1 | Evolvable core | Non-redundant |
| IP3R1 | IP3R1 | Evolvable core | Non-redundant |
| IP3 | IP3R1 | Evolvable core | Non-redundant |
| Gbg_i | IP3R1 | Evolvable core | Non-redundant |
| PKA | IP3R1 | Evolvable core | Non-redundant |
| Raf_Loc | Raf_DeP | Evolvable core | Non-redundant |
| Raf_DeP | Raf_DeP | Evolvable core | Non-redundant |
| Raf_Rest | Raf_DeP | Evolvable core | Non-redundant |
| PP2A | Raf_DeP | Evolvable core | Non-redundant |
| PKC | PKC_primed | Evolvable core | Non-redundant |
| PKC_primed | PKC_primed | Evolvable core | Non-redundant |
| PDK1 | PKC_primed | Evolvable core | Non-redundant |
| Raf | Raf_Loc | Evolvable core | Non-redundant |
| Raf_Loc | Raf_Loc | Evolvable core | Non-redundant |
| Raf_DeP | Raf_Loc | Evolvable core | Non-redundant |
| Ras | Raf_Loc | Evolvable core | Non-redundant |
| Raf_DeP | Raf_Rest | Evolvable core | Non-redundant |
| PKA | CaMKK | Robust neighbor | Redundant |
| PP2A | CaMK | Robust neighbor | Redundant |
| Gai | Rap1 | Robust neighbor | Redundant |
| Rap1 | Rap1 | Robust neighbor | Redundant |
| cAMP | Rap1 | Robust neighbor | Redundant |
| PKA | Rap1 | Robust neighbor | Redundant |
| CaMK | Rap1 | Robust neighbor | Redundant |
| PIP2_45 | Sos | Robust neighbor | Redundant |
| Ras | Sos | Robust neighbor | Redundant |
| p120RasGAP | Ras | Robust neighbor | Redundant |
| PI3K | PIP2_45 | Robust neighbor | Redundant |
| PLC_B | PIP2_45 | Robust neighbor | Redundant |
| PLC_g | PIP2_45 | Robust neighbor | Redundant |
| Rho | PI5K | Robust neighbor | Redundant |
| Rac | PI5K | Robust neighbor | Redundant |
| PKC | GRK | Robust neighbor | Redundant |
| PKC | AC | Robust neighbor | Redundant |
| Fak | p120RasGAP | Robust neighbor | Redundant |
| SHP2 | p120RasGAP | Robust neighbor | Redundant |
| Ca | p120RasGAP | Robust neighbor | Redundant |
| PIP2_45 | p120RasGAP | Robust neighbor | Redundant |
| PIP2_34 | p120RasGAP | Robust neighbor | Redundant |
| PIP3_345 | p120RasGAP | Robust neighbor | Redundant |
| EGFR | p120RasGAP | Robust neighbor | Redundant |
| p120RasGAP | p190RhoGAP | Robust neighbor | Redundant |
| p190RhoGAP | p190RhoGAP | Robust neighbor | Redundant |
| Fak | p190RhoGAP | Robust neighbor | Redundant |
| Src | Csk | Robust neighbor | Redundant |
| Fak | Csk | Robust neighbor | Redundant |
| PIP2_45 | Vinc | Robust neighbor | Redundant |
| Src | Vinc | Robust neighbor | Redundant |
| Actin | Vinc | Robust neighbor | Redundant |
| EGFR | Nck | Robust neighbor | Redundant |
| Crk | DOCK180 | Robust neighbor | Redundant |
| Cas | DOCK180 | Robust neighbor | Redundant |
| Akt | Rac | Robust neighbor | Redundant |
| Gbg_i | Rac | Robust neighbor | Redundant |
| Cdc42 | Rac | Robust neighbor | Redundant |
| Pix_Cool | Rac | Robust neighbor | Redundant |
| DOCK180 | Rac | Robust neighbor | Redundant |
| PKC | RalGDS | Robust neighbor | Redundant |
| PDK1 | RalGDS | Robust neighbor | Redundant |
| Ras | RalGDS | Robust neighbor | Redundant |
| PIP3_345 | RalGDS | Robust neighbor | Redundant |
| alpha_12_13_R | RalGDS | Robust neighbor | Redundant |
| alpha_s_R | RalGDS | Robust neighbor | Redundant |
| alpha_q_R | RalGDS | Robust neighbor | Redundant |
| alpha_i_R | RalGDS | Robust neighbor | Redundant |
| AND_3_4 | Ral | Robust neighbor | Redundant |
| RalGDS | Ral | Robust neighbor | Redundant |
| RalBP1 | Cdc42 | Robust neighbor | Redundant |
| p190RhoGAP | Cdc42 | Robust neighbor | Redundant |
| Graf | Cdc42 | Robust neighbor | Redundant |
| Nck | NIK | Robust neighbor | Redundant |
| Tpl2 | NIK | Robust neighbor | Redundant |
| PDK1 | PAK | Robust neighbor | Redundant |
| PTPPEST | WASP | Robust neighbor | Redundant |
| Cdc42 | WASP | Robust neighbor | Redundant |
| Crk | WASP | Robust neighbor | Redundant |
| Src | WASP | Robust neighbor | Redundant |
| Nck | WASP | Robust neighbor | Redundant |
| Grb2 | WASP | Robust neighbor | Redundant |
| PIP2_45 | WASP | Robust neighbor | Redundant |
| Src | Graf | Robust neighbor | Redundant |
| PAK | MLCK | Robust neighbor | Redundant |
| PKA | MLCK | Robust neighbor | Redundant |
| Erk | MLCK | Robust neighbor | Redundant |
| RhoK | MLCP | Robust neighbor | Redundant |
| PKC | MLCP | Robust neighbor | Redundant |
| ILK | MLCP | Robust neighbor | Redundant |
| PAK | MLCP | Robust neighbor | Redundant |
| Raf | MLCP | Robust neighbor | Redundant |
| p38 | Tab_1_2 | Robust neighbor | Redundant |
| NIK | Mekk1 | Robust neighbor | Redundant |
| Rho | Mekk1 | Robust neighbor | Redundant |
| Rac | Mekk1 | Robust neighbor | Redundant |
| Trafs | Mekk1 | Robust neighbor | Redundant |
| Mekk2 | Mekk2 | Robust neighbor | Redundant |
| Src | Mekk2 | Robust neighbor | Redundant |
| PI3K | Mekk2 | Robust neighbor | Redundant |
| Grb2 | Mekk2 | Robust neighbor | Redundant |
| PLC_g | Mekk2 | Robust neighbor | Redundant |
| Trafs | Mekk3 | Robust neighbor | Redundant |
| Rac | Mekk3 | Robust neighbor | Redundant |
| Rac | Mekk4 | Robust neighbor | Redundant |
| Akt | ASK1 | Robust neighbor | Redundant |
| Rac | MLK1 | Robust neighbor | Redundant |
| Cdc42 | MLK2 | Robust neighbor | Redundant |
| Rac | MLK2 | Robust neighbor | Redundant |
| IL1_TNFR | MLK3 | Robust neighbor | Redundant |
| Cdc42 | MLK3 | Robust neighbor | Redundant |
| Tpl2 | Sek1 | Robust neighbor | Redundant |
| TAK1 | Sek1 | Robust neighbor | Redundant |
| MLK3 | Sek1 | Robust neighbor | Redundant |
| MLK2 | Sek1 | Robust neighbor | Redundant |
| MLK1 | Sek1 | Robust neighbor | Redundant |
| Mekk4 | Sek1 | Robust neighbor | Redundant |
| Mekk3 | Sek1 | Robust neighbor | Redundant |
| Mekk2 | Sek1 | Robust neighbor | Redundant |
| Mekk1 | Sek1 | Robust neighbor | Redundant |
| MLK3 | MKK7 | Robust neighbor | Redundant |
| MLK2 | MKK7 | Robust neighbor | Redundant |
| MLK1 | MKK7 | Robust neighbor | Redundant |
| Mekk4 | MKK7 | Robust neighbor | Redundant |
| Mekk3 | MKK7 | Robust neighbor | Redundant |
| Mekk2 | MKK7 | Robust neighbor | Redundant |
| Mekk1 | MKK7 | Robust neighbor | Redundant |
| PAK | MKK3 | Robust neighbor | Redundant |
| TAO_1_2 | MKK3 | Robust neighbor | Redundant |
| Tpl2 | MKK3 | Robust neighbor | Redundant |
| TAK1 | MKK3 | Robust neighbor | Redundant |
| MLK3 | MKK3 | Robust neighbor | Redundant |
| MLK2 | MKK3 | Robust neighbor | Redundant |
| MLK1 | MKK3 | Robust neighbor | Redundant |
| Mekk4 | MKK3 | Robust neighbor | Redundant |
| Mekk3 | MKK3 | Robust neighbor | Redundant |
| Mekk2 | MKK3 | Robust neighbor | Redundant |
| PAK | MKK6 | Robust neighbor | Redundant |
| TAO_1_2 | MKK6 | Robust neighbor | Redundant |
| Tpl2 | MKK6 | Robust neighbor | Redundant |
| TAK1 | MKK6 | Robust neighbor | Redundant |
| Mekk4 | MKK6 | Robust neighbor | Redundant |
| MLK3 | MKK6 | Robust neighbor | Redundant |
| MKPs | p38 | Robust neighbor | Redundant |
| p38 | p38 | Robust neighbor | Redundant |
| Sek1 | p38 | Robust neighbor | Redundant |
| MKK6 | p38 | Robust neighbor | Redundant |
| MKK3 | p38 | Robust neighbor | Redundant |
| Src | PP2A | Robust neighbor | Redundant |
| PP2A | PP2A | Robust neighbor | Redundant |
| cAMP | PP2A | Robust neighbor | Redundant |
| EGF | PTP1b | Robust neighbor | Redundant |
| EGFR | PTP1b | Robust neighbor | Redundant |
| EGFR | EGFR | Robust neighbor | Non-redundant |
| PTP1b | EGFR | Robust neighbor | Non-redundant |
| PAK | Mek | Robust neighbor | Non-redundant |
| PI3K | PIP_4 | Robust neighbor | Non-redundant |
| PI5K | PIP_4 | Robust neighbor | Non-redundant |
| PIP2_34 | PIP_4 | Robust neighbor | Non-redundant |
| PTEN | PIP_4 | Robust neighbor | Non-redundant |
| PI4K | PIP_4 | Robust neighbor | Non-redundant |
| Graf | Rho | Robust neighbor | Non-redundant |
| PKA | Src | Robust neighbor | Non-redundant |
| MLCP | Myosin | Robust neighbor | Non-redundant |
| Myosin | Myosin | Robust neighbor | Non-redundant |
| RhoK | Myosin | Robust neighbor | Non-redundant |
| ILK | Myosin | Robust neighbor | Non-redundant |
| MLCK | Myosin | Robust neighbor | Non-redundant |
| CaM | Myosin | Robust neighbor | Non-redundant |
| Rap1 | Tiam | Robust neighbor | Non-redundant |
| Ras | Tiam | Robust neighbor | Non-redundant |
| PIP2_45 | Tiam | Robust neighbor | Non-redundant |
| PIP3_345 | Tiam | Robust neighbor | Non-redundant |
| CaMK | Tiam | Robust neighbor | Non-redundant |
| Src | Tiam | Robust neighbor | Non-redundant |
| PKC | Tiam | Robust neighbor | Non-redundant |
| PKC | Talin | Robust neighbor | Non-redundant |
| Gab1 | Mekk3 | Robust neighbor | Non-redundant |
| MKPs | SAPK | Robust neighbor | Non-redundant |
| PP2A | SAPK | Robust neighbor | Non-redundant |
| Ral | PLD | Robust neighbor | Non-redundant |
| Rac | PLD | Robust neighbor | Non-redundant |
| Rho | PLD | Robust neighbor | Non-redundant |
| Cdc42 | PLD | Robust neighbor | Non-redundant |
| PKC | IP3R1 | Robust neighbor | Non-redundant |
| Raf_Rest | Raf_Rest | Robust neighbor | Non-redundant |
| Raf | Raf_Rest | Robust neighbor | Non-redundant |
