# Supplementary material for: Robustness and Evolvability of the Human Signaling Network
Source: PLoS Comput Biol. 2014 Jul 31;10(7):e1003763. doi: 10.1371/journal.pcbi.1003763 (PMC4117429; doi:10.1371/journal.pcbi.1003763)
Supplement: Table S18 — Primary attractor states of the human signaling network obtained from the simulation with asynchronous update of Boolean functions. (DOC) [file pcbi.1003763.s036.doc]

**Table S18**. Primary attractor states of the human signaling network obtained from the simulation with asynchronous update of Boolean functions.

| Node name | Primary attractor | |
| --- | --- | --- |
| EGFR | 0 | 0 |
| PLC_B | 0 | 0 |
| PLC_g | 0 | 0 |
| IP3 | 0 | 0 |
| DAG | 0 | 0 |
| Ca | 0 | 0 |
| CaM | 0 | 0 |
| CaMKK | 0 | 0 |
| CaMK | 0 | 0 |
| RGS | 0 | 0 |
| Rap1 | 0 | 0 |
| Shc | 0 | 0 |
| Grb2 | 0 | 0 |
| Sos | 0 | 0 |
| Ras | 0 | 0 |
| PKC | 0 | 0 |
| AA | 0 | 0 |
| Raf | 0 | 0 |
| Mek | 0 | 0 |
| Erk | 0 | 0 |
| PLA2 | 0 | 0 |
| PI3K | 0 | 0 |
| Akt | 0 | 0 |
| PDK1 | 0 | 0 |
| PIP_4 | 0 | 1 |
| PIP2_45 | 1 | 1 |
| PIP2_34 | 0 | 0 |
| PIP3_345 | 0 | 0 |
| PI4K | 1 | 1 |
| PI5K | 1 | 1 |
| α_s_R | 0 | 0 |
| Gbg_s | 0 | 0 |
| Gai | 0 | 0 |
| Gas | 0 | 0 |
| Gaq | 0 | 0 |
| GRK | 0 | 0 |
| B_Arrestin | 0 | 0 |
| AC | 0 | 0 |
| cAMP | 0 | 0 |
| PKA | 0 | 0 |
| RasGRF_GRP | 0 | 0 |
| p120RasGAP | 1 | 1 |
| Rho | 0 | 0 |
| RhoGDI | 0 | 0 |
| p190RhoGAP | 0 | 0 |
| p115RhoGEF | 0 | 0 |
| RhoK | 0 | 0 |
| Fak | 0 | 0 |
| Cas | 0 | 0 |
| Src | 0 | 0 |
| Csk | 0 | 0 |
| Myosin | 0 | 0 |
| Actin | 0 | 0 |
| Integrins | 0 | 0 |
| RKIP | 0 | 0 |
| Vinc | 0 | 0 |
| Crk | 0 | 0 |
| Nck | 0 | 0 |
| DOCK180 | 0 | 0 |
| Rac | 0 | 0 |
| Tiam | 0 | 0 |
| RalGDS | 0 | 0 |
| AND_3_4 | 0 | 0 |
| Ral | 0 | 0 |
| RalBP1 | 0 | 0 |
| Cdc42 | 0 | 0 |
| NIK | 0 | 0 |
| Talin | 1 | 1 |
| ILK | 0 | 0 |
| Arp_2_3 | 0 | 0 |
| Pix_Cool | 0 | 0 |
| PAK | 0 | 0 |
| WASP | 0 | 0 |
| Graf | 0 | 0 |
| MLCK | 0 | 0 |
| MLCP | 1 | 1 |
| IL1_TNFR | 0 | 0 |
| Trafs | 0 | 0 |
| GCK | 0 | 0 |
| Gab1 | 0 | 0 |
| Tab_1_2 | 0 | 0 |
| Mekk1 | 0 | 0 |
| Mekk2 | 0 | 0 |
| Mekk3 | 0 | 0 |
| Mekk4 | 0 | 0 |
| ASK1 | 0 | 0 |
| Trx | 0 | 0 |
| TAK1 | 0 | 0 |
| Tpl2 | 0 | 0 |
| MLK1 | 0 | 0 |
| MLK2 | 0 | 0 |
| MLK3 | 0 | 0 |
| TAO_1_2 | 0 | 0 |
| Sek1 | 0 | 0 |
| MKK7 | 0 | 0 |
| MKK3 | 0 | 0 |
| MKK6 | 0 | 0 |
| SAPK | 0 | 0 |
| p38 | 0 | 0 |
| p90RSK | 0 | 0 |
| PP2A | 1 | 1 |
| SHP2 | 0 | 0 |
| MKPs | 0 | 0 |
| PTEN | 0 | 0 |
| PTP1b | 1 | 1 |
| PTPPEST | 0 | 0 |
| PTPa | 0 | 0 |
| PLD | 1 | 1 |
| PA | 1 | 1 |
| ARF | 1 | 1 |
| DGK | 0 | 0 |
| B_Parvin | 0 | 0 |
| α_i_R | 0 | 0 |
| α_q_R | 0 | 0 |
| α_12_13_R | 0 | 0 |
| Ga_12_13 | 0 | 0 |
| Gbg_i | 0 | 0 |
| Gbg_q | 0 | 0 |
| Gbg_12_13 | 0 | 0 |
| PDE4 | 0 | 0 |
| Cbp | 0 | 0 |
| IP3R1 | 0 | 0 |
| Raf_DeP | 1 | 0 |
| PKC_primed | 1 | 1 |
| Raf_Loc | 1 | 1 |
| Raf_Rest | 0 | 1 |
| Pα_s_R | 0 | 0 |
| Pα_q_R | 0 | 0 |
| Pα_i_R | 0 | 0 |
| Pα_12_13_R | 0 | 0 |
| EGF | 0 | 0 |
| ECM | 0 | 0 |
| α_q_lig | 0 | 0 |
| α_i_lig | 0 | 0 |
| α_s_lig | 0 | 0 |
| α_12_13_lig | 0 | 0 |
| Stress | 0 | 0 |
| IL1_TNF | 0 | 0 |
| ExtPump | 0 | 0 |
