# Supplementary material for: Robustness and Evolvability of the Human Signaling Network
Source: PLoS Comput Biol. 2014 Jul 31;10(7):e1003763. doi: 10.1371/journal.pcbi.1003763 (PMC4117429; doi:10.1371/journal.pcbi.1003763)
Supplement: Table S19 — The emergent function of information processing in the evolvable core of the human signaling network. We followed the same procedure proposed by Helikar et al. [22]. This table shows that the relatively small number of output categories are observed from 10,000 simulations with different inputs, where the input values denotes a percentage ‘ON’ for the input node in the Boolean iteration of 1,000 times and the output values denote the average number of ‘ON’s over the last 100 iterations out of the Boolean iteration of 1,000 times. The output values were categorized by using three different ranges; 0 (0∼9%), 1 (10∼29%), and 2 (30∼100%). A four-tuple of numbers in the legends represents a category of four output nodes (‘Akt’, ‘Erk’, ‘Rac’, and ‘Cdc42’). (DOC) [file pcbi.1003763.s037.doc]

**Table S19. The emergent function of information processing in the evolvable core of the human signaling network. We followed the same procedure proposed by Helikar *et al*. [22]. This table shows that the relatively small number of output categories are observed from 10,000 simulations with different inputs, where the input values denotes a percentage ‘ON’ for the input node in the Boolean iteration of 1,000 times and the output values denote the average number of ‘ON’s over the last 100 iterations out of the Boolean iteration of 1,000 times. The output values were categorized by using three different ranges; 0 (0~9%), 1 (10~29%), and 2 (30~100%). A four-tuple of numbers in the legends represents a category of four output nodes (‘Akt’, ‘Erk’, ‘Rac’, and ‘Cdc42’).**

| Output category | Count | Average input | | | | | | | | | Average output | | | |
| --- | --- | --- | --- | --- | --- | --- | --- | --- | --- | --- | --- | --- | --- | --- |
|  |  | EGF | ECM | α_q_lig | α_i_lig | α_s_lig | α_12_13_lig | Stress | IL1_TNF | ExtPump | Akt | Erk | Cdc42 | Rac |
| 2000 | 1465 | 64 | 37 | 49 | 51 | 50 | 51 | 2 | 1 | 44 | 54 | 3 | 2 | 2 |
| 2100 | 1068 | 65 | 36 | 49 | 53 | 50 | 51 | 2 | 3 | 43 | 55 | 17 | 2 | 2 |
| 1000 | 852 | 18 | 28 | 49 | 49 | 48 | 53 | 2 | 2 | 47 | 19 | 3 | 2 | 3 |
| 2110 | 664 | 65 | 54 | 49 | 53 | 50 | 56 | 2 | 2 | 40 | 53 | 18 | 18 | 5 |
| 2200 | 660 | 74 | 36 | 53 | 45 | 51 | 50 | 2 | 3 | 61 | 67 | 50 | 1 | 1 |
| 2120 | 474 | 71 | 54 | 52 | 52 | 49 | 39 | 2 | 2 | 61 | 60 | 19 | 52 | 5 |
| 2220 | 418 | 78 | 54 | 50 | 50 | 45 | 35 | 2 | 3 | 58 | 68 | 46 | 59 | 4 |
| 2010 | 408 | 56 | 49 | 51 | 54 | 49 | 59 | 2 | 1 | 54 | 48 | 4 | 18 | 5 |
| 1011 | 386 | 16 | 69 | 45 | 51 | 50 | 56 | 2 | 2 | 51 | 19 | 4 | 19 | 15 |
| 2121 | 375 | 49 | 80 | 51 | 60 | 51 | 41 | 2 | 2 | 52 | 46 | 19 | 52 | 16 |
| 2020 | 313 | 65 | 49 | 50 | 49 | 50 | 47 | 2 | 1 | 72 | 59 | 4 | 52 | 5 |
| 1010 | 291 | 21 | 37 | 48 | 52 | 50 | 53 | 2 | 2 | 56 | 20 | 4 | 16 | 6 |
| 2210 | 266 | 73 | 55 | 51 | 44 | 45 | 54 | 2 | 3 | 53 | 62 | 43 | 19 | 4 |
| 1021 | 223 | 17 | 70 | 46 | 54 | 51 | 39 | 2 | 2 | 66 | 19 | 4 | 46 | 19 |
| 2111 | 218 | 51 | 81 | 51 | 60 | 50 | 57 | 2 | 2 | 33 | 44 | 18 | 20 | 14 |
| 1100 | 210 | 24 | 31 | 54 | 35 | 54 | 51 | 2 | 3 | 42 | 22 | 15 | 2 | 3 |
| 0000 | 190 | 14 | 31 | 48 | 26 | 46 | 51 | 2 | 2 | 61 | 6 | 2 | 2 | 3 |
| 2021 | 180 | 43 | 77 | 49 | 57 | 51 | 44 | 2 | 1 | 68 | 42 | 4 | 50 | 16 |
| 2011 | 155 | 41 | 78 | 50 | 57 | 51 | 60 | 2 | 2 | 45 | 40 | 5 | 19 | 13 |
| 1001 | 154 | 14 | 73 | 49 | 36 | 52 | 53 | 2 | 2 | 36 | 18 | 3 | 4 | 14 |
| 2221 | 154 | 55 | 81 | 53 | 57 | 53 | 31 | 2 | 2 | 45 | 52 | 41 | 64 | 15 |
| 1121 | 106 | 22 | 75 | 53 | 52 | 50 | 36 | 2 | 2 | 61 | 23 | 15 | 52 | 19 |
| 1111 | 95 | 23 | 77 | 52 | 45 | 52 | 58 | 2 | 3 | 42 | 22 | 14 | 19 | 16 |
| 2001 | 84 | 47 | 78 | 48 | 48 | 53 | 56 | 2 | 1 | 31 | 42 | 4 | 5 | 13 |
| 1110 | 81 | 28 | 40 | 42 | 46 | 45 | 43 | 2 | 3 | 36 | 24 | 15 | 17 | 5 |
| 2101 | 75 | 53 | 81 | 57 | 43 | 55 | 57 | 2 | 3 | 27 | 44 | 17 | 5 | 13 |
| 0011 | 63 | 8 | 64 | 49 | 31 | 45 | 52 | 2 | 2 | 72 | 7 | 2 | 17 | 15 |
| 0001 | 58 | 9 | 72 | 57 | 18 | 51 | 46 | 2 | 2 | 47 | 6 | 2 | 3 | 15 |
| 1020 | 55 | 23 | 32 | 58 | 56 | 47 | 32 | 2 | 2 | 64 | 20 | 4 | 47 | 5 |
| 2211 | 44 | 63 | 86 | 49 | 41 | 47 | 59 | 2 | 3 | 42 | 49 | 38 | 19 | 13 |
| 1022 | 41 | 13 | 91 | 46 | 57 | 51 | 46 | 2 | 2 | 75 | 18 | 4 | 57 | 33 |
| 1101 | 34 | 24 | 81 | 55 | 23 | 59 | 56 | 3 | 3 | 40 | 21 | 15 | 5 | 15 |
| 0021 | 30 | 10 | 68 | 54 | 31 | 47 | 38 | 2 | 2 | 69 | 8 | 2 | 45 | 20 |
| 0010 | 28 | 16 | 38 | 61 | 24 | 41 | 36 | 2 | 2 | 58 | 7 | 4 | 16 | 6 |
| 2201 | 16 | 53 | 84 | 68 | 36 | 51 | 57 | 2 | 3 | 30 | 46 | 35 | 4 | 12 |
| 1120 | 15 | 32 | 35 | 47 | 40 | 51 | 22 | 2 | 2 | 65 | 24 | 15 | 46 | 5 |
| 1122 | 11 | 14 | 95 | 49 | 59 | 32 | 22 | 2 | 2 | 69 | 21 | 16 | 72 | 33 |
| 0100 | 5 | 18 | 42 | 34 | 18 | 45 | 52 | 1 | 3 | 18 | 9 | 14 | 6 | 6 |
| 0020 | 4 | 6 | 24 | 61 | 14 | 38 | 9 | 2 | 1 | 68 | 8 | 1 | 41 | 4 |
| 1012 | 4 | 8 | 88 | 32 | 50 | 38 | 37 | 4 | 1 | 67 | 13 | 1 | 21 | 34 |
| 1201 | 4 | 42 | 92 | 33 | 5 | 41 | 74 | 1 | 4 | 60 | 24 | 35 | 2 | 12 |
| 0012 | 3 | 3 | 95 | 58 | 40 | 63 | 57 | 3 | 1 | 91 | 6 | 1 | 15 | 39 |
| 0022 | 3 | 6 | 97 | 73 | 30 | 46 | 38 | 3 | 2 | 86 | 8 | 3 | 55 | 36 |
| 0111 | 2 | 19 | 67 | 53 | 27 | 32 | 58 | 3 | 3 | 65 | 9 | 13 | 16 | 13 |
| 1200 | 2 | 48 | 27 | 62 | 12 | 95 | 78 | 1 | 4 | 50 | 27 | 39 | 0 | 1 |
| 1220 | 2 | 41 | 16 | 48 | 62 | 39 | 24 | 3 | 2 | 38 | 28 | 36 | 62 | 2 |
| 1221 | 2 | 24 | 54 | 71 | 48 | 84 | 53 | 1 | 2 | 18 | 27 | 35 | 59 | 13 |
| 0002 | 1 | 37 | 37 | 37 | 37 | 37 | 37 | 37 | 37 | 37 | 14 | 14 | 14 | 14 |
| 0101 | 1 | 39 | 39 | 39 | 39 | 39 | 39 | 39 | 39 | 39 | 8 | 8 | 8 | 8 |
| 0110 | 1 | 16 | 16 | 16 | 16 | 16 | 16 | 16 | 16 | 16 | 9 | 9 | 9 | 9 |
| 0121 | 1 | 55 | 55 | 55 | 55 | 55 | 55 | 55 | 55 | 55 | 20 | 20 | 20 | 20 |
| 1002 | 1 | 32 | 32 | 32 | 32 | 32 | 32 | 32 | 32 | 32 | 18 | 18 | 18 | 18 |
| 1112 | 1 | 42 | 42 | 42 | 42 | 42 | 42 | 42 | 42 | 42 | 21 | 21 | 21 | 21 |
| 1211 | 1 | 45 | 45 | 45 | 45 | 45 | 45 | 45 | 45 | 45 | 23 | 23 | 23 | 23 |
| 2122 | 1 | 37 | 37 | 37 | 37 | 37 | 37 | 37 | 37 | 37 | 41 | 41 | 41 | 41 |
| 2222 | 1 | 44 | 44 | 44 | 44 | 44 | 44 | 44 | 44 | 44 | 44 | 44 | 44 | 44 |
