# Supplementary material for: Robustness and Evolvability of the Human Signaling Network
Source: PLoS Comput Biol. 2014 Jul 31;10(7):e1003763. doi: 10.1371/journal.pcbi.1003763 (PMC4117429; doi:10.1371/journal.pcbi.1003763)
Supplement: Table S20 — The list of genes related to immune system that are included in the human signaling network. (DOC) [file pcbi.1003763.s038.doc]

**Table S20**. The list of genes related to immune system that are included in the human signaling network.

| EntrezGene ID | Gene symbol | Evolvability score | Robustness score |
| --- | --- | --- | --- |
| 998 | CDC42 | 0.667 | 0.333 |
| 5578 | PRKCA | 0.739 | 0.261 |
| 7454 | WAS | 0.222 | 0.778 |
| 207 | AKT1 | 0.833 | 0.167 |
| 399 | RHOH | 0.667 | 0.333 |
| 4690 | NCK1 | 0.667 | 0.333 |
| 8440 | NCK2 | 0.667 | 0.333 |
| 1399 | CRKL | 0.875 | 0.125 |
| 5336 | PLCG2 | 0.800 | 0.200 |
| 7189 | TRAF6 | 0.714 | 0.286 |
| 929 | CD14 | 0.889 | 0.111 |
| 10803 | CCR9 | 0.889 | 0.111 |
| 1230 | CCR1 | 0.889 | 0.111 |
| 1233 | CCR4 | 0.889 | 0.111 |
| 1234 | CCR5 | 0.889 | 0.111 |
| 1235 | CCR6 | 0.889 | 0.111 |
| 1236 | CCR7 | 0.889 | 0.111 |
| 1237 | CCR8 | 0.889 | 0.111 |
| 3579 | IL8RB | 0.889 | 0.111 |
| 643 | CXCR5 | 0.889 | 0.111 |
| 729230 | CCR2 | 0.889 | 0.111 |
| 7852 | CXCR4 | 0.889 | 0.111 |
| 1910 | EDNRB | 0.889 | 0.111 |
| 57105 | CYSLTR2 | 0.889 | 0.111 |
| 5724 | PTAFR | 0.889 | 0.111 |
| 3274 | HRH2 | 0.900 | 0.100 |
| 9564 | BCAR1 | 0.889 | 0.111 |
| 5594 | MAPK1 | 0.917 | 0.083 |
| 7132 | TNFRSF1A | 0.750 | 0.250 |
| 3554 | IL1R1 | 0.750 | 0.250 |
| 3556 | IL1RAP | 0.750 | 0.250 |
| 55361 | PI4K2A | 0.875 | 0.125 |
| 10344 | CCL26 | 1.000 | 0.000 |
| 10563 | CXCL13 | 1.000 | 0.000 |
| 10850 | CCL27 | 1.000 | 0.000 |
| 2919 | CXCL1 | 1.000 | 0.000 |
| 2920 | CXCL2 | 1.000 | 0.000 |
| 2921 | CXCL3 | 1.000 | 0.000 |
| 3576 | IL8 | 1.000 | 0.000 |
| 3627 | CXCL10 | 1.000 | 0.000 |
| 414062 | CCL3L3 | 1.000 | 0.000 |
| 4283 | CXCL9 | 1.000 | 0.000 |
| 5196 | PF4 | 1.000 | 0.000 |
| 5197 | PF4V1 | 1.000 | 0.000 |
| 5473 | PPBP | 1.000 | 0.000 |
| 56477 | CCL28 | 1.000 | 0.000 |
| 58191 | CXCL16 | 1.000 | 0.000 |
| 6346 | CCL1 | 1.000 | 0.000 |
| 6347 | CCL2 | 1.000 | 0.000 |
| 6348 | CCL3 | 1.000 | 0.000 |
| 6351 | CCL4 | 1.000 | 0.000 |
| 6352 | CCL5 | 1.000 | 0.000 |
| 6354 | CCL7 | 1.000 | 0.000 |
| 6355 | CCL8 | 1.000 | 0.000 |
| 6356 | CCL11 | 1.000 | 0.000 |
| 6357 | CCL13 | 1.000 | 0.000 |
| 6358 | CCL14 | 1.000 | 0.000 |
| 6359 | CCL15 | 1.000 | 0.000 |
| 6360 | CCL16 | 1.000 | 0.000 |
| 6361 | CCL17 | 1.000 | 0.000 |
| 6362 | CCL18 | 1.000 | 0.000 |
| 6363 | CCL19 | 1.000 | 0.000 |
| 6364 | CCL20 | 1.000 | 0.000 |
| 6366 | CCL21 | 1.000 | 0.000 |
| 6367 | CCL22 | 1.000 | 0.000 |
| 6368 | CCL23 | 1.000 | 0.000 |
| 6369 | CCL24 | 1.000 | 0.000 |
| 6370 | CCL25 | 1.000 | 0.000 |
| 6372 | CXCL6 | 1.000 | 0.000 |
| 6373 | CXCL11 | 1.000 | 0.000 |
| 6374 | CXCL5 | 1.000 | 0.000 |
| 6375 | XCL1 | 1.000 | 0.000 |
| 6376 | CX3CL1 | 1.000 | 0.000 |
| 6387 | CXCL12 | 1.000 | 0.000 |
| 6846 | XCL2 | 1.000 | 0.000 |
| 9547 | CXCL14 | 1.000 | 0.000 |
| 9560 | CCL4L1 | 1.000 | 0.000 |
| 55824 | PAG1 | 1.000 | 0.000 |
| 7057 | THBS1 | 1.000 | 0.000 |
| 7448 | VTN | 1.000 | 0.000 |
| 5871 | MAP4K2 | 1.000 | 0.000 |
| 7124 | TNF | 1.000 | 0.000 |
| 3552 | IL1A | 1.000 | 0.000 |
| 3553 | IL1B | 1.000 | 0.000 |
| 3655 | ITGA6 | 1.000 | 0.000 |
| 3672 | ITGA1 | 1.000 | 0.000 |
| 3676 | ITGA4 | 1.000 | 0.000 |
| 3688 | ITGB1 | 1.000 | 0.000 |
| 929 | CD14 | 1.000 | 0.000 |
| 10803 | CCR9 | 1.000 | 0.000 |
| 1230 | CCR1 | 1.000 | 0.000 |
| 1233 | CCR4 | 1.000 | 0.000 |
| 1234 | CCR5 | 1.000 | 0.000 |
| 1235 | CCR6 | 1.000 | 0.000 |
| 1236 | CCR7 | 1.000 | 0.000 |
| 1237 | CCR8 | 1.000 | 0.000 |
| 3579 | IL8RB | 1.000 | 0.000 |
| 643 | CXCR5 | 1.000 | 0.000 |
| 729230 | CCR2 | 1.000 | 0.000 |
| 7852 | CXCR4 | 1.000 | 0.000 |
| 1910 | EDNRB | 1.000 | 0.000 |
| 57105 | CYSLTR2 | 1.000 | 0.000 |
| 5724 | PTAFR | 1.000 | 0.000 |
| 3274 | HRH2 | 1.000 | 0.000 |
| 5578 | PRKCA | 1.000 | 0.000 |
| 5319 | PLA2G1B | 1.000 | 0.000 |
| 115727 | RASGRP4 | 1.000 | 0.000 |
| 5996 | RGS1 | 1.000 | 0.000 |
| 397 | ARHGDIB | 1.000 | 0.000 |
