# Supplementary material for: Robustness and Evolvability of the Human Signaling Network
Source: PLoS Comput Biol. 2014 Jul 31;10(7):e1003763. doi: 10.1371/journal.pcbi.1003763 (PMC4117429; doi:10.1371/journal.pcbi.1003763)
Supplement: Table S21 — The list of oncogenes that are included in the human signaling network. (DOC) [file pcbi.1003763.s039.doc]

**Table S21**. The list of oncogenes that are included in the human signaling network.

| EntrezGene ID | Gene symbol | Evolvability score | Robustness score |
| --- | --- | --- | --- |
| 1956 | EGFR | 0.750 | 0.250 |
| 207 | AKT1 | 0.833 | 0.167 |
| 3265 | HRAS | 0.636 | 0.364 |
| 3845 | KRAS | 0.636 | 0.364 |
| 4893 | NRAS | 0.636 | 0.364 |
| 1326 | MAP3K8 | 0.333 | 0.667 |
| 5290 | PIK3CA | 0.769 | 0.231 |
| 5894 | RAF1 | 0.500 | 0.500 |
| 5894 | RAF1 | 0.846 | 0.154 |
| 5781 | PTPN11 | 0.833 | 0.167 |
| 5894 | RAF1 | 1.000 | 0.000 |
| 5894 | RAF1 | 1.000 | 0.000 |
