# Supplementary material for: Robustness and Evolvability of the Human Signaling Network
Source: PLoS Comput Biol. 2014 Jul 31;10(7):e1003763. doi: 10.1371/journal.pcbi.1003763 (PMC4117429; doi:10.1371/journal.pcbi.1003763)
Supplement: Table S22 — The list of FDA-approved drug targets that are included in the human signaling network. (DOC) [file pcbi.1003763.s040.doc]

**Table S22**. The list of FDA-approved drug targets that are included in the human signaling network.

| EntrezGene ID | Gene symbol | Evolvability score | Robustness score |
| --- | --- | --- | --- |
| 6714 | SRC | 0.780 | 0.220 |
| 5879 | RAC1 | 0.542 | 0.458 |
| 5609 | MAP2K7 | 0.222 | 0.778 |
| 5578 | PRKCA | 0.739 | 0.261 |
| 5579 | PRKCB | 0.739 | 0.261 |
| 1956 | EGFR | 0.750 | 0.250 |
| 5515 | PPP2CA | 0.667 | 0.333 |
| 5516 | PPP2CB | 0.667 | 0.333 |
| 207 | AKT1 | 0.833 | 0.167 |
| 5337 | PLD1 | 0.600 | 0.400 |
| 5338 | PLD2 | 0.600 | 0.400 |
| 3265 | HRAS | 0.636 | 0.364 |
| 387 | RHOA | 0.667 | 0.333 |
| 5295 | PIK3R1 | 0.769 | 0.231 |
| 5770 | PTPN1 | 0.500 | 0.500 |
| 5894 | RAF1 | 0.500 | 0.500 |
| 58 | ACTA1 | 0.750 | 0.250 |
| 2885 | GRB2 | 0.800 | 0.200 |
| 5894 | RAF1 | 0.846 | 0.154 |
| 107 | ADCY1 | 0.833 | 0.167 |
| 113 | ADCY7 | 0.833 | 0.167 |
| 2149 | F2R | 0.889 | 0.111 |
| 1234 | CCR5 | 0.889 | 0.111 |
| 7852 | CXCR4 | 0.889 | 0.111 |
| 10800 | CYSLTR1 | 0.889 | 0.111 |
| 1128 | CHRM1 | 0.889 | 0.111 |
| 1129 | CHRM2 | 0.889 | 0.111 |
| 1131 | CHRM3 | 0.889 | 0.111 |
| 146 | ADRA1D | 0.889 | 0.111 |
| 147 | ADRA1B | 0.889 | 0.111 |
| 148 | ADRA1A | 0.889 | 0.111 |
| 185 | AGTR1 | 0.889 | 0.111 |
| 1909 | EDNRA | 0.889 | 0.111 |
| 1910 | EDNRB | 0.889 | 0.111 |
| 2149 | F2R | 0.889 | 0.111 |
| 2911 | GRM1 | 0.889 | 0.111 |
| 2915 | GRM5 | 0.889 | 0.111 |
| 3269 | HRH1 | 0.889 | 0.111 |
| 3356 | HTR2A | 0.889 | 0.111 |
| 3357 | HTR2B | 0.889 | 0.111 |
| 3358 | HTR2C | 0.889 | 0.111 |
| 3973 | LHCGR | 0.889 | 0.111 |
| 5021 | OXTR | 0.889 | 0.111 |
| 552 | AVPR1A | 0.889 | 0.111 |
| 553 | AVPR1B | 0.889 | 0.111 |
| 57105 | CYSLTR2 | 0.889 | 0.111 |
| 5731 | PTGER1 | 0.889 | 0.111 |
| 5733 | PTGER3 | 0.889 | 0.111 |
| 5737 | PTGFR | 0.889 | 0.111 |
| 624 | BDKRB2 | 0.889 | 0.111 |
| 6865 | TACR2 | 0.889 | 0.111 |
| 6869 | TACR1 | 0.889 | 0.111 |
| 6915 | TBXA2R | 0.889 | 0.111 |
| 886 | CCKAR | 0.889 | 0.111 |
| 887 | CCKBR | 0.889 | 0.111 |
| 1128 | CHRM1 | 0.900 | 0.100 |
| 1131 | CHRM3 | 0.900 | 0.100 |
| 1133 | CHRM5 | 0.900 | 0.100 |
| 135 | ADORA2A | 0.900 | 0.100 |
| 136 | ADORA2B | 0.900 | 0.100 |
| 153 | ADRB1 | 0.900 | 0.100 |
| 154 | ADRB2 | 0.900 | 0.100 |
| 155 | ADRB3 | 0.900 | 0.100 |
| 1812 | DRD1 | 0.900 | 0.100 |
| 1816 | DRD5 | 0.900 | 0.100 |
| 3274 | HRH2 | 0.900 | 0.100 |
| 3360 | HTR4 | 0.900 | 0.100 |
| 3362 | HTR6 | 0.900 | 0.100 |
| 3363 | HTR7 | 0.900 | 0.100 |
| 801 | CALM1 | 0.889 | 0.111 |
| 1950 | EGF | 0.500 | 0.500 |
| 5594 | MAPK1 | 0.917 | 0.083 |
| 5595 | MAPK3 | 0.917 | 0.083 |
| 54331 | GNG2 | 0.917 | 0.083 |
| 156 | ADRBK1 | 0.929 | 0.071 |
| 157 | ADRBK2 | 0.929 | 0.071 |
| 3554 | IL1R1 | 0.750 | 0.250 |
| 2147 | F2 | 1.000 | 0.000 |
| 3576 | IL8 | 1.000 | 0.000 |
| 5196 | PF4 | 1.000 | 0.000 |
| 6347 | CCL2 | 1.000 | 0.000 |
| 6368 | CCL23 | 1.000 | 0.000 |
| 6387 | CXCL12 | 1.000 | 0.000 |
| 1606 | DGKA | 1.000 | 0.000 |
| 1607 | DGKB | 1.000 | 0.000 |
| 1608 | DGKG | 1.000 | 0.000 |
| 8527 | DGKD | 1.000 | 0.000 |
| 1277 | COL1A1 | 1.000 | 0.000 |
| 1278 | COL1A2 | 1.000 | 0.000 |
| 1280 | COL2A1 | 1.000 | 0.000 |
| 1281 | COL3A1 | 1.000 | 0.000 |
| 1291 | COL6A1 | 1.000 | 0.000 |
| 2335 | FN1 | 1.000 | 0.000 |
| 284217 | LAMA1 | 1.000 | 0.000 |
| 3909 | LAMA3 | 1.000 | 0.000 |
| 3911 | LAMA5 | 1.000 | 0.000 |
| 3912 | LAMB1 | 1.000 | 0.000 |
| 3915 | LAMC1 | 1.000 | 0.000 |
| 7057 | THBS1 | 1.000 | 0.000 |
| 7448 | VTN | 1.000 | 0.000 |
| 7450 | VWF | 1.000 | 0.000 |
| 54331 | GNG2 | 1.000 | 0.000 |
| 54331 | GNG2 | 1.000 | 0.000 |
| 54331 | GNG2 | 1.000 | 0.000 |
| 7124 | TNF | 1.000 | 0.000 |
| 3553 | IL1B | 1.000 | 0.000 |
| 22801 | ITGA11 | 1.000 | 0.000 |
| 3674 | ITGA2B | 1.000 | 0.000 |
| 3676 | ITGA4 | 1.000 | 0.000 |
| 3690 | ITGB3 | 1.000 | 0.000 |
| 2149 | F2R | 1.000 | 0.000 |
| 1234 | CCR5 | 1.000 | 0.000 |
| 7852 | CXCR4 | 1.000 | 0.000 |
| 10800 | CYSLTR1 | 1.000 | 0.000 |
| 1128 | CHRM1 | 1.000 | 0.000 |
| 1129 | CHRM2 | 1.000 | 0.000 |
| 1131 | CHRM3 | 1.000 | 0.000 |
| 146 | ADRA1D | 1.000 | 0.000 |
| 147 | ADRA1B | 1.000 | 0.000 |
| 148 | ADRA1A | 1.000 | 0.000 |
| 185 | AGTR1 | 1.000 | 0.000 |
| 1909 | EDNRA | 1.000 | 0.000 |
| 1910 | EDNRB | 1.000 | 0.000 |
| 2149 | F2R | 1.000 | 0.000 |
| 2911 | GRM1 | 1.000 | 0.000 |
| 2915 | GRM5 | 1.000 | 0.000 |
| 3269 | HRH1 | 1.000 | 0.000 |
| 3356 | HTR2A | 1.000 | 0.000 |
| 3357 | HTR2B | 1.000 | 0.000 |
| 3358 | HTR2C | 1.000 | 0.000 |
| 3973 | LHCGR | 1.000 | 0.000 |
| 5021 | OXTR | 1.000 | 0.000 |
| 552 | AVPR1A | 1.000 | 0.000 |
| 553 | AVPR1B | 1.000 | 0.000 |
| 57105 | CYSLTR2 | 1.000 | 0.000 |
| 5731 | PTGER1 | 1.000 | 0.000 |
| 5733 | PTGER3 | 1.000 | 0.000 |
| 5737 | PTGFR | 1.000 | 0.000 |
| 624 | BDKRB2 | 1.000 | 0.000 |
| 6865 | TACR2 | 1.000 | 0.000 |
| 6869 | TACR1 | 1.000 | 0.000 |
| 6915 | TBXA2R | 1.000 | 0.000 |
| 886 | CCKAR | 1.000 | 0.000 |
| 887 | CCKBR | 1.000 | 0.000 |
| 1128 | CHRM1 | 1.000 | 0.000 |
| 1131 | CHRM3 | 1.000 | 0.000 |
| 1133 | CHRM5 | 1.000 | 0.000 |
| 135 | ADORA2A | 1.000 | 0.000 |
| 136 | ADORA2B | 1.000 | 0.000 |
| 153 | ADRB1 | 1.000 | 0.000 |
| 154 | ADRB2 | 1.000 | 0.000 |
| 155 | ADRB3 | 1.000 | 0.000 |
| 1812 | DRD1 | 1.000 | 0.000 |
| 1816 | DRD5 | 1.000 | 0.000 |
| 3274 | HRH2 | 1.000 | 0.000 |
| 3360 | HTR4 | 1.000 | 0.000 |
| 3362 | HTR6 | 1.000 | 0.000 |
| 3363 | HTR7 | 1.000 | 0.000 |
| 5141 | PDE4A | 1.000 | 0.000 |
| 5142 | PDE4B | 1.000 | 0.000 |
| 5143 | PDE4C | 1.000 | 0.000 |
| 5144 | PDE4D | 1.000 | 0.000 |
| 5578 | PRKCA | 1.000 | 0.000 |
| 5579 | PRKCB | 1.000 | 0.000 |
| 5321 | PLA2G4A | 1.000 | 0.000 |
| 8398 | PLA2G6 | 1.000 | 0.000 |
| 5894 | RAF1 | 1.000 | 0.000 |
