# Supplementary material for: Robustness and Evolvability of the Human Signaling Network
Source: PLoS Comput Biol. 2014 Jul 31;10(7):e1003763. doi: 10.1371/journal.pcbi.1003763 (PMC4117429; doi:10.1371/journal.pcbi.1003763)
Supplement: Table S23 — The list of experimental drug targets that are included in the human signaling network. (DOC) [file pcbi.1003763.s041.doc]

**Table S23**. The list of experimental drug targets that are included in the human signaling network.

| EntrezGene ID | Gene symbol | Evolvability score | Robustness score |
| --- | --- | --- | --- |
| 6714 | SRC | 0.780 | 0.220 |
| 998 | CDC42 | 0.667 | 0.333 |
| 5879 | RAC1 | 0.542 | 0.458 |
| 4629 | MYH11 | 0.250 | 0.750 |
| 1432 | MAPK14 | 0.250 | 0.750 |
| 6300 | MAPK12 | 0.250 | 0.750 |
| 7454 | WAS | 0.222 | 0.778 |
| 1956 | EGFR | 0.750 | 0.250 |
| 5501 | PPP1CC | 0.143 | 0.857 |
| 4294 | MAP3K10 | 0.167 | 0.833 |
| 207 | AKT1 | 0.833 | 0.167 |
| 5566 | PRKACA | 0.737 | 0.263 |
| 387 | RHOA | 0.667 | 0.333 |
| 5747 | PTK2 | 0.778 | 0.222 |
| 5294 | PIK3CG | 0.769 | 0.231 |
| 5770 | PTPN1 | 0.500 | 0.500 |
| 5898 | RALA | 0.400 | 0.600 |
| 58 | ACTA1 | 0.750 | 0.250 |
| 1445 | CSK | 0.778 | 0.222 |
| 113 | ADCY7 | 0.833 | 0.167 |
| 1128 | CHRM1 | 0.889 | 0.111 |
| 148 | ADRA1A | 0.889 | 0.111 |
| 3356 | HTR2A | 0.889 | 0.111 |
| 1128 | CHRM1 | 0.900 | 0.100 |
| 3362 | HTR6 | 0.900 | 0.100 |
| 801 | CALM1 | 0.889 | 0.111 |
| 9564 | BCAR1 | 0.889 | 0.111 |
| 5594 | MAPK1 | 0.917 | 0.083 |
| 5595 | MAPK3 | 0.917 | 0.083 |
| 7132 | TNFRSF1A | 0.750 | 0.250 |
| 5604 | MAP2K1 | 0.889 | 0.111 |
| 5781 | PTPN11 | 0.833 | 0.167 |
| 2147 | F2 | 1.000 | 0.000 |
| 3627 | CXCL10 | 1.000 | 0.000 |
| 5473 | PPBP | 1.000 | 0.000 |
| 6352 | CCL5 | 1.000 | 0.000 |
| 6355 | CCL8 | 1.000 | 0.000 |
| 6364 | CCL20 | 1.000 | 0.000 |
| 1311 | COMP | 1.000 | 0.000 |
| 7057 | THBS1 | 1.000 | 0.000 |
| 3691 | ITGB4 | 1.000 | 0.000 |
| 1128 | CHRM1 | 1.000 | 0.000 |
| 148 | ADRA1A | 1.000 | 0.000 |
| 3356 | HTR2A | 1.000 | 0.000 |
| 1128 | CHRM1 | 1.000 | 0.000 |
| 3362 | HTR6 | 1.000 | 0.000 |
| 5142 | PDE4B | 1.000 | 0.000 |
| 5144 | PDE4D | 1.000 | 0.000 |
| 5319 | PLA2G1B | 1.000 | 0.000 |
| 5320 | PLA2G2A | 1.000 | 0.000 |
| 8399 | PLA2G10 | 1.000 | 0.000 |
| 5037 | PEBP1 | 1.000 | 0.000 |
