# Supplementary material for: Robustness and Evolvability of the Human Signaling Network
Source: PLoS Comput Biol. 2014 Jul 31;10(7):e1003763. doi: 10.1371/journal.pcbi.1003763 (PMC4117429; doi:10.1371/journal.pcbi.1003763)
Supplement: Table S24 — The list of genes related to receptors that are included in the human signaling network. (DOC) [file pcbi.1003763.s042.doc]

**Table S24**. The list of genes related to receptors that are included in the human signaling network.

| EntrezGene ID | Gene symbol | Evolvability score | Robustness score |
| --- | --- | --- | --- |
| 1956 | EGFR | 0.750 | 0.250 |
| 7182 | NR2C2 | 0.200 | 0.800 |
| 2149 | F2R | 0.889 | 0.111 |
| 929 | CD14 | 0.889 | 0.111 |
| 10663 | CXCR6 | 0.889 | 0.111 |
| 10803 | CCR9 | 0.889 | 0.111 |
| 1230 | CCR1 | 0.889 | 0.111 |
| 1232 | CCR3 | 0.889 | 0.111 |
| 1233 | CCR4 | 0.889 | 0.111 |
| 1234 | CCR5 | 0.889 | 0.111 |
| 1235 | CCR6 | 0.889 | 0.111 |
| 1236 | CCR7 | 0.889 | 0.111 |
| 1237 | CCR8 | 0.889 | 0.111 |
| 1524 | CX3CR1 | 0.889 | 0.111 |
| 2826 | CCR10 | 0.889 | 0.111 |
| 2829 | XCR1 | 0.889 | 0.111 |
| 2833 | CXCR3 | 0.889 | 0.111 |
| 3577 | IL8RA | 0.889 | 0.111 |
| 3579 | IL8RB | 0.889 | 0.111 |
| 643 | CXCR5 | 0.889 | 0.111 |
| 729230 | CCR2 | 0.889 | 0.111 |
| 7852 | CXCR4 | 0.889 | 0.111 |
| 10800 | CYSLTR1 | 0.889 | 0.111 |
| 1128 | CHRM1 | 0.889 | 0.111 |
| 1129 | CHRM2 | 0.889 | 0.111 |
| 1131 | CHRM3 | 0.889 | 0.111 |
| 146 | ADRA1D | 0.889 | 0.111 |
| 147 | ADRA1B | 0.889 | 0.111 |
| 148 | ADRA1A | 0.889 | 0.111 |
| 185 | AGTR1 | 0.889 | 0.111 |
| 1909 | EDNRA | 0.889 | 0.111 |
| 1910 | EDNRB | 0.889 | 0.111 |
| 2149 | F2R | 0.889 | 0.111 |
| 2911 | GRM1 | 0.889 | 0.111 |
| 2915 | GRM5 | 0.889 | 0.111 |
| 2925 | GRPR | 0.889 | 0.111 |
| 3269 | HRH1 | 0.889 | 0.111 |
| 3356 | HTR2A | 0.889 | 0.111 |
| 3357 | HTR2B | 0.889 | 0.111 |
| 3358 | HTR2C | 0.889 | 0.111 |
| 3973 | LHCGR | 0.889 | 0.111 |
| 4923 | NTSR1 | 0.889 | 0.111 |
| 5021 | OXTR | 0.889 | 0.111 |
| 552 | AVPR1A | 0.889 | 0.111 |
| 553 | AVPR1B | 0.889 | 0.111 |
| 56413 | LTB4R2 | 0.889 | 0.111 |
| 57105 | CYSLTR2 | 0.889 | 0.111 |
| 5724 | PTAFR | 0.889 | 0.111 |
| 5731 | PTGER1 | 0.889 | 0.111 |
| 5733 | PTGER3 | 0.889 | 0.111 |
| 5737 | PTGFR | 0.889 | 0.111 |
| 623 | BDKRB1 | 0.889 | 0.111 |
| 624 | BDKRB2 | 0.889 | 0.111 |
| 6865 | TACR2 | 0.889 | 0.111 |
| 6869 | TACR1 | 0.889 | 0.111 |
| 6870 | TACR3 | 0.889 | 0.111 |
| 6915 | TBXA2R | 0.889 | 0.111 |
| 7201 | TRHR | 0.889 | 0.111 |
| 886 | CCKAR | 0.889 | 0.111 |
| 887 | CCKBR | 0.889 | 0.111 |
| 1128 | CHRM1 | 0.900 | 0.100 |
| 1131 | CHRM3 | 0.900 | 0.100 |
| 1133 | CHRM5 | 0.900 | 0.100 |
| 135 | ADORA2A | 0.900 | 0.100 |
| 136 | ADORA2B | 0.900 | 0.100 |
| 153 | ADRB1 | 0.900 | 0.100 |
| 154 | ADRB2 | 0.900 | 0.100 |
| 155 | ADRB3 | 0.900 | 0.100 |
| 1812 | DRD1 | 0.900 | 0.100 |
| 1816 | DRD5 | 0.900 | 0.100 |
| 3274 | HRH2 | 0.900 | 0.100 |
| 3360 | HTR4 | 0.900 | 0.100 |
| 3361 | HTR5A | 0.900 | 0.100 |
| 3362 | HTR6 | 0.900 | 0.100 |
| 3363 | HTR7 | 0.900 | 0.100 |
| 7132 | TNFRSF1A | 0.750 | 0.250 |
| 3554 | IL1R1 | 0.750 | 0.250 |
| 3556 | IL1RAP | 0.750 | 0.250 |
| 3708 | ITPR1 | 0.889 | 0.111 |
| 2147 | F2 | 1.000 | 0.000 |
| 58191 | CXCL16 | 1.000 | 0.000 |
| 3911 | LAMA5 | 1.000 | 0.000 |
| 7448 | VTN | 1.000 | 0.000 |
| 22801 | ITGA11 | 1.000 | 0.000 |
| 3655 | ITGA6 | 1.000 | 0.000 |
| 3672 | ITGA1 | 1.000 | 0.000 |
| 3673 | ITGA2 | 1.000 | 0.000 |
| 3674 | ITGA2B | 1.000 | 0.000 |
| 3675 | ITGA3 | 1.000 | 0.000 |
| 3676 | ITGA4 | 1.000 | 0.000 |
| 3678 | ITGA5 | 1.000 | 0.000 |
| 3679 | ITGA7 | 1.000 | 0.000 |
| 3680 | ITGA9 | 1.000 | 0.000 |
| 3685 | ITGAV | 1.000 | 0.000 |
| 8515 | ITGA10 | 1.000 | 0.000 |
| 8516 | ITGA8 | 1.000 | 0.000 |
| 3688 | ITGB1 | 1.000 | 0.000 |
| 3690 | ITGB3 | 1.000 | 0.000 |
| 3691 | ITGB4 | 1.000 | 0.000 |
| 3693 | ITGB5 | 1.000 | 0.000 |
| 3694 | ITGB6 | 1.000 | 0.000 |
| 3695 | ITGB7 | 1.000 | 0.000 |
| 3696 | ITGB8 | 1.000 | 0.000 |
| 2149 | F2R | 1.000 | 0.000 |
| 929 | CD14 | 1.000 | 0.000 |
| 10663 | CXCR6 | 1.000 | 0.000 |
| 10803 | CCR9 | 1.000 | 0.000 |
| 1230 | CCR1 | 1.000 | 0.000 |
| 1232 | CCR3 | 1.000 | 0.000 |
| 1233 | CCR4 | 1.000 | 0.000 |
| 1234 | CCR5 | 1.000 | 0.000 |
| 1235 | CCR6 | 1.000 | 0.000 |
| 1236 | CCR7 | 1.000 | 0.000 |
| 1237 | CCR8 | 1.000 | 0.000 |
| 1524 | CX3CR1 | 1.000 | 0.000 |
| 2826 | CCR10 | 1.000 | 0.000 |
| 2829 | XCR1 | 1.000 | 0.000 |
| 2833 | CXCR3 | 1.000 | 0.000 |
| 3577 | IL8RA | 1.000 | 0.000 |
| 3579 | IL8RB | 1.000 | 0.000 |
| 643 | CXCR5 | 1.000 | 0.000 |
| 729230 | CCR2 | 1.000 | 0.000 |
| 7852 | CXCR4 | 1.000 | 0.000 |
| 10800 | CYSLTR1 | 1.000 | 0.000 |
| 1128 | CHRM1 | 1.000 | 0.000 |
| 1129 | CHRM2 | 1.000 | 0.000 |
| 1131 | CHRM3 | 1.000 | 0.000 |
| 146 | ADRA1D | 1.000 | 0.000 |
| 147 | ADRA1B | 1.000 | 0.000 |
| 148 | ADRA1A | 1.000 | 0.000 |
| 185 | AGTR1 | 1.000 | 0.000 |
| 1909 | EDNRA | 1.000 | 0.000 |
| 1910 | EDNRB | 1.000 | 0.000 |
| 2149 | F2R | 1.000 | 0.000 |
| 2911 | GRM1 | 1.000 | 0.000 |
| 2915 | GRM5 | 1.000 | 0.000 |
| 2925 | GRPR | 1.000 | 0.000 |
| 3269 | HRH1 | 1.000 | 0.000 |
| 3356 | HTR2A | 1.000 | 0.000 |
| 3357 | HTR2B | 1.000 | 0.000 |
| 3358 | HTR2C | 1.000 | 0.000 |
| 3973 | LHCGR | 1.000 | 0.000 |
| 4923 | NTSR1 | 1.000 | 0.000 |
| 5021 | OXTR | 1.000 | 0.000 |
| 552 | AVPR1A | 1.000 | 0.000 |
| 553 | AVPR1B | 1.000 | 0.000 |
| 56413 | LTB4R2 | 1.000 | 0.000 |
| 57105 | CYSLTR2 | 1.000 | 0.000 |
| 5724 | PTAFR | 1.000 | 0.000 |
| 5731 | PTGER1 | 1.000 | 0.000 |
| 5733 | PTGER3 | 1.000 | 0.000 |
| 5737 | PTGFR | 1.000 | 0.000 |
| 623 | BDKRB1 | 1.000 | 0.000 |
| 624 | BDKRB2 | 1.000 | 0.000 |
| 6865 | TACR2 | 1.000 | 0.000 |
| 6869 | TACR1 | 1.000 | 0.000 |
| 6870 | TACR3 | 1.000 | 0.000 |
| 6915 | TBXA2R | 1.000 | 0.000 |
| 7201 | TRHR | 1.000 | 0.000 |
| 886 | CCKAR | 1.000 | 0.000 |
| 887 | CCKBR | 1.000 | 0.000 |
| 1128 | CHRM1 | 1.000 | 0.000 |
| 1131 | CHRM3 | 1.000 | 0.000 |
| 1133 | CHRM5 | 1.000 | 0.000 |
| 135 | ADORA2A | 1.000 | 0.000 |
| 136 | ADORA2B | 1.000 | 0.000 |
| 153 | ADRB1 | 1.000 | 0.000 |
| 154 | ADRB2 | 1.000 | 0.000 |
| 155 | ADRB3 | 1.000 | 0.000 |
| 1812 | DRD1 | 1.000 | 0.000 |
| 1816 | DRD5 | 1.000 | 0.000 |
| 3274 | HRH2 | 1.000 | 0.000 |
| 3360 | HTR4 | 1.000 | 0.000 |
| 3361 | HTR5A | 1.000 | 0.000 |
| 3362 | HTR6 | 1.000 | 0.000 |
| 3363 | HTR7 | 1.000 | 0.000 |
| 5786 | PTPRA | 1.000 | 0.000 |
