# Supplementary material for: Robustness and Evolvability of the Human Signaling Network
Source: PLoS Comput Biol. 2014 Jul 31;10(7):e1003763. doi: 10.1371/journal.pcbi.1003763 (PMC4117429; doi:10.1371/journal.pcbi.1003763)
Supplement: Table S25 — The list of genes related to kinases that are included in the human signaling network. (DOC) [file pcbi.1003763.s043.doc]

**Table S25**. The list of genes related to kinases that are included in the human signaling network.

| EntrezGene ID | Gene symbol | Evolvability score | Robustness score |
| --- | --- | --- | --- |
| 5606 | MAP2K3 | 0.083 | 0.917 |
| 6416 | MAP2K4 | 0.167 | 0.833 |
| 10746 | MAP3K2 | 0.200 | 0.800 |
| 6714 | SRC | 0.780 | 0.220 |
| 4214 | MAP3K1 | 0.500 | 0.500 |
| 5608 | MAP2K6 | 0.125 | 0.875 |
| 5609 | MAP2K7 | 0.222 | 0.778 |
| 1432 | MAPK14 | 0.250 | 0.750 |
| 5600 | MAPK11 | 0.250 | 0.750 |
| 5603 | MAPK13 | 0.250 | 0.750 |
| 6300 | MAPK12 | 0.250 | 0.750 |
| 5578 | PRKCA | 0.739 | 0.261 |
| 5579 | PRKCB | 0.739 | 0.261 |
| 5582 | PRKCG | 0.739 | 0.261 |
| 1956 | EGFR | 0.750 | 0.250 |
| 4296 | MAP3K11 | 0.143 | 0.857 |
| 10298 | PAK4 | 0.789 | 0.211 |
| 5058 | PAK1 | 0.789 | 0.211 |
| 5062 | PAK2 | 0.789 | 0.211 |
| 5063 | PAK3 | 0.789 | 0.211 |
| 56924 | PAK6 | 0.789 | 0.211 |
| 57144 | PAK7 | 0.789 | 0.211 |
| 4215 | MAP3K3 | 0.250 | 0.750 |
| 4216 | MAP3K4 | 0.167 | 0.833 |
| 4294 | MAP3K10 | 0.167 | 0.833 |
| 10000 | AKT3 | 0.833 | 0.167 |
| 207 | AKT1 | 0.833 | 0.167 |
| 208 | AKT2 | 0.833 | 0.167 |
| 4638 | MYLK | 0.200 | 0.800 |
| 85366 | MYLK2 | 0.200 | 0.800 |
| 91807 | MYLK3 | 0.200 | 0.800 |
| 4293 | MAP3K9 | 0.200 | 0.800 |
| 5566 | PRKACA | 0.737 | 0.263 |
| 5567 | PRKACB | 0.737 | 0.263 |
| 5568 | PRKACG | 0.737 | 0.263 |
| 1326 | MAP3K8 | 0.333 | 0.667 |
| 814 | CAMK4 | 0.500 | 0.500 |
| 815 | CAMK2A | 0.500 | 0.500 |
| 816 | CAMK2B | 0.500 | 0.500 |
| 817 | CAMK2D | 0.500 | 0.500 |
| 818 | CAMK2G | 0.500 | 0.500 |
| 5747 | PTK2 | 0.778 | 0.222 |
| 9020 | MAP3K14 | 0.250 | 0.750 |
| 5163 | PDK1 | 0.750 | 0.250 |
| 5290 | PIK3CA | 0.769 | 0.231 |
| 5291 | PIK3CB | 0.769 | 0.231 |
| 5293 | PIK3CD | 0.769 | 0.231 |
| 5294 | PIK3CG | 0.769 | 0.231 |
| 5296 | PIK3R2 | 0.769 | 0.231 |
| 8503 | PIK3R3 | 0.769 | 0.231 |
| 200576 | PIKFYVE | 0.769 | 0.231 |
| 23396 | PIP5K1C | 0.769 | 0.231 |
| 5305 | PIP4K2A | 0.769 | 0.231 |
| 79837 | PIP4K2C | 0.769 | 0.231 |
| 8394 | PIP5K1A | 0.769 | 0.231 |
| 8395 | PIP5K1B | 0.769 | 0.231 |
| 8396 | PIP4K2B | 0.769 | 0.231 |
| 5894 | RAF1 | 0.500 | 0.500 |
| 10645 | CAMKK2 | 0.750 | 0.250 |
| 84254 | CAMKK1 | 0.750 | 0.250 |
| 1399 | CRKL | 0.875 | 0.125 |
| 1445 | CSK | 0.778 | 0.222 |
| 3611 | ILK | 0.667 | 0.333 |
| 5894 | RAF1 | 0.846 | 0.154 |
| 6011 | GRK1 | 0.500 | 0.500 |
| 5601 | MAPK9 | 0.571 | 0.429 |
| 51347 | TAOK3 | 0.333 | 0.667 |
| 57551 | TAOK1 | 0.333 | 0.667 |
| 9344 | TAOK2 | 0.333 | 0.667 |
| 4217 | MAP3K5 | 0.667 | 0.333 |
| 5594 | MAPK1 | 0.917 | 0.083 |
| 5595 | MAPK3 | 0.917 | 0.083 |
| 156 | ADRBK1 | 0.929 | 0.071 |
| 131890 | GRK7 | 0.929 | 0.071 |
| 157 | ADRBK2 | 0.929 | 0.071 |
| 2868 | GRK4 | 0.929 | 0.071 |
| 2869 | GRK5 | 0.929 | 0.071 |
| 2870 | GRK6 | 0.929 | 0.071 |
| 6011 | GRK1 | 0.929 | 0.071 |
| 5604 | MAP2K1 | 0.889 | 0.111 |
| 5605 | MAP2K2 | 0.889 | 0.111 |
| 5297 | PI4KA | 0.875 | 0.125 |
| 5298 | PI4KB | 0.875 | 0.125 |
| 55300 | PI4K2B | 0.875 | 0.125 |
| 55361 | PI4K2A | 0.875 | 0.125 |
| 6347 | CCL2 | 1.000 | 0.000 |
| 6351 | CCL4 | 1.000 | 0.000 |
| 1606 | DGKA | 1.000 | 0.000 |
| 1607 | DGKB | 1.000 | 0.000 |
| 1608 | DGKG | 1.000 | 0.000 |
| 160851 | DGKH | 1.000 | 0.000 |
| 1609 | DGKQ | 1.000 | 0.000 |
| 8525 | DGKZ | 1.000 | 0.000 |
| 8526 | DGKE | 1.000 | 0.000 |
| 8527 | DGKD | 1.000 | 0.000 |
| 9162 | DGKI | 1.000 | 0.000 |
| 5649 | RELN | 1.000 | 0.000 |
| 5871 | MAP4K2 | 1.000 | 0.000 |
| 27330 | RPS6KA6 | 1.000 | 0.000 |
| 6195 | RPS6KA1 | 1.000 | 0.000 |
| 6196 | RPS6KA2 | 1.000 | 0.000 |
| 6197 | RPS6KA3 | 1.000 | 0.000 |
| 5578 | PRKCA | 1.000 | 0.000 |
| 5579 | PRKCB | 1.000 | 0.000 |
| 5582 | PRKCG | 1.000 | 0.000 |
| 5894 | RAF1 | 1.000 | 0.000 |
| 5894 | RAF1 | 1.000 | 0.000 |
